# Phenotypes and rates of cancer-relevant symptoms and tests in the year before cancer diagnosis in UK Biobank and CPRD Gold

**DOI:** 10.1371/journal.pdig.0000383

**Published:** 2023-12-15

**Authors:** Matthew Barclay, Cristina Renzi, Antonis Antoniou, Spiros Denaxas, Hannah Harrison, Samantha Ip, Nora Pashayan, Ana Torralbo, Juliet Usher-Smith, Angela Wood, Georgios Lyratzopoulos

**Affiliations:** 1 Department of Behavioural Science and Health, Institute of Epidemiology and Healthcare, University College London, London, United Kingdom; 2 Faculty of Medicine, University Vita-Salute San Raffaele, Milan, Italy; 3 Department of Public Health and Primary Care, School of Clinical Medicine, University of Cambridge, Cambridge, United Kingdom; 4 Institute of Health Informatics, University College London, London, United Kingdom; 5 Victor Phillip Dahdaleh Heart and Lung Research Institute, University of Cambridge, Cambridge, United Kingdom; 6 Department of Applied Health Research, Institute of Epidemiology and Healthcare, University College London, London, United Kingdom; 7 British Heart Foundation Centre of Research Excellence, University of Cambridge, Cambridge, United Kingdom; 8 National Institute for Health and Care Research Blood and Transplant Research Unit in Donor Health and Behaviour, University of Cambridge, Cambridge, United Kingdom; 9 Health Data Research UK Cambridge, Wellcome Genome Campus and University of Cambridge, Cambridge, United Kingdom; 10 Cambridge Centre for Artificial Intelligence in Medicine, University of Cambridge, Cambridge, United Kingdom; Massachusetts General Hospital, UNITED STATES

## Abstract

Early diagnosis of cancer relies on accurate assessment of cancer risk in patients presenting with symptoms, when screening is not appropriate. But recorded symptoms in cancer patients pre-diagnosis may vary between different sources of electronic health records (EHRs), either genuinely or due to differential completeness of symptom recording. To assess possible differences, we analysed primary care EHRs in the year pre-diagnosis of cancer in UK Biobank and Clinical Practice Research Datalink (CPRD) populations linked to cancer registry data. We developed harmonised phenotypes in Read v2 and CTV3 coding systems for 21 symptoms and eight blood tests relevant to cancer diagnosis. Among 22,601 CPRD and 11,594 UK Biobank cancer patients, 54% and 36%, respectively, had at least one consultation for possible cancer symptoms recorded in the year before their diagnosis. Adjusted comparisons between datasets were made using multivariable Poisson models, comparing rates of symptoms/tests in CPRD against expected rates if cancer site-age-sex-deprivation associations were the same as in UK Biobank. UK Biobank cancer patients compared with those in CPRD had lower rates of consultation for possible cancer symptoms [RR: 0.61 (0.59–0.63)], and lower rates for any primary care consultation [RR: 0.86 (95%CI 0.85–0.87)]. Differences were larger for ‘non-alarm’ symptoms [RR: 0.54 (0.52–0.56)], and smaller for ‘alarm’ symptoms [RR: 0.80 (0.76–0.84)] and blood tests [RR: 0.93 (0.90–0.95)]. In the CPRD cohort, approximately representative of the UK population, half of cancer patients had recorded symptoms in the year before diagnosis. The frequency of non-specific presenting symptoms recorded in the year pre-diagnosis of cancer was substantially lower among UK Biobank participants. The degree to which results based on highly selected biobank cohorts are generalisable needs to be examined in disease-specific contexts.

## Author summary

We develop symptom phenotypes and describe and compare rates of symptoms and blood tests before cancer diagnosis in patients drawn from a representative sample and from UK Biobank. We found that in the representative sample around half of patients had recorded symptoms in the year before cancer diagnosis, but that rates of recorded symptoms in UK Biobank participants were substantially lower. These differences primarily related to non-specific symptoms, but remained following adjustment for cancer site, age, sex, and socio-economic deprivation. The phenotypes for identifying symptoms we developed may be useful for other researchers working with UK primary care data. The differences in pre-diagnostic symptoms emphasise that the generalisability of results from cohort studies need to be examined in a disease-specific context, and support efforts for ensuring equitable participation in major cohort studies.

## Introduction

Early diagnosis is an important strategy for cancer control associated with improved clinical outcomes and patient experience [1–3]. Over 90% of patients in the UK and over 80% in the US are diagnosed after symptom onset [4–6], and understanding the risk of underlying cancer in symptomatic patients presenting to primary care is critical for achieving earlier diagnosis. Symptoms of possible cancer fall into two groups. First, so-called ’alarm’ or ’red-flag’ symptoms, for which national guidelines (for example in England and Scotland [7]) indicate that cancer should be suspected, and appropriate referral actions be considered. Overall, the positive predictive value of those symptoms exceeds 3% [8], which is deemed by the UK National Institute of Health and Care Excellence (NICE) to represent an appropriate risk threshold for fast-track investigation. Second, other symptoms with overall predictive value for cancer that is lower than 3%; around half of patients with cancer initially present with such non-specific or vague symptoms [9]. Yet positive predictive values are affected by the underlying risk, and values are generally substantially greater in older than younger individuals and men over women.

Early diagnosis strategies rely on primary care physicians being able to appropriately assess the risk of underlying cancer in symptomatic patients [10]. Several reviews and national guidelines summarise the evidence about the predictive value of common symptoms in either category and related positive predictive values [10–12]. Additionally, risk prediction models with a web interface exist that can support individualised risk prediction conditional on specific symptoms [13]. Currently available evidence chiefly pertains to sources of electronic health records data that do not include information on genetic risk [2,14–18]. The UK Biobank is one of the largest data sources where information from electronic health records and genetic risk are integrated. There is great interest in examining the contribution of genetic predisposition on cancer risk additional to other characteristics using UK Biobank data. Enabling such research requires development of appropriate phenotypes for symptoms of interest, and their distribution described and compared against other sources to guide interpretation. These considerations have motivated our study.

Many EHR datasets are representative of the reference population, including CPRD Gold [19]. Others cover a highly-selected cohort of patients, including current biobank cohorts such as UK Biobank. These selected cohorts are attractive for research due to the availability of linked genetic information and rich data on exposures during life-course in addition to EHR data on participants’ healthcare use. However, the prevalence of health conditions can differ between EHR sources (e.g., [20,21]), either because of differing patient populations or because of differential completeness in the recording of these conditions in participating practices and EHR information systems used. UK Biobank participant health outcomes and demographic characteristics differ from the general population [22], and the impact these differences on the symptoms and tests in the year before a cancer diagnosis is unclear.

Given the above, this study had three aims. First, to develop harmonised symptom and blood test phenotypes for Read v2 and CTV3 coding systems. Second, to estimate the prevalence of 21 symptoms and eight blood tests in the year before cancer diagnosis in a representative cohort from CPRD Gold. Third, to compare rates of symptoms and blood tests in the year before diagnosis in cancer patients in CPRD and UK Biobank.

## Methods

The analysis had three main components: identifying comparable cohorts of cancer patients in UK Biobank and CPRD; identifying the same types of outcome events in each cohort; and calculating rates and adjusted comparisons between the cohorts.

### Data sources

CPRD Gold is an electronic health record (EHR) database of fully-coded primary care records from practices across the UK using the Vision software [19], with linkage to additional datasets including cancer registration data. Information on primary care consultations forms an inherent part of the EHR and is coded using Read v2, a clinical coding system primarily developed for primary care that has been used since 1985 [23]. The database contains data on more than 11 million patients from 1987 onwards [19], with an average follow-up of 5.1 years. Use of CPRD data in this study was approved by the UK Medicines and Healthcare products Regulatory Agency (MHRA) Independent Scientific Advisory Committee (ISAC Protocol number 18_299RMnA5), under Section 251 (NHS Social Care Act 2006).

UK Biobank is a prospective cohort study of around 500,000 people [24], recruited between the ages of 40 and 69 in 2006–2010. Around 200,000 UK Biobank participants have linked routinely collected primary care EHRs, recorded using either TPP (England, CTV3 coding, 71% of records), EMIS (Wales and Scotland, Read v2 coding, 20%), or Vision (England, Read v2 coding, 10%) systems. EHR data in UK Biobank are available from both before and after participants’ recruitment to the study. Data from the UK Biobank Resource was provided under application number 64351; UK Biobank received ethical approval from the National Information Governance Board for Health and Social Care and the National Health Service North West Centre for Research Ethics Committee (Ref: 21/NW/0157). All participants provided informed consent at recruitment to the study for their data to be used for health-related research that was in the public interest.

#### Study population

**Fig 1:**
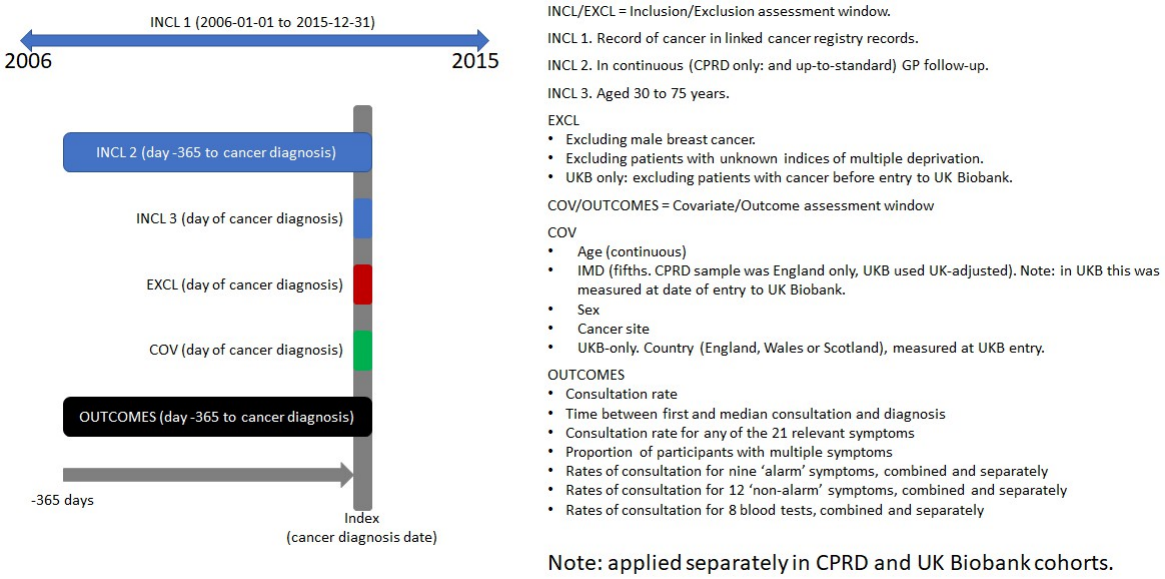
Cohort definition chart for the UK Biobank and CPRD comparison cohorts. A cohort inclusion/exclusion flowchart is given in S1 Fig. **CPRD comparison cohort**. CPRD Gold patients with a cancer diagnosed in 2006–2015, with at least a year of ‘up-to-standard’ primary care registration before diagnosis, and aged 30–75 at diagnosis to match UK Biobank cohort age ranges. **UK Biobank comparison cohort.** UK Biobank participants (with available electronic health records*) with a cancer diagnosed in 2006–2015 and at least a year of continuous GP record before diagnosis (including registrations with different practices if gaps in continuous registration were <91 days). *These patients belonged to the UK Biobank cohort sub-sample with available linked anonymous electronic health records data at the time of analysis.

We derived comparable cohorts within CPRD and UK Biobank for this analysis (Fig 1, S1 Fig). The CPRD cohort was drawn from a random sample of 1,000,000 patients in CPRD, while the UK Biobank cohort was drawn from those UK Biobank participants with available primary care records. Patients were included if they had a cancer diagnosis in 2006–2015 with at least a year of continuous (defined as no gaps in registration of more than 90 days) primary-care follow-up before diagnosis, and were aged 30–75 at diagnosis. Cancer diagnoses were sourced from the national cancer registry, and only the first cancer diagnosis post-2006 was considered. For UK Biobank patients, patients with cancer diagnoses before entry to the UK Biobank study were excluded.

#### Cancer sites

We considered all cancer sites combined (excluding non-melanoma skin cancer), and additionally the ten most common cancer sites in the UK Biobank cohort individually: breast, prostate, colorectal, lung (including mesothelioma), bladder, uterine, kidney, and upper GI cancers (stomach and oesophageal), and non-Hodgkin lymphoma (NHL) and melanoma, with all other sites considered as an ‘other cancer sites’ group. S1 Table gives ICD10 codelists for the sites included in each group.

### Outcomes

Outcomes were identified from coded electronic primary care record data using phenotypes developed for this study; free-text information was not available. We considered rates of primary care consultations, relevant symptoms, and blood tests (Table 1). These outcomes were identified from electronic health records using Read v2 and CTV3 phenotypes developed for this study (S1 Table).

**Table 1:** Details of the different outcome measures considered.

Outcome	Measurement	Justification
Consultations for any reason. May include administrative and other non-clinical events.	Rate (per patient-year)	Capture general primary-care utilisation, expected to increase in year before diagnosis
Symptoms in primary care, as a composite		
Consultations for any of 21 symptoms	Rate	Symptom consultations expected to increase more in year before diagnosis than general consultations
Proportion of patients with multiple distinct symptoms	Proportion	Hints partially at recording, i.e., if it is easy to record symptoms in one system then we might expect more patients with multiple symptoms
‘Alarm’ symptoms in primary care		
Consultations for any of 9 ‘alarm’ symptoms	Rate	Symptoms that are known to be relatively strongly associated with a specific cancer. Typically identified in clinical guidance as indicating urgent assessment for suspected cancer in at least some age groups.
Abdominal lump	Rate	
Breast lump	Rate	
Change in bowel habit	Rate	
Dysphagia	Rate	
Haematuria	Rate	
Haemoptysis	Rate	
Jaundice	Rate	
Post-menopausal bleed	Rate	
Rectal bleeding	Rate	
‘Non-alarm’ symptoms in primary care		
Consultations for any of 12 ‘non-alarm’ symptoms	Rate	Symptoms that are possibly related to a cancer diagnosis, perhaps of specific sites (e.g., abdominal pain and colorectal cancer) or of a range of sites (e.g., fatigue), but which are also common in primary care for other non-cancer reasons.
Abdominal bloating	Rate	
Abdominal pain	Rate	
Constipation	Rate	
Cough	Rate	
Diarrhoea	Rate	
Dyspepsia	Rate	
Dyspnoea (shortness of breath)	Rate	
Fatigue	Rate	
Night sweats	Rate	
Pelvic pain	Rate	
Nausea / vomiting	Rate	
Weight loss	Rate	
Blood tests in primary care		
Any of the below blood tests	Rate	Especially for ‘non-alarm’ symptoms, blood tests in primary care may provide useful information for decision-making by GPs and indicate the possibility of cancer.
Haemoglobin concentration	Rate	
Platelet counts	Rate	
Haematocrit percentage	Rate	
Albumin	Rate	
Ferritin	Rate	
ESR	Rate	
CRP	Rate	
PV	Rate	

One outcome related to the occurrence of primary care consultations (for any reason, without restriction to any specific recorded symptoms): the rate of primary care consultations in the year before cancer diagnosis.

Two outcomes related to primary care consultations for possible cancer symptoms (described subsequently) considered in aggregate (Table 1). First, the rate of primary care consultations for any of the relevant symptoms. Second, the proportion of patients with more than one relevant symptom in the year pre-diagnosis.

Ten outcomes related to ‘alarm’ symptoms for cancer (Table 1), selected based on previous evidence on their relatively high positive predictive value for cancer and clinical guideline recommendations for urgent specialist referral for suspected cancer [7], comprising the rate of consultation for any of nine ‘alarm’ symptoms overall, and for each alarm symptom individually.

Thirteen outcomes related to ‘non-alarm’ symptoms (Table 1), with weaker associations with possible underlying cancer and generally excluded from clinical guidelines on urgent referrals [7], comprising the rate of consultations for any of twelve ‘non-alarm’ symptoms overall and for each non-alarm symptom individually. These represent the non-alarm symptoms deemed of greatest importance [8,25,26].

Nine outcomes related to blood tests that can support the diagnostic process (Table 1), comprising the rate of any of eight blood tests overall, and the rate of each blood test individually.

#### Outcome phenotypes

Each of the 29 symptom and blood test outcomes required a harmonised phenotype appropriate for use across two different coding systems, CTV3 and Read v2. The Read v2 system includes ~88,000 codes, while CTV3 includes ~140,000. Primary care data in CPRD Gold is coded in Read v2, and there are existing Read v2 symptom phenotypes from previous studies which informed the development of phenotypes used in this study [8]. In contrast, the majority of UK Biobank primary care data is recorded in CTV3, and while there are prior CTV3 blood test phenotypes there were no symptom phenotypes [27].

Consequently, we developed harmonised Read v2 and CTV3 symptom phenotypes. Starting with an existing Read v2 phenotypes, we identified candidate CTV3 phenotypes by mapping from Read v2 to CTV3 using published mapping files available from NHS TRUD [28]. These identify codes for the same clinical concept in the two systems, but do not identify all relevant codes and occasionally identified concepts that were not closely related or were generic. Targeted term searches were applied to identify additional potentially-relevant CTV3 codes not identified by the mapping files. The proposed CTV3 phenotypes were reviewed by clinically qualified co-authors (CR and GL) to remove generic codes (e.g., the CTV3 code XM1XP for ‘O/E–pain’), and codes not related to symptoms under investigation. Informal validation included examination of counts and rates of codes in different cuts of the dataset, and assessment of trends over time. The resulting phenotypes included 1,776 codes (1,227 CTV3, 549 Read v2, see S1 Table).

### Statistical analysis

We examined the demographic and cancer case-mix of each cohort, and described the number and proportion of participants with each outcome.

We applied Poisson models for all cancers combined (adjusted for cancer site, age, sex, and fifth of Indices of Multiple Deprivation (IMD)) and each cancer site individually (adjusted for age, sex, and IMD fifth) to examine differences in pre-diagnostic consultation rates and timing of consultations beyond those expected based on simple demographics, both overall and for individual symptoms. Age was parametrised using restricted cubic splines with knots at ages 50, 60 and 70.

The UK Biobank and CPRD cohorts were stored and analysed separately, and the reported results were calculated by an approach analogous to indirect standardisation. For each outcome, a Poisson model was initially fit in CPRD. The coefficients of this model were then applied in the UK Biobank cohort to calculate the outcome rate expected if the associations in UK Biobank were the same as CPRD. A Poisson model was then applied in the UK Biobank cohort to measure the ratio of the observed outcome rate against that expected based on CPRD. Robust standard errors were used to account for possible overdispersion.

The observed differences in rates of observed symptoms and blood tests prompted an exploratory analysis to examine whether differences were seen for all patients in UK Biobank or only for some geographical areas. We compared observed rates in UK Biobank participants who lived in England, Wales, and Scotland at entry to UK Biobank (inferred from the assessment centre) against those expected based on CPRD.

#### Multiple comparisons

Model-based comparisons were only conducted where the UK Biobank and CPRD comparison cohorts each had at least ten participants with the outcome. Given 35 outcomes examined for all cancers and for eleven specific cancer sites, there were 420 potential comparisons of which 270 met minimum sample size criteria above. Analyses for UK Biobank participants’ country added a further 105 comparisons. While we do not report p-values, we provide 95% confidence intervals and these intervals have not been adjusted for multiple testing. Given 525 comparisons we would expect around 25 comparisons where CIs do not cross 1 by chance, even if consultation rates were identical in each cohort. Care should be taken when interpreting specific comparisons, especially those based on relatively small numbers of consultations.

## Results

### Case-mix and demographics of the cohorts

UK Biobank (n = 11,594) and CPRD (n = 22,601) cancer patients in our analysis samples had overall similar age and sex distributions (Table 2), with higher levels of deprivation in CPRD. The cancer case-mix differed substantially. Prostate cancer in particular was overrepresented in UK Biobank (2,190, 19% of cancers) compared with CPRD (3,071, 14%). Breast cancer was also more common in UK Biobank (2,581, 22%) than CPRD (4,369, 19%), while lung cancer was less common (806, 7% vs 2184, 10%).

**Table 2:** Population characteristics and distribution of symptoms, blood tests and primary care consultation patterns in CPRD and UK Biobank.

	UK Biobank cohort	CPRD cohort		
**DEMOGRAPHICS**	**Count**	**(%)**	**Count**	**(%)**
Patients	11,594		22,601	
Male	5,683	(49.0%)	10,453	(46.3%)
Deprivation				
Least deprived	3,594	(31.0%)	5,550	(24.6%)
2nd fifth	2,557	(22.1%)	5,049	(22.3%)
3rd fifth	2,088	(18.0%)	4,795	(21.2%)
4th fifth	1,822	(15.7%)	3,898	(17.2%)
Most deprived	1,533	(13.2%)	3,309	(14.6%)
	**Mean**	**(SD)**	**Mean**	**(SD)**
Age (years)	64.1	(6.9)	59.4	(11.2)
**NUMBER OF CANCERS BY SITE**	**Count**	**(%)**	**Count**	**(%)**
Any cancer	11,594		22,601	
Breast cancer	2,581	(22.3%)	4,379	(19.4%)
Prostate cancer	2,190	(18.9%)	3,071	(13.6%)
Colorectal cancer	1,238	(10.7%)	2,233	(9.9%)
Lung cancer	806	(7.0%)	2,184	(9.7%)
Melanoma	622	(5.4%)	1,011	(4.5%)
NHL	474	(4.1%)	787	(3.5%)
Bladder cancer	380	(3.3%)	709	(3.1%)
Uterine cancer	335	(2.9%)	630	(2.8%)
Upper GI cancer	306	(2.6%)	779	(3.4%)
Kidney cancer	272	(2.3%)	481	(2.1%)
Other cancers	2,390	(20.6%)	6,337	(28.0%)
**CONSULTATIONS**	**Mean**	**(SD)**	**Mean**	**(SD)**
Consultations in the year before diagnosis	14.3	(9.7)	16.2	(11.5)
**POSSIBLE CANCER SYMPTOMS**	**Patients with 1 or more record**	**(%)**	**Patients with 1 or more record**	**(%)**
Any symptom	4,164	(35.9%)	12,187	(53.9%)
Multiple symptoms	1,059	(9.1%)	4,290	(19.0%)
’Alarm’ symptoms	1,891	(16.3%)	5,115	(22.6%)
Abdominal lump	54	(0.5%)	185	(0.8%)
Breast lump	489	(4.2%)	1,888	(8.4%)
Change in bowel habit	111	(1.0%)	362	(1.6%)
Dysphagia	91	(0.8%)	372	(1.6%)
Haematuria	709	(6.1%)	1,054	(4.7%)
Haemoptysis	45	(0.4%)	178	(0.8%)
Jaundice	55	(0.5%)	174	(0.8%)
Post-menopausal bleed	160	(1.4%)	355	(1.6%)
Rectal bleed	242	(2.1%)	770	(3.4%)
’Non-alarm’ symptoms	2,789	(24.1%)	8,991	(39.8%)
Abdominal bloating	101	(0.9%)	346	(1.5%)
Abdominal pain	701	(6.0%)	2,642	(11.7%)
Constipation	208	(1.8%)	731	(3.2%)
Cough	900	(7.8%)	3,497	(15.5%)
Diarrhoea	216	(1.9%)	764	(3.4%)
Dyspepsia	445	(3.8%)	1,082	(4.8%)
Dyspnoea	636	(5.5%)	1,870	(8.3%)
Fatigue	247	(2.1%)	995	(4.4%)
Night sweats	14	(0.1%)	61	(0.3%)
Pelvic pain	9	(0.1%)	24	(0.1%)
Nausea / vomiting	100	(0.9%)	519	(2.3%)
Weight loss	48	(0.4%)	414	(1.8%)
**BLOOD TESTS**	7,469	(64.4%)	14,522	(64.3%)
Full blood count	6,316	(54.5%)	12,914	(57.1%)
Haemoglobin concentration	6,304	(54.4%)	12,903	(57.1%)
Platelet counts	6,294	(54.3%)	12,792	(56.6%)
Haematocrit percentage	6,232	(53.8%)	12,599	(55.7%)
Acute phase response	6,779	(58.5%)	12,940	(57.3%)
Albumin	6,578	(56.7%)	12,663	(56.0%)
Ferritin	1,658	(14.3%)	2,606	(11.5%)
Inflammatory markers	3,154	(27.2%)	7,037	(31.1%)
ESR	2,293	(19.8%)	4,895	(21.7%)
CRP	2,130	(18.4%)	4,707	(20.8%)
PV	247	(2.1%)	781	(3.5%)

### Primary care consultations for any reason

**Fig 2:**
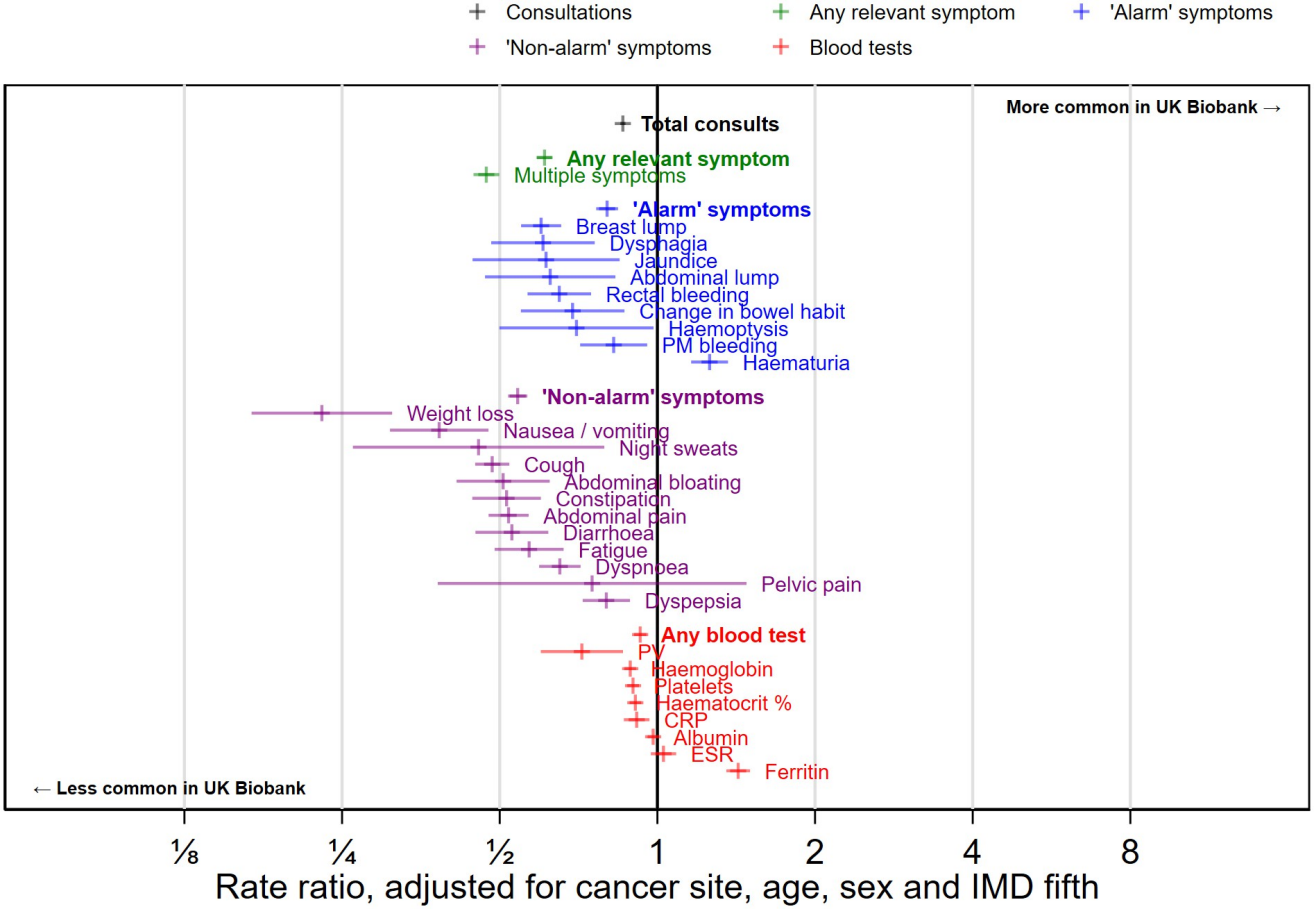
Overall, all-cancers-combined, CPRD-UK Biobank comparisons of consultation, symptom and blood test rates, adjusted for age, sex, deprivation group (IMD fifth) and cancer site.

**Fig 3:**
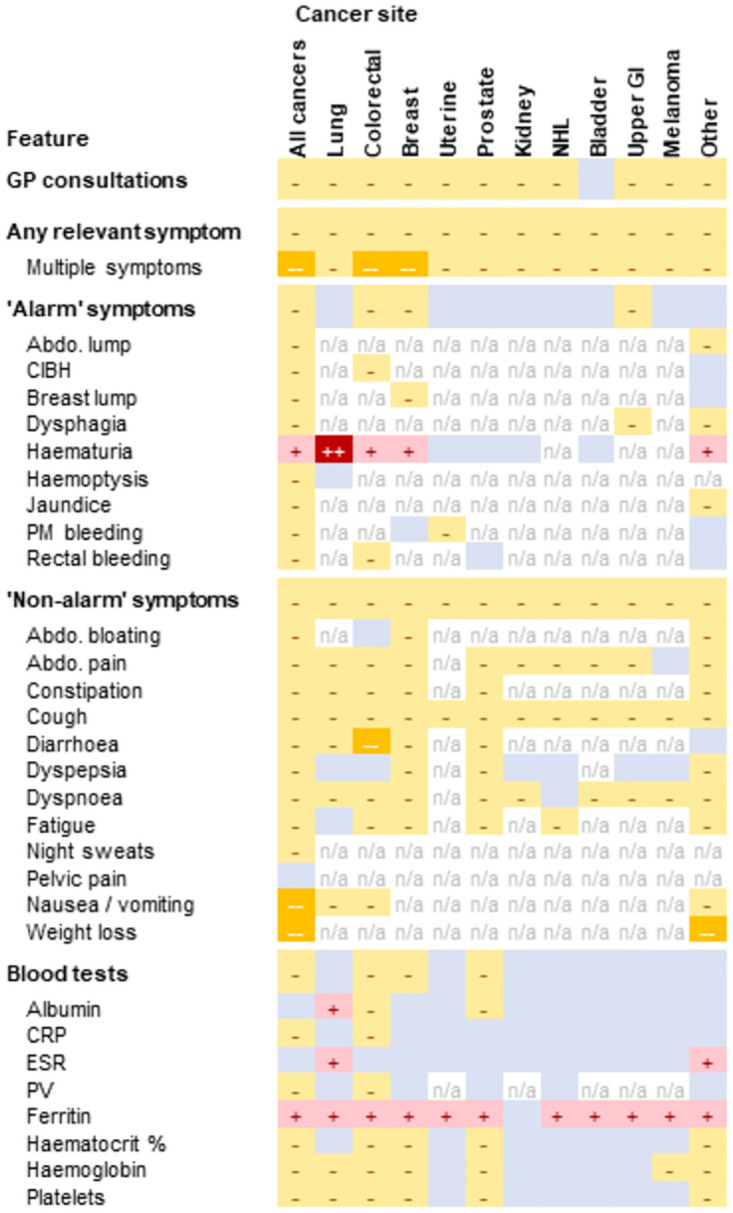
Matrix of comparisons of consultations, symptoms, and blood tests between CPRD and UK Biobank in the year before a cancer diagnosis. Dark yellow—means lower in UKB with upper bound below 0.5. Yellow—means lower in UKB with upper bound below 1. Red + means higher in UKB with lower bound above 1. Dark red ++ means higher in UKB with lower bound above 2. Blue without symbol means the CIs cross 1. n/a means comparisons were not performed due to small sample size.

Nearly all (>99.8%) of cancer patients in UK Biobank and CPRD had at least one primary care consultation in the year pre-diagnosis, although the mean number of consultations was higher in CPRD cancer patients (16.2) compared with UK Biobank cancer patients (14.3, Table 2). After adjusting for cancer site, age, sex, and deprivation, UK Biobank patients had 14% fewer consultations in the year before diagnosis (RR 0.86, 95% CI 0.85 to 0.87, Fig 2). Differences in overall consultation rates in the year before diagnosis were broadly consistent for each individual cancer site considered (Fig 3, all comparisons described in S3 Table).

### Symptomatic consultations and blood tests

Half of CPRD patients (54%, 12,187 of 22,601) had a recorded consultation for at least one of the 21 symptoms we considered in the year before diagnosis (Table 2); one in five (19%, 4,290) had a record of two or more of these symptoms. UK Biobank participants were considerably less likely to have symptom records, with just one third (36%, 4,164 of 11,594) having a record of any of the 21 examined symptoms and one in ten (9%, 1,059) reporting two or more symptoms. These differences persisted after adjustment for age, sex, IMD fifth and cancer site, with a rate ratio of 0.61 (95% CI 0.59 to 0.63) for symptomatic consultations in UK Biobank vs CPRD (Fig 2), and a rate ratio of 0.47 (95% CI 0.45 to 0.50) for the proportion of patients who have consulted for multiple different symptoms in the year pre-diagnosis.

Comparisons for individual symptoms and blood tests are summarised below. Additional results showing comparisons for each individual symptom and blood test and for each of the individual cancer site groups considered are given in S3 Table. Summary figures for each cancer site (showing all symptoms and blood tests) are given in S1 Text. Fig 3 provides an overview of all comparisons.

#### Records of consultations for ‘alarm’ symptoms

23% of CPRD patients and 16% of UK Biobank participants had at least one recorded consultation for an ‘alarm’ symptom recorded in the year before cancer diagnosis (Table 2), with corresponding adjusted (for age, sex, deprivation and cancer site) rate ratio of 0.80 (95% CI 0.76 to 0.84) for UK Biobank vs CPRD (Fig 2). This relatively small difference concealed heterogeneity in the direction of associations. Most symptoms were less common in UK Biobank patients although the size of the difference varied (8/9 alarm symptoms: abdominal lump; breast lump; change in bowel habit; dysphagia; haemoptysis; post-menopausal bleed; jaundice; and rectal bleeding). Haematuria was more common in UK Biobank than CPRD (RR 1.26, 1.15 to 1.36), with more extreme differences for cancer sites for which it is not a relevant symptom (e.g. lung RR 3.82, 95% CI 2.40 to 6.09, S3 Table) than those for which it is (e.g. bladder RR 1.02, 95% CI 0.91 to 1.14, Table 3).

**Table 3:** ‘Alarm’ symptom-cancer pairs in the UK Biobank and CPRD comparison cohorts.

Cancer site	Symptom	UK Biobank comparison cohort			CPRD comparison cohort			Adjusted rate ratio, UK Biobank vs CPRD	
		Patients		(% with symptom)	Patients		(% with symptom)		
		Cancer	Symptom		Cancer	Symptom		RR	(95% CI)
Breast	Breast lump	2,581	461	(17.9%)	4,379	1,771	(40.4%)	0.59	(0.54, 0.65)
Colorectal	Rectal bleeding	1,238	144	(11.6%)	2,233	487	(21.8%)	0.63	(0.53, 0.76)
Bladder	Haematuria	380	213	(56.1%)	709	455	(64.2%)	1.02	(0.91, 1.14)
Uterine	Post-menopausal bleeding	335	117	(34.9%)	630	270	(42.9%)	0.84	(0.71, 1.00)
Kidney	Haematuria	272	53	(19.5%)	481	103	(21.4%)	0.79	(0.60, 1.05)
Upper GI	Dysphagia	306	56	(18.3%)	779	241	(30.9%)	0.58	(0.44, 0.76)

Cancer-specific comparisons included six pairs of ‘alarm’ symptoms and associated cancers (Table 3). For three of these pairs (haematuria and both bladder and kidney cancers, and uterine cancer and post-menopausal bleeding and uterine cancer; and dysphagia and upper GI cancer (i.e. oesophageal and gastric cancer considered jointly), Table 3). For three of these comparisons (bladder cancer and haematuria, kidney cancer and haematuria, and uterine cancer and post-menopausal bleeding), after adjustment there was little evidence of a difference between UK Biobank and CPRD (Table 3). For the other three pairs (breast lump and breast cancer, rectal bleeding and colorectal cancer, and dysphagia and upper GI cancer), there were clear differences between the data sources with these symptoms being less common in UK Biobank patients than in CPRD.

#### Consultations for ‘non-alarm’ symptoms

40% of CPRD and 24% of UK Biobank patients had at least one consultation for a ‘non-alarm’ symptom recorded in the year before cancer diagnosis (Table 2), with the corresponding adjusted (for age, sex, deprivation and cancer site) rate ratio being 0.54 (95% CI 0.52 to 0.56) for UK Biobank vs CPRD (Fig 2).

These patterns applied to all cancer sites (Fig 3), and most individual non-alarm symptoms considered (Figs 2 and 3). Yet, the scale of the differences varied. For example, the rate of consultations for abdominal pain was half as high in UK Biobank as in CPRD (RR 0.52, 95% CI 0.48 to 0.57, Fig 2), while for weight loss it was four times higher in CPRD (RR 0.23, 95% CI 0.17 to 0.31).

#### Blood tests

Sixty-four percent of both CPRD patients and UK Biobank participants had at least one blood test recorded in primary care in the year before cancer diagnosis. Across all cancers combined, and adjusted for age, sex, deprivation and cancer site, the rate ratio for blood tests was 0.93 (95% CI 0.90 to 0.95) in UK Biobank vs CPRD (Fig 2), and most individual blood tests had similar rate ratios to this overall comparison. Ferritin tests were the exception, with higher rates of testing in UK Biobank patients than in CPRD (RR 1.43, 95% CI 1.35 to 1.50).

### Differences in UK Biobank results by country

**Fig 4:**
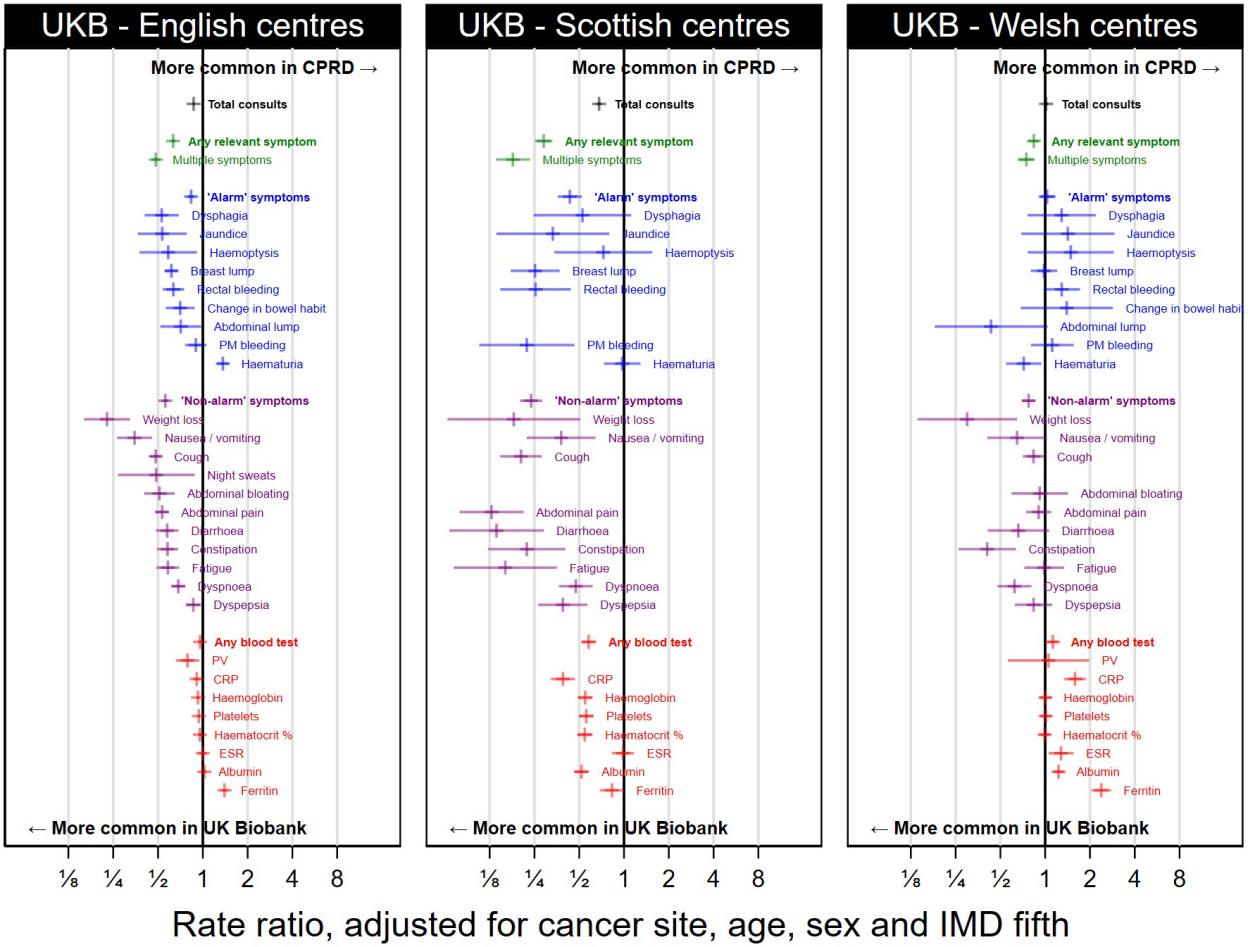
Comparison of rates of symptoms and blood tests of UK Biobank participants in each nation against those in the CPRD cohort. Some comparisons are suppressed due to small numbers.

Exploratory analysis showed that UK Biobank participant differences from CPRD varied for the three UK nations (Fig 4, all country-specific comparisons given in S4 Table), with results for Scotland differing from those for England or Wales. There were larger differences in rates of recorded ‘alarm’ symptoms (RR 0.43, 95% CI 0.36 to 0.52 for Scotland UK Biobank participants vs CPRD, vs a corresponding RR 0.84, 95% CI 0.79 to 0.88 for England UK Biobank participants vs CPRD), and in rates of recorded blood tests (RR 0.58, 95% CI 0.52 to 0.65 for Scotland, vs RR 0.96, 95% CI 0.93 to 0.99 for England).

## Discussion

We created harmonised phenotypes for cancer-relevant symptoms and blood tests in two different primary care coding systems and compared their rates in the year before cancer diagnosis in participants of a cohort study and patients registered in UK general practice.

Nearly all cancer patients in either source had at least one primary care consultation in the year before their cancer diagnosis. Among CPRD cases, half had at least one consultation for one of the studied symptoms in the year before diagnosis, compared with one third in UK Biobank cases. As CPRD is approximately representative of the UK population, this likely means that a slight majority of incident cancer cases in the UK have recorded prodromal symptoms, but that a substantial fraction of patients either do not present to primary care with symptoms or do not have their symptoms recorded.

Differences in recorded alarm symptoms between the CPRD and UKB samples were generally small, with some variability between symptoms. A similar pattern of overall small differences was observed for use of common blood tests. However, differences in non-specific symptoms were far more pronounced.

### Comparisons with existing research

Pre-diagnostic prevalence of symptoms and blood tests in both UK Biobank and CPRD was of comparable magnitude to that reported by the English National Cancer Diagnostic Audit [29], although typically our study found lower prevalence of symptoms which may be due to symptoms being recorded in free-text information but not coded [30]. Other existing symptom studies found prevalence before diagnosis similar to our analysis of CPRD [31,32].

UK Biobank participants are not representative of the UK population [22], but estimates of associations of cardiovascular disease outcomes with known risk factors may be generalisable [20]. In contrast, the risk of adverse health outcomes in multimorbidity may be underestimated in UK Biobank [21]. In the light of existing literature, our results suggest the prevalence and predictive value of health states in selected cohorts should not be considered generalisable to broader populations; and that examination of representativeness needs to be considered in disease-specific contexts.

The findings highlight the risk of relying on single sources of EHRs when estimating prevalence or predictive values of less-specific symptoms (e.g., fatigue, abdominal pain) where it appears recording may be most variable. Despite calls for external validation [22], most studies of decision-support tools based on information on presenting symptoms have thus far been confined to single data sources, such as QResearch [9,10], CPRD [11], or THIN [12].

### Strengths and limitations

The key strengths of this study are: a) the use of high-quality longitudinal EHRs from sources with established previous use in research studies, b) the detailed process followed for the development of *de novo* harmonised phenotypes applicable across both sources of data, and c) the use of approaches akin to indirect standardisation to enable like-for-like comparisons of symptom occurrence in either cohort.

The main limitation is that, by necessity, the analysis could only examine symptoms recorded in the coded data. Free text information was not available for this study, and symptoms may not always be coded in the EHR. Calculating accurate estimates of symptom prevalence may require bespoke data collections such as the English National Cancer Diagnostic Audit [31]. Another challenge was the sample size of patients; cancer-site-specific and country-specific comparisons frequently needed to be suppressed due to the small number of patients with certain symptoms. One UK Biobank primary care data provider in England (Vision) did not provide information on patients who died before the data extract; this will have affected around 600 patients meaning there may be a slight underestimate of both cancer cases and symptoms in UK Biobank, but this could at most explain a small fraction of the differences between UKB and CPRD. Finally, there is possibly a small overlap of UK Biobank participants and CPRD patients, with some patients possibly appearing in both datasets; note however that this would necessarily reduce the size of any possible difference.

Further blood tests of relevance to cancer diagnosis exist that we did not examine, such as calcium and bilirubin. While there would be value in examining recording of further tests, we are reassured that most other blood tests had similar occurrence in both data sources.

Judging external validity of this study is hard due to the selection bias in UK Biobank. Differences between UK Biobank and CPRD will largely be driven by a mix of selection bias in UK Biobank and differences in recording of data due to the different general practice IT systems in use. When comparing other electronic health record datasets based on selected and representative samples, the mix of real differences in health due to selection and apparent differences due to completeness of recording will not be the same; dataset-specific studies will be required.

### Interpretation and implications

Observed differences in prevalence of pre-diagnostic features between UK Biobank and CPRD were principally concentrated in ‘non-alarm’ symptoms of relatively low positive predictive value, where risk of under-recording is greater [30]. However, this does not explain why possible under-recording of non-alarm symptoms should differ between the two sources.

Three hypotheses are worth exploring.

First, differences may truly reflect lower pre-diagnosis symptom prevalence in UK Biobank compared with CPRD cancer cases. This may be because of presentation earlier in the disease process (therefore with lesser symptom burden) or greater proportion of patients detected with asymptomatic disease (e.g., through screening for breast and bowel cancer, or through PSA testing). This may also explain the higher proportion of UK Biobank cancer patients with breast and prostate cancer. Ideally these hypotheses can be examined with information on diagnostic intervals, perhaps especially the time between symptom onset and first consultation with primary care, stage at diagnosis, and diagnostic route [33,34]. However, stage and route to diagnosis information is not yet available in UK Biobank linked cancer registry data.

Differences in comorbidity burden and lifestyle factors may also lead to real differences in rates of symptoms. UK Biobank participants have fewer comorbidities and lower rates of smoking than the general population [22]. Differences in smoking rates may be expected to lead to different rates of respiratory symptoms such as cough or dyspnoea, even among patients with smoking-related cancers. Among patients with colorectal cancer those with pre-existing comorbidities are less likely to present with rectal bleeding or change in bowel habit [35].

Second, there may be a systematic bias in the recording of symptoms in practices represented in UK Biobank and CPRD. Data on UK Biobank cases relates to practices using SystmOne/TPP, whereas data on CPRD cases to Vision. Computer system interface design is known to affect the way EHRs are used [36,37], possibly leading to differences in symptom coding, or how often symptoms are recorded as text rather than coded. For example, interface design could explain the observed excess haematuria codes for patients with lung cancer, which may be misrecorded haemoptysis if the interface suggests codes based on the first four letters. Such a systematic bias is a plausible explanation for the larger discrepancies observed when comparing CPRD vs UK Biobank participants from Scotland. This may also suggest caution when analysing electronic health records for UK Biobank participants from Scotland, as our results suggest they may be more likely to be missing information than records for England or Wales.

Third, our coding harmonisation may have been deficient in imperceptible ways, given differences in the coding systems. However, the exploratory analysis by country showed larger differences between CPRD cases and the Scotland component of UK Biobank cases (both coded Read v2), than between CPRD cases (coded in Read v2) and the England component of UK Biobank cases (chiefly coded in CTV3). This suggests differences are unlikely to be simply or solely due to problems with CTV3 phenotyping.

Overall, the findings offer reassurance but also a cause for concern. It is reassuring that within two UK populations of cancer patients, overall consultation patterns and recording of alarm symptoms and blood tests were broadly similar in the year before the diagnosis, indirectly cross-validating the usefulness of both sources for symptom-based research on cancer diagnosis. However, it is also concerning that on average there was a nearly two-fold difference in the frequency of recording of non-alarm symptoms, because predictive value estimates of such symptoms (for cancer) may be susceptible to bias, and because their accurate estimation is a research priority [38,39]. The potential direction of bias is hard to infer in advance of empirical research, as it interacts with the degree of completeness in the recording of the same symptom among non-cases, but should be addressed by future research [40]. Given the variability in the encoding of symptoms, there is an urgent question about external validity of risk estimates (i.e., whether they can be transported from a given dataset to a population). The results highlight the need for further research of this kind across other sources of primary care electronic health records in the UK and health systems in other countries. Further efforts are needed to develop harmonised phenotypes that can be used across electronic health records datasets, as well as efforts to make use of free-text data from the electronic health record that may contain more complete information on symptoms. The findings support efforts for ensuring equitable participation in contemporary cohort studies collecting genetic, lifestyle and healthcare use data, such as the Our Future Health and All of Us cohorts.

## Supporting information

S1 Fig Cohort inclusion flowchart. (DOCX)

S1 Table Cancer groups and codelists in ICD10. (DOCX)

S2 Table Symptom and blood test codelists in Read v2 and CTV3. (DOCX)

S3 Table All feature comparisons between UK Biobank and CPRD. (DOCX)

S4 Table All feature comparisons between individual countries in UK Biobank and CPRD. These comparisons relate to all cancer sites combined.
(DOCX)

S1 Text Plots of cancer-specific UK Biobank-CPRD comparisons. Fig A. Breast cancer. Fig B. Prostate cancer. Fig C. Colorectal cancer. Fig D. Lung cancer. Fig E. Melanoma. Fig F. NHL. Fig G. Bladder cancer. Fig H. Uterine cancer. Fig I. Kidney cancer. Fig J. Upper GI cancer. Fig K. Other cancers.
(DOCX)

## References

[1] World Health Organisation. Guide to cancer early diagnosis. 2017 [cited 27 Jan 2022]. https://apps.who.int/iris/handle/10665/254500.

[2] Neal RD, Tharmanathan P, France B, Din NU, Cotton S, Fallon-Ferguson J, et al. Is increased time to diagnosis and treatment in symptomatic cancer associated with poorer outcomes? Systematic review. Br J Cancer. 2015;112: S92–S107. doi: 10.1038/bjc.2015.48 25734382 PMC4385982

[3] Mendonca SC, Abel GA, Lyratzopoulos G. Pre-referral GP consultations in patients subsequently diagnosed with rarer cancers: a study of patient-reported data. Br J Gen Pract. 2016;66: e171–e181. doi: 10.3399/bjgp16X683977 26917657 PMC4758496

[4] Swann R, McPhail S, Abel GA, Witt J, Wills L, Hiom S, et al. National Cancer Diagnosis Audits for England 2018 versus 2014: a comparative analysis. Br J Gen Pract. 2023;73: e566–e574. doi: 10.3399/BJGP.2022.0268 37253630 PMC10242853

[5] Sarma EA, Kobrin SC, Thompson MJ. A Proposal to Improve the Early Diagnosis of Symptomatic Cancers in the United States. Cancer Prev Res. 2020;13: 715–720. doi: 10.1158/1940-6207.CAPR-20-0115 32493702

[6] Elliss-Brookes L, McPhail S, Ives A, Greenslade M, Shelton J, Hiom S, et al. Routes to diagnosis for cancer—determining the patient journey using multiple routine data sets. Br J Cancer. 2012;107: 1220–1226. doi: 10.1038/bjc.2012.408 22996611 PMC3494426

[7] National Institute for Health and Care Excellence. Suspected cancer: recognition and referral. NICE Guidel. 2015;NG12. https://www.nice.org.uk/guidance/ng12. 26180880

[8] Moore SF, Price SJ, Chowienczyk S, Bostock J, Hamilton W. The impact of changing risk thresholds on the number of people in England eligible for urgent investigation for possible cancer: an observational cross-sectional study. Br J Cancer. 2021;125: 1593–1597. doi: 10.1038/s41416-021-01541-4 34531548 PMC8445014

[9] Jensen H, Tørring ML, Olesen F, Overgaard J, Vedsted P. Cancer suspicion in general practice, urgent referral and time to diagnosis: a population-based GP survey and registry study. BMC Cancer. 2014;14: 636. doi: 10.1186/1471-2407-14-636 25175155 PMC4164756

[10] Hamilton W, Hajioff S, Graham J, Schmidt-Hansen M. Suspected cancer (part 2—adults): reference tables from updated NICE guidance. Bmj. 2015/06/25. 2015;350: h3044. doi: 10.1136/bmj.h3044 26104465

[11] Shapley M, Mansell G, Jordan JL, Jordan KP. Positive predictive values of ≥5% in primary care for cancer: systematic review. Br J Gen Pract. 2010;60: e366–e377. doi: 10.3399/bjgp10X515412 20849687 PMC2930247

[12] Huggenberger IK, Andersen JS. Predictive value of the official cancer alarm symptoms in general practice—a systematic review. Dan Med J. 2015;62. Available from: http://www.ncbi.nlm.nih.gov/pubmed/26050833 26050833

[13] Hippisley-Cox J, Coupland C. QCancer. 2013 [cited 27 Jul 2023]. https://www.qcancer.org/.

[14] Hippisley-Cox J, Coupland C. Symptoms and risk factors to identify women with suspected cancer in primary care: derivation and validation of an algorithm. Br J Gen Pract. 2013;63: e11. doi: 10.3399/bjgp13X660733 23336450 PMC3529288

[15] Hippisley-Cox J, Coupland C. Symptoms and risk factors to identify men with suspected cancer in primary care: derivation and validation of an algorithm. Br J Gen Pract. 2013;63: e1–e10. doi: 10.3399/bjgp13X660724 23336443 PMC3529287

[16] Jones R, Latinovic R, Charlton J, Gulliford MC. Alarm symptoms in early diagnosis of cancer in primary care: cohort study using General Practice Research Database. BMJ. 2007;334: 1040–1044. doi: 10.1136/bmj.39171.637106.AE 17493982 PMC1871798

[17] Herbert A, Rafiq M, Pham TM, Renzi C, Abel GA, Price S, et al. Predictive values for different cancers and inflammatory bowel disease of 6 common abdominal symptoms among more than 1.9 million primary care patients in the UK: A cohort study. Basu S, editor. PLOS Med. 2021;18: e1003708. doi: 10.1371/journal.pmed.1003708 34339405 PMC8367005

[18] Fritz CDL, Otegbeye EE, Zong X, Demb J, Nickel KB, Olsen MA, et al. Red-flag signs and symptoms for earlier diagnosis of early-onset colorectal cancer. JNCI J Natl Cancer Inst. 2023. doi: 10.1093/jnci/djad068 37138415 PMC10407716

[19] Herrett E, Gallagher AM, Bhaskaran K, Forbes H, Mathur R, van Staa T, et al. Data Resource Profile: Clinical Practice Research Datalink (CPRD). Int J Epidemiol. 2015;44. Available from: https://academic.oup.com/ije/article/44/3/827/632531 26050254 10.1093/ije/dyv098 PMC4521131

[20] Batty GD, Gale CR, Kivimäki M, Deary IJ, Bell S. Comparison of risk factor associations in UK Biobank against representative, general population based studies with conventional response rates: prospective cohort study and individual participant meta-analysis. BMJ. 2020;368. doi: 10.1136/bmj.m131 32051121 PMC7190071

[21] Hanlon P, Jani BD, Nicholl B, Lewsey J, McAllister DA, Mair FS. Associations between multimorbidity and adverse health outcomes in UK Biobank and the SAIL Databank: A comparison of longitudinal cohort studies. PLOS Med. 2022;19: e1003931. doi: 10.1371/journal.pmed.1003931 35255092 PMC8901063

[22] Fry A, Littlejohns TJ, Sudlow C, Doherty N, Adamska L, Sprosen T, et al. Comparison of Sociodemographic and Health-Related Characteristics of UK Biobank Participants With Those of the General Population. Am J Epidemiol. 2017;186: 1026–1034. doi: 10.1093/aje/kwx246 28641372 PMC5860371

[23] NHS Digital. Read Codes. 2022 [cited 12 Jan 2023]. https://digital.nhs.uk/services/terminology-and-classifications/read-codes.

[24] Sudlow C, Gallacher J, Allen N, Beral V, Burton P, Danesh J, et al. UK Biobank: An Open Access Resource for Identifying the Causes of a Wide Range of Complex Diseases of Middle and Old Age. PLOS Med. 2015;12: e1001779. doi: 10.1371/journal.pmed.1001779 25826379 PMC4380465

[25] Nicholson BD, Aveyard P, Price SJ, Hobbs FR, Koshiaris C, Hamilton W. Prioritising primary care patients with unexpected weight loss for cancer investigation: diagnostic accuracy study. BMJ. 2020;370: 2651. doi: 10.1136/bmj.m2651 32816714 PMC7424394

[26] White B, Rafiq M, Gonzalez-Izquierdo A, Hamilton W, Price S, Lyratzopoulos G. Risk of cancer following primary care presentation with fatigue: a population-based cohort study of a quarter of a million patients. Br J Cancer. 2022;126: 1627–1636. doi: 10.1038/s41416-022-01733-6 35181753 PMC9130200

[27] Denaxas S, Shah AD, Mateen BA, Kuan V, Quint JK, Fitzpatrick N, et al. A semi-supervised approach for rapidly creating clinical biomarker phenotypes in the UK Biobank using different primary care EHR and clinical terminology systems. JAMIA Open. 2021;3: 545–556. doi: 10.1093/jamiaopen/ooaa047 33619467 PMC7717266

[28] NHS Digital. NHS TRUD. [cited 13 Oct 2022]. https://isd.digital.nhs.uk/trud/user/guest/group/0/home.

[29] Cranfield B, Koo MM, Abel GA, Swann R, McPhail S, Rubin G, et al. Primary care blood tests before cancer diagnosis: National Cancer Diagnosis Audit data. Br J Gen Pract. 2022; BJGP.2022.0265. doi: 10.3399/BJGP.2022.0265 36253112 PMC9591015

[30] Price SJ, Stapley SA, Shephard E, Barraclough K, Hamilton WT. Is omission of free text records a possible source of data loss and bias in Clinical Practice Research Datalink studies? A case-control study. BMJ Open. 2016;6. doi: 10.1136/BMJOPEN-2016-011664 27178981 PMC4874123

[31] Koo MM, Swann R, McPhail S, Abel GA, Elliss-Brookes L, Rubin GP, et al. Presenting symptoms of cancer and stage at diagnosis: evidence from a cross-sectional, population-based study. Lancet Oncol. 2020;21: 73–79. doi: 10.1016/S1470-2045(19)30595-9 31704137 PMC6941215

[32] Price S, Spencer A, Zhang X, Ball S, Lyratzopoulos G, Mujica-Mota R, et al. Trends in time to cancer diagnosis around the period of changing national guidance on referral of symptomatic patients: A serial cross-sectional study using UK electronic healthcare records from 2006–17. Cancer Epidemiol. 2020;69: 101805. doi: 10.1016/j.canep.2020.101805 32919226 PMC7480981

[33] McPhail S, Elliss-Brookes L, Shelton J, Ives A, Greenslade M, Vernon S, et al. Emergency presentation of cancer and short-term mortality. Br J Cancer. 2013;109: 2027–2034. doi: 10.1038/bjc.2013.569 24045658 PMC3798965

[34] Olesen F, Hansen RP, Vedsted P. Delay in diagnosis: the experience in Denmark. Br J Cancer. 2009;101: S5–S8. doi: 10.1038/sj.bjc.6605383 19956163 PMC2790711

[35] Majano SB, Lyratzopoulos G, Rachet B, de Wit NJ, Renzi C. Do presenting symptoms, use of pre-diagnostic endoscopy and risk of emergency cancer diagnosis vary by comorbidity burden and type in patients with colorectal cancer? Br J Cancer 2021 1264. 2021;126: 652–663. doi: 10.1038/s41416-021-01603-7 34741134 PMC8569047

[36] Walker AJ, MacKenna B, Inglesby P, Tomlinson L, Rentsch CT, Curtis HJ, et al. Clinical coding of long COVID in English primary care: a federated analysis of 58 million patient records in situ using OpenSAFELY. Br J Gen Pract. 2021;71: e806–e814. doi: 10.3399/BJGP.2021.0301 34340970 PMC8340730

[37] Singh H, Sittig DF. A Sociotechnical Framework for Safety-Related Electronic Health Record Research Reporting: The SAFER Reporting Framework. Ann Intern Med. 2020;172: S92–S100. doi: 10.7326/M19-0879 32479184

[38] Nicholson BD, Mant D, Bankhead C. Can safety-netting improve cancer detection in patients with vague symptoms? BMJ. 2016; i5515. doi: 10.1136/bmj.i5515 28291732

[39] Nicholson BD, Lyratzopoulos G. Progress and priorities in reducing the time to cancer diagnosis. Br J Cancer. 2022. doi: 10.1038/s41416-022-02045-5 36344594 PMC9640847

[40] Koo MM, Lyratzopoulos G, Herbert A, Abel GA, Taylor RM, Barber JA, et al. Association of Self-reported Presenting Symptoms With Timeliness of Help-Seeking Among Adolescents and Young Adults With Cancer in the BRIGHTLIGHT Study. JAMA Netw Open. 2020;3: e2015437–e2015437. doi: 10.1001/jamanetworkopen.2020.15437 32880648 PMC7489839

