# Supplementary figures and images for: Phenotypes and rates of cancer-relevant symptoms and tests in the year before cancer diagnosis in UK Biobank and CPRD Gold
Source: PLOS Digit Health. 2023 Dec 15;2(12):e0000383. doi: 10.1371/journal.pdig.0000383 (PMC10723831; doi:10.1371/journal.pdig.0000383)

***S1 Fig. Cohort inclusion flowchart.***


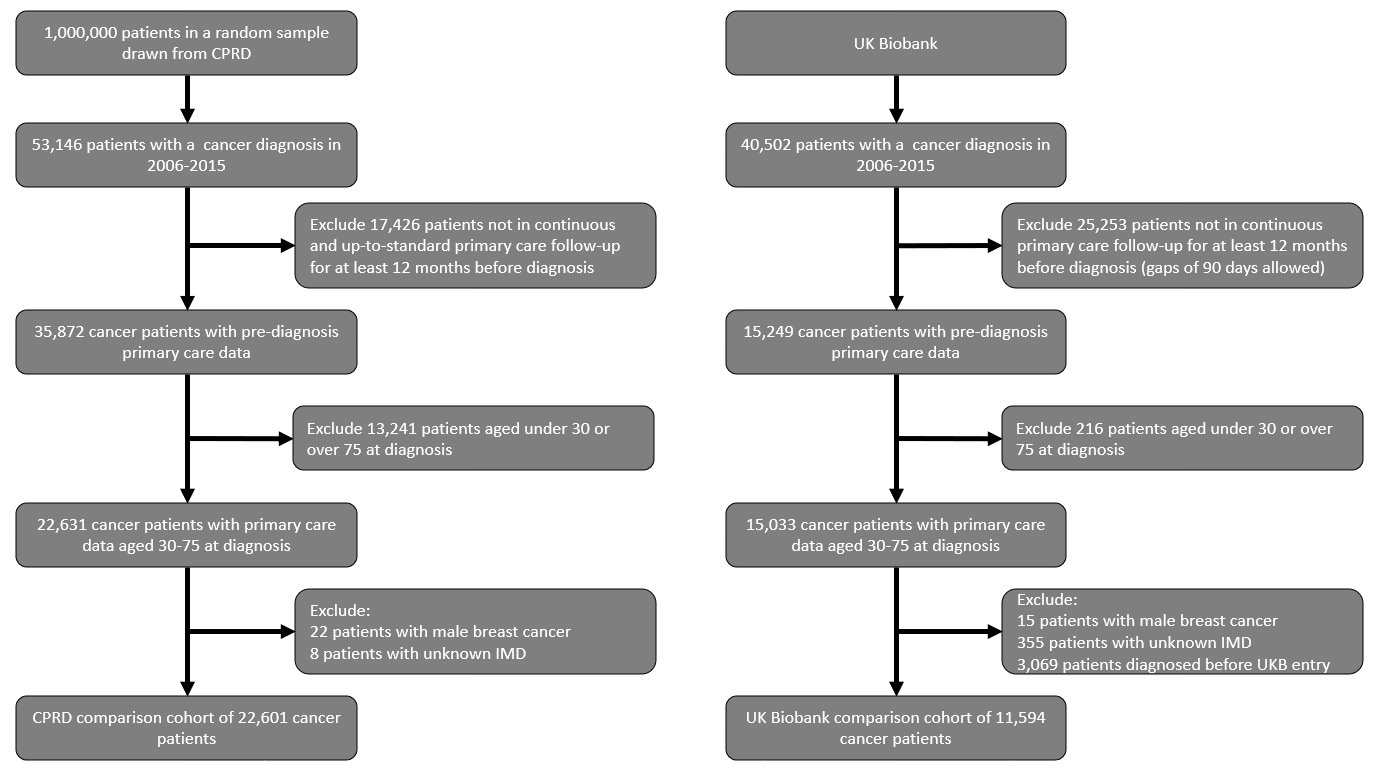

Supplement: S1 Fig — (DOCX) [file pdig.0000383.s001.docx]
