# Supplementary material for: Phenotypes and rates of cancer-relevant symptoms and tests in the year before cancer diagnosis in UK Biobank and CPRD Gold
Source: PLOS Digit Health. 2023 Dec 15;2(12):e0000383. doi: 10.1371/journal.pdig.0000383 (PMC10723831; doi:10.1371/journal.pdig.0000383)
Supplement: S1 Table — (DOCX) [file pdig.0000383.s002.docx]

***S1 Table. Cancer groups and codelists in ICD10.***

| **Analysis group** | **Cancer site** | **ICD10 code** |
| --- | --- | --- |
| Breast | Breast | C50 |
| Breast | Breast (in-situ) | D05 |
| Prostate | Prostate | C61 |
| Colorectal | Colon | C18 |
| Colorectal | Colon | C19 |
| Colorectal | Rectum | C20 |
| Lung | Lung | C33 |
| Lung | Lung | C34 |
| Lung | Mesothelioma | C45 |
| Melanoma | Melanoma | C43 |
| NHL | Non-hodgkin lymphoma | C82 |
| NHL | Non-hodgkin lymphoma | C83 |
| NHL | Non-hodgkin lymphoma | C84 |
| NHL | Non-hodgkin lymphoma | C85 |
| Kidney | Kidney | C64 |
| Upper GI | Oesophagus | C15 |
| Upper GI | Stomach | C16 |
| Bladder | Bladder | C67 |
| Bladder | Bladder (in-situ) | D09 |
| Uterine | Uterus | C54 |
| Uterine | Uterus | C55 |
| Other | Acute myeloid leukaemia | C920 |
| Other | Acute myeloid leukaemia | C924 |
| Other | Acute myeloid leukaemia | C925 |
| Other | Acute myeloid leukaemia | C930 |
| Other | Acute myeloid leukaemia | C940 |
| Other | Acute myeloid leukaemia | C942 |
| Other | Bone sarcoma | C40 |
| Other | Bone sarcoma | C41 |
| Other | Brain | C71 |
| Other | Brain | D330 |
| Other | Brain | D331 |
| Other | Brain | D332 |
| Other | Brain | D430 |
| Other | Brain | D431 |
| Other | Brain | D432 |
| Other | Cervix | C53 |
| Other | Cervix (in-situ) | D06 |
| Other | Chronic lymphocytic leukaemia | C911 |
| Other | Connective and soft tissue sarcoma | C48 |
| Other | Connective and soft tissue sarcoma | C49 |
| Other | Hodgkin lymphoma | C81 |
| Other | Larynx | C32 |
| Other | Liver | C22 |
| Other | Meninges | C70 |
| Other | Meninges | D32 |
| Other | Meninges | D42 |
| Other | Multiple myeloma | C90 |
| Other | Non-specific head and neck | C00 |
| Other | Non-specific head and neck | C14 |
| Other | Non-specific head and neck | C31 |
| Other | Oral cavity | C02 |
| Other | Oral cavity | C03 |
| Other | Oral cavity | C04 |
| Other | Oral cavity | C06 |
| Other | Oropharynx | C01 |
| Other | Oropharynx | C09 |
| Other | Oropharynx | C10 |
| Other | Other CNS and intracranial | C720 |
| Other | Other CNS and intracranial | C721 |
| Other | Other CNS and intracranial | C722 |
| Other | Other CNS and intracranial | C723 |
| Other | Other CNS and intracranial | C724 |
| Other | Other CNS and intracranial | C725 |
| Other | Other CNS and intracranial | C751 |
| Other | Other CNS and intracranial | C752 |
| Other | Other CNS and intracranial | C753 |
| Other | Other CNS and intracranial | D333 |
| Other | Other CNS and intracranial | D334 |
| Other | Other CNS and intracranial | D35 |
| Other | Other CNS and intracranial | D433 |
| Other | Other CNS and intracranial | D434 |
| Other | Other CNS and intracranial | D44 |
| Other | Other and unspecified urinary | C65 |
| Other | Other and unspecified urinary | C66 |
| Other | Other and unspecified urinary | C68 |
| Other | Other haematological | C88 |
| Other | Other haematological | C912 |
| Other | Other haematological | C913 |
| Other | Other haematological | C914 |
| Other | Other haematological | C915 |
| Other | Other haematological | C917 |
| Other | Other haematological | C919 |
| Other | Other haematological | C922 |
| Other | Other haematological | C923 |
| Other | Other haematological | C927 |
| Other | Other haematological | C929 |
| Other | Other haematological | C931 |
| Other | Other haematological | C932 |
| Other | Other haematological | C937 |
| Other | Other haematological | C939 |
| Other | Other haematological | C943 |
| Other | Other haematological | C944 |
| Other | Other haematological | C945 |
| Other | Other haematological | C947 |
| Other | Other haematological | C95 |
| Other | Other haematological | C96 |
| Other | Other head and neck | C05 |
| Other | Other head and neck | C07 |
| Other | Other head and neck | C08 |
| Other | Other head and neck | C11 |
| Other | Other head and neck | C12 |
| Other | Other head and neck | C13 |
| Other | Other leukaemia | C910 |
| Other | Other leukaemia | C921 |
| Other | Other malignant neoplasms | C17 |
| Other | Other malignant neoplasms | C21 |
| Other | Other malignant neoplasms | C23 |
| Other | Other malignant neoplasms | C24 |
| Other | Other malignant neoplasms | C26 |
| Other | Other malignant neoplasms | C30 |
| Other | Other malignant neoplasms | C37 |
| Other | Other malignant neoplasms | C38 |
| Other | Other malignant neoplasms | C39 |
| Other | Other malignant neoplasms | C46 |
| Other | Other malignant neoplasms | C47 |
| Other | Other malignant neoplasms | C52 |
| Other | Other malignant neoplasms | C58 |
| Other | Other malignant neoplasms | C60 |
| Other | Other malignant neoplasms | C63 |
| Other | Other malignant neoplasms | C69 |
| Other | Other malignant neoplasms | C728 |
| Other | Other malignant neoplasms | C729 |
| Other | Other malignant neoplasms | C74 |
| Other | Other malignant neoplasms | C750 |
| Other | Other malignant neoplasms | C754 |
| Other | Other malignant neoplasms | C755 |
| Other | Other malignant neoplasms | C758 |
| Other | Other malignant neoplasms | C759 |
| Other | Other malignant neoplasms | C76 |
| Other | Other malignant neoplasms | C97 |
| Other | Ovary | C56 |
| Other | Ovary | C57 |
| Other | Pancreas | C25 |
| Other | Testis | C62 |
| Other | Thyroid | C73 |
| Other | Unknown primary | C77 |
| Other | Unknown primary | C78 |
| Other | Unknown primary | C79 |
| Other | Unknown primary | C80 |
| Other | Vulva | C51 |
