# Supplementary material for: Phenotypes and rates of cancer-relevant symptoms and tests in the year before cancer diagnosis in UK Biobank and CPRD Gold
Source: PLOS Digit Health. 2023 Dec 15;2(12):e0000383. doi: 10.1371/journal.pdig.0000383 (PMC10723831; doi:10.1371/journal.pdig.0000383)
Supplement: S2 Table — (DOCX) [file pdig.0000383.s003.docx]

***S2 Table. Symptom and blood test codelists in Read v2 and CTV3***

| **Feature** | **Coding system** | **5-digit Read code** | **Description – truncated at 50 characters** |
| --- | --- | --- | --- |
| Abdominal pain | Read v2 | 1829. | retrosternal pain |
| Abdominal pain | Read v2 | 196.. | type of git pain |
| Abdominal pain | Read v2 | 1962. | colicky abdominal pain |
| Abdominal pain | Read v2 | 1963. | non-colicky abdominal pain |
| Abdominal pain | Read v2 | 1968. | abdominal discomfort |
| Abdominal pain | Read v2 | 1969. | abdominal pain |
| Abdominal pain | Read v2 | 19690 | abdominal wall pain |
| Abdominal pain | Read v2 | 196Z. | type of git pain nos |
| Abdominal pain | Read v2 | 197.. | flank pain |
| Abdominal pain | Read v2 | 1971. | central abdominal pain |
| Abdominal pain | Read v2 | 1972. | epigastric pain |
| Abdominal pain | Read v2 | 1973. | left subcostal pain |
| Abdominal pain | Read v2 | 1974. | right subcostal pain |
| Abdominal pain | Read v2 | 1975. | left flank pain |
| Abdominal pain | Read v2 | 1976. | right flank pain |
| Abdominal pain | Read v2 | 1977. | right iliac fossa pain |
| Abdominal pain | Read v2 | 1978. | left iliac fossa pain |
| Abdominal pain | Read v2 | 1979. | suprapubic pain |
| Abdominal pain | Read v2 | 197A. | generalised abdominal pain |
| Abdominal pain | Read v2 | 197B. | upper abdominal pain |
| Abdominal pain | Read v2 | 197C. | lower abdominal pain |
| Abdominal pain | Read v2 | 197D. | right upper quadrant pain |
| Abdominal pain | Read v2 | 197Z. | site of git pain nos |
| Abdominal pain | Read v2 | 1A53. | c/o - loin pain |
| Abdominal pain | Read v2 | 1DC5. | griping pain |
| Abdominal pain | Read v2 | 25C.. | o/e - umbilical pain on palp. |
| Abdominal pain | Read v2 | 25C2. | o/e - abd.pain-r.hypochondrium |
| Abdominal pain | Read v2 | 25C3. | o/e - abd. pain - epigastrium |
| Abdominal pain | Read v2 | 25C4. | o/e - abd.pain-l.hypochondrium |
| Abdominal pain | Read v2 | 25C5. | o/e - abd. pain - r.lumbar |
| Abdominal pain | Read v2 | 25C6. | o/e - abd. pain - umbilical |
| Abdominal pain | Read v2 | 25C7. | o/e - abd. pain - l.lumbar |
| Abdominal pain | Read v2 | 25C8. | o/e - abd. pain - r.iliac |
| Abdominal pain | Read v2 | 25C9. | o/e - abd. pain - hypogastrium |
| Abdominal pain | Read v2 | 25CA. | o/e - abd. pain - l.iliac |
| Abdominal pain | Read v2 | 25CZ. | o/e -abd.pain on palpation nos |
| Abdominal pain | Read v2 | 25D.. | o/e - guarding of abdomen |
| Abdominal pain | Read v2 | 25D2. | o/e - guarding-r.hypochondrium |
| Abdominal pain | Read v2 | 25D3. | o/e - guarding - epigastrium |
| Abdominal pain | Read v2 | 25D4. | o/e - guarding-l.hypochondrium |
| Abdominal pain | Read v2 | 25D6. | o/e - guarding - umbilical |
| Abdominal pain | Read v2 | 25D8. | o/e - guarding - r.iliac |
| Abdominal pain | Read v2 | 25D9. | o/e - guarding - hypogastrium |
| Abdominal pain | Read v2 | 25DA. | o/e - guarding - l.iliac |
| Abdominal pain | Read v2 | 25DZ. | o/e -guarding on palpation nos |
| Abdominal pain | Read v2 | 25E.. | o/e - rebound tenderness |
| Abdominal pain | Read v2 | 25EZ. | o/e - rebound tenderness nos |
| Abdominal pain | Read v2 | 25F.. | o/e - abdominal rigidity |
| Abdominal pain | Read v2 | 25F2. | o/e - acute abdomen |
| Abdominal pain | Read v2 | 2I18. | o/e - tenderness/pain |
| Abdominal pain | Read v2 | 2I181 | tenderness of epigastrium |
| Abdominal pain | Read v2 | E2780 | psychogenic pain unspecified |
| Abdominal pain | Read v2 | R0732 | [d]gas pain (abdominal) |
| Abdominal pain | Read v2 | R090. | [d]abdominal pain |
| Abdominal pain | Read v2 | R0900 | [d]abdominal tenderness |
| Abdominal pain | Read v2 | R0901 | [d]abdominal colic |
| Abdominal pain | Read v2 | R0902 | [d]colic nos |
| Abdominal pain | Read v2 | R0903 | [d]evening colic |
| Abdominal pain | Read v2 | R0904 | [d]abdominal cramps |
| Abdominal pain | Read v2 | R0905 | [d]epigastric pain |
| Abdominal pain | Read v2 | R0906 | [d]umbilical pain |
| Abdominal pain | Read v2 | R0907 | [d]hypochondrial pain |
| Abdominal pain | Read v2 | R0908 | [d]suprapubic pain |
| Abdominal pain | Read v2 | R0909 | [d]pain in right iliac fossa |
| Abdominal pain | Read v2 | R090A | [d]pain in left iliac fossa |
| Abdominal pain | Read v2 | R090C | [d]loin pain |
| Abdominal pain | Read v2 | R090E | [d]recurrent acute abdominal pain |
| Abdominal pain | Read v2 | R090F | [d]acute abdomen |
| Abdominal pain | Read v2 | R090H | [d]upper abdominal pain |
| Abdominal pain | Read v2 | R090J | [d]right upper quadrant pain |
| Abdominal pain | Read v2 | R090K | [d]left upper quadrant pain |
| Abdominal pain | Read v2 | R090L | [d]left lower quadrant pain |
| Abdominal pain | Read v2 | R090M | [d]right lower quadrant pain |
| Abdominal pain | Read v2 | R090N | [d]nonspecific abdominal pain |
| Abdominal pain | Read v2 | R090P | [d]functional abdominal pain syndrome |
| Abdominal pain | Read v2 | R090y | [d]other specified abdominal pain |
| Abdominal pain | Read v2 | R090z | [d]abdominal pain nos |
| Abdominal pain | Read v2 | R094. | [d]abdominal rigidity |
| Abdominal pain | Read v2 | R096. | [d]acute abdomen |
| Abdominal pain | Read v2 | Ryu10 | [x]pain localized to other parts of lower abdomen |
| Abdominal pain | Read v2 | Ryu11 | [x]other and unspecified abdominal pain |
| Abdominal pain | CTV3 | .1829 | retrosternal pain |
| Abdominal pain | CTV3 | .196. | type of git pain |
| Abdominal pain | CTV3 | .1962 | abdominal colic |
| Abdominal pain | CTV3 | .1963 | non-colicky abdominal pain |
| Abdominal pain | CTV3 | .1968 | abdominal discomfort |
| Abdominal pain | CTV3 | .1969 | abdominal pain |
| Abdominal pain | CTV3 | .196Z | type of git pain nos |
| Abdominal pain | CTV3 | .197. | flank pain |
| Abdominal pain | CTV3 | .1971 | central abdominal pain |
| Abdominal pain | CTV3 | .1972 | epigastric pain |
| Abdominal pain | CTV3 | .1973 | left subcostal pain |
| Abdominal pain | CTV3 | .1974 | right subcostal pain |
| Abdominal pain | CTV3 | .1975 | left flank pain |
| Abdominal pain | CTV3 | .1976 | right flank pain |
| Abdominal pain | CTV3 | .1977 | right iliac fossa pain |
| Abdominal pain | CTV3 | .1978 | left iliac fossa pain |
| Abdominal pain | CTV3 | .1979 | suprapubic pain |
| Abdominal pain | CTV3 | .197A | generalised abdominal pain |
| Abdominal pain | CTV3 | .197B | upper abdominal pain |
| Abdominal pain | CTV3 | .197C | lower abdominal pain |
| Abdominal pain | CTV3 | .197D | right upper quadrant pain |
| Abdominal pain | CTV3 | .197Z | site of git pain nos |
| Abdominal pain | CTV3 | .1A53 | loin pain |
| Abdominal pain | CTV3 | .1DC5 | griping pain |
| Abdominal pain | CTV3 | .25C. | o/e - abdominal pain |
| Abdominal pain | CTV3 | .25C1 | o/e - no abd.pain on palpation |
| Abdominal pain | CTV3 | .25C2 | o/e - abd.pain-r.hypochondrium |
| Abdominal pain | CTV3 | .25C3 | o/e - abd. pain - epigastrium |
| Abdominal pain | CTV3 | .25C4 | o/e - abd.pain-l.hypochondrium |
| Abdominal pain | CTV3 | .25C5 | o/e - abd. pain - r.lumbar |
| Abdominal pain | CTV3 | .25C6 | o/e - abd. pain - umbilical |
| Abdominal pain | CTV3 | .25C7 | o/e - abd. pain - l.lumbar |
| Abdominal pain | CTV3 | .25C8 | o/e - abd. pain - r.iliac |
| Abdominal pain | CTV3 | .25C9 | o/e - abd. pain - hypogastrium |
| Abdominal pain | CTV3 | .25CZ | o/e -abd.pain on palpation nos |
| Abdominal pain | CTV3 | .25D. | o/e - guarding on palpation |
| Abdominal pain | CTV3 | .25D2 | o/e - guarding-r.hypochondrium |
| Abdominal pain | CTV3 | .25D3 | o/e - guarding - epigastrium |
| Abdominal pain | CTV3 | .25D4 | o/e - guarding-l.hypochondrium |
| Abdominal pain | CTV3 | .25D6 | o/e - guarding - umbilical |
| Abdominal pain | CTV3 | .25D8 | o/e - guarding - r.iliac |
| Abdominal pain | CTV3 | .25D9 | o/e - guarding - hypogastrium |
| Abdominal pain | CTV3 | .25DA | o/e - guarding - l.iliac |
| Abdominal pain | CTV3 | .25DZ | o/e -guarding on palpation nos |
| Abdominal pain | CTV3 | .25E. | o/e - rebound tenderness |
| Abdominal pain | CTV3 | .25EZ | o/e - rebound tenderness nos |
| Abdominal pain | CTV3 | .25F. | o/e - abdominal rigidity |
| Abdominal pain | CTV3 | .25FZ | o/e - abdominal rigidity nos |
| Abdominal pain | CTV3 | .2I18 | o/e - tenderness/pain |
| Abdominal pain | CTV3 | .R90. | [d]abdominal pain |
| Abdominal pain | CTV3 | .R900 | [d]acute abdomen |
| Abdominal pain | CTV3 | .R901 | [d]abdominal tenderness |
| Abdominal pain | CTV3 | .R902 | [d]abdominal colic |
| Abdominal pain | CTV3 | .R903 | colic [d] |
| Abdominal pain | CTV3 | .R904 | [d]evening colic |
| Abdominal pain | CTV3 | .R905 | [d]abdominal cramps |
| Abdominal pain | CTV3 | .R906 | [d]epigastric pain |
| Abdominal pain | CTV3 | .R907 | [d]umbilical pain |
| Abdominal pain | CTV3 | .R908 | [d]hypochondrial pain |
| Abdominal pain | CTV3 | .R909 | [d]suprapubic pain |
| Abdominal pain | CTV3 | .R90A | [d]pain in right iliac fossa |
| Abdominal pain | CTV3 | .R90B | [d]pain in left iliac fossa |
| Abdominal pain | CTV3 | .R90C | [d]loin pain |
| Abdominal pain | CTV3 | .R90Z | [d]abdominal pain nos |
| Abdominal pain | CTV3 | .R94. | [d]abdominal rigidity |
| Abdominal pain | CTV3 | 1829. | retrosternal pain |
| Abdominal pain | CTV3 | 196.. | type of git pain |
| Abdominal pain | CTV3 | 1962. | abdominal colic |
| Abdominal pain | CTV3 | 1963. | non-colicky abdominal pain |
| Abdominal pain | CTV3 | 1968. | abdominal discomfort |
| Abdominal pain | CTV3 | 1969. | abdominal pain |
| Abdominal pain | CTV3 | 19690 | abdominal wall pain |
| Abdominal pain | CTV3 | 196Z. | type of git pain nos |
| Abdominal pain | CTV3 | 197.. | flank pain |
| Abdominal pain | CTV3 | 1971. | central abdominal pain |
| Abdominal pain | CTV3 | 1972. | epigastric pain |
| Abdominal pain | CTV3 | 1973. | left subcostal pain |
| Abdominal pain | CTV3 | 1974. | right subcostal pain |
| Abdominal pain | CTV3 | 1975. | left flank pain |
| Abdominal pain | CTV3 | 1976. | right flank pain |
| Abdominal pain | CTV3 | 1977. | right iliac fossa pain |
| Abdominal pain | CTV3 | 1978. | left iliac fossa pain |
| Abdominal pain | CTV3 | 1979. | suprapubic pain |
| Abdominal pain | CTV3 | 197A. | generalised abdominal pain |
| Abdominal pain | CTV3 | 197B. | upper abdominal pain |
| Abdominal pain | CTV3 | 197C. | lower abdominal pain |
| Abdominal pain | CTV3 | 197D. | right upper quadrant pain |
| Abdominal pain | CTV3 | 197Z. | site of git pain nos |
| Abdominal pain | CTV3 | 1A53. | c/o - loin pain |
| Abdominal pain | CTV3 | 1DC5. | griping pain |
| Abdominal pain | CTV3 | 25C.. | o/e - abdo. pain on palpation |
| Abdominal pain | CTV3 | 25C1. | o/e - no abd.pain on palpation |
| Abdominal pain | CTV3 | 25C2. | o/e - abd.pain-r.hypochondrium |
| Abdominal pain | CTV3 | 25C3. | o/e - abd. pain - epigastrium |
| Abdominal pain | CTV3 | 25C4. | o/e - abd.pain-l.hypochondrium |
| Abdominal pain | CTV3 | 25C5. | o/e - abd. pain - r.lumbar |
| Abdominal pain | CTV3 | 25C6. | o/e - abd. pain - umbilical |
| Abdominal pain | CTV3 | 25C7. | o/e - abd. pain - l.lumbar |
| Abdominal pain | CTV3 | 25C8. | o/e - abd. pain - r.iliac |
| Abdominal pain | CTV3 | 25C9. | o/e - abd. pain - hypogastrium |
| Abdominal pain | CTV3 | 25CA. | o/e - abd. pain - l.iliac |
| Abdominal pain | CTV3 | 25CZ. | o/e -abd.pain on palpation nos |
| Abdominal pain | CTV3 | 25D.. | o/e - guarding on palpation |
| Abdominal pain | CTV3 | 25D2. | o/e - guarding-r.hypochondrium |
| Abdominal pain | CTV3 | 25D3. | o/e - guarding - epigastrium |
| Abdominal pain | CTV3 | 25D4. | o/e - guarding-l.hypochondrium |
| Abdominal pain | CTV3 | 25D6. | o/e - guarding - umbilical |
| Abdominal pain | CTV3 | 25D8. | o/e - guarding - r.iliac |
| Abdominal pain | CTV3 | 25D9. | o/e - guarding - hypogastrium |
| Abdominal pain | CTV3 | 25DA. | o/e - guarding - l.iliac |
| Abdominal pain | CTV3 | 25DZ. | o/e -guarding on palpation nos |
| Abdominal pain | CTV3 | 25E.. | o/e - rebound tenderness |
| Abdominal pain | CTV3 | 25EZ. | o/e - rebound tenderness nos |
| Abdominal pain | CTV3 | 25F.. | o/e - abdominal rigidity |
| Abdominal pain | CTV3 | 25F2. | o/e - acute abdomen |
| Abdominal pain | CTV3 | 25FZ. | o/e - abdominal rigidity nos |
| Abdominal pain | CTV3 | 2I18. | o/e - tenderness/pain |
| Abdominal pain | CTV3 | 2I181 | tenderness of epigastrium |
| Abdominal pain | CTV3 | E2780 | psychogenic pain unspecified |
| Abdominal pain | CTV3 | R0732 | [d]gas pain (abdominal) |
| Abdominal pain | CTV3 | R090. | [d]abdominal pain |
| Abdominal pain | CTV3 | R0900 | [d]abdominal tenderness |
| Abdominal pain | CTV3 | R0901 | [d]abdominal colic |
| Abdominal pain | CTV3 | R0902 | [d]colic nos |
| Abdominal pain | CTV3 | R0903 | [d]evening colic |
| Abdominal pain | CTV3 | R0904 | [d]abdominal cramps |
| Abdominal pain | CTV3 | R0905 | [d]epigastric pain |
| Abdominal pain | CTV3 | R0906 | [d]umbilical pain |
| Abdominal pain | CTV3 | R0907 | [d]hypochondrial pain |
| Abdominal pain | CTV3 | R0908 | [d]suprapubic pain |
| Abdominal pain | CTV3 | R0909 | [d]pain in right iliac fossa |
| Abdominal pain | CTV3 | R090A | [d]pain in left iliac fossa |
| Abdominal pain | CTV3 | R090C | [d]loin pain |
| Abdominal pain | CTV3 | R090E | [d]recurrent acute abdominal pain |
| Abdominal pain | CTV3 | R090F | [d]acute abdomen |
| Abdominal pain | CTV3 | R090H | [d] upper abdominal pain |
| Abdominal pain | CTV3 | R090J | [d] right upper quadrant pain |
| Abdominal pain | CTV3 | R090K | [d] left upper quadrant pain |
| Abdominal pain | CTV3 | R090L | [d]left lower quadrant pain |
| Abdominal pain | CTV3 | R090M | [d]right lower quadrant pain |
| Abdominal pain | CTV3 | R090N | [d]nonspecific abdominal pain |
| Abdominal pain | CTV3 | R090P | [d]functional abdominal pain syndrome |
| Abdominal pain | CTV3 | R090y | [d]other specified abdominal pain |
| Abdominal pain | CTV3 | R090z | [d]abdominal pain nos |
| Abdominal pain | CTV3 | R094. | [d]abdominal rigidity |
| Abdominal pain | CTV3 | R096. | [d]acute abdomen |
| Abdominal pain | CTV3 | Ryu10 | [x]pain localised to other parts of lower abdomen |
| Abdominal pain | CTV3 | Ryu11 | [x]other and unspecified abdominal pain |
| Abdominal pain | CTV3 | X3062 | chronic nonspecific abdominal pain |
| Abdominal pain | CTV3 | X75rQ | abdominal tenderness |
| Abdominal pain | CTV3 | X75rc | generalised abdominal pain |
| Abdominal pain | CTV3 | X75rd | subcostal pain |
| Abdominal pain | CTV3 | X75re | site of abdominal pain |
| Abdominal pain | CTV3 | X75rf | iliac fossa pain |
| Abdominal pain | CTV3 | X75rg | flank pain |
| Abdominal pain | CTV3 | X75rh | type of git pain - symptom |
| Abdominal pain | CTV3 | X75ri | abdominal pain type |
| Abdominal pain | CTV3 | X75rm | evening colic |
| Abdominal pain | CTV3 | X76dT | abdominal cramps |
| Abdominal pain | CTV3 | XE0r9 | site of git pain |
| Abdominal pain | CTV3 | XE0uZ | flank pain |
| Abdominal pain | CTV3 | XE0up | loin pain |
| Abdominal pain | CTV3 | XE1hU | o/e - abdo. pain on palpation |
| Abdominal pain | CTV3 | XE1hV | o/e - acute abdomen |
| Abdominal pain | CTV3 | XE1iM | o/e - tenderness/pain |
| Abdominal pain | CTV3 | XE1jA | o/e - abdominal pain |
| Abdominal pain | CTV3 | XE1jC | o/e - abd. pain - r.iliac |
| Abdominal pain | CTV3 | XE2Pt | type of git pain |
| Abdominal pain | CTV3 | XE2cT | right iliac fossa pain |
| Abdominal pain | CTV3 | XE2cV | left iliac fossa pain |
| Abdominal pain | CTV3 | XM03B | rebound tenderness |
| Abdominal pain | CTV3 | XM03C | board-like abdominal rigidity |
| Abdominal pain | CTV3 | XM08K | flatulence, eructation and gas pain |
| Abdominal pain | CTV3 | XM08N | gas pain - abdominal |
| Abdominal pain | CTV3 | XM08w | abdominal cramps |
| Abdominal pain | CTV3 | XM08x | umbilical pain |
| Abdominal pain | CTV3 | XM08y | hypochondrial pain |
| Abdominal pain | CTV3 | XM08z | right iliac fossa pain |
| Abdominal pain | CTV3 | XM090 | left iliac fossa pain |
| Abdominal pain | CTV3 | XM092 | loin pain |
| Abdominal pain | CTV3 | XM093 | recurrent acute abdominal pain |
| Abdominal pain | CTV3 | XM094 | other specified abdominal pain |
| Abdominal pain | CTV3 | XM098 | abdominal rigidity |
| Abdominal pain | CTV3 | XM0Cp | c/o - loin pain |
| Abdominal pain | CTV3 | XM0Cq | c/o - lumbar pain |
| Abdominal pain | CTV3 | XM0zS | acute abdomen |
| Abdominal pain | CTV3 | XM0zT | colic [d] |
| Abdominal pain | CTV3 | XM10Y | c/o right iliac fossa pain |
| Abdominal pain | CTV3 | XM10Z | c/o left iliac fossa pain |
| Abdominal pain | CTV3 | XM118 | o/e - abdominal pain |
| Abdominal pain | CTV3 | XM119 | o/e - epigastric pain |
| Abdominal pain | CTV3 | XM11A | o/e - iliac pain - abd. |
| Abdominal pain | CTV3 | XM11C | o/e-umbilical pain-abd. |
| Abdominal pain | CTV3 | XM1B9 | pain localised to other parts of lower abdomen |
| Abdominal pain | CTV3 | XM1NH | o/e - iliac pain on palpation |
| Abdominal pain | CTV3 | XM1NI | o/e - lumbar pain on palpation |
| Abdominal pain | CTV3 | XM1XO | o/e - tenderness |
| Abdominal pain | CTV3 | Xa35t | left sided abdominal pain |
| Abdominal pain | CTV3 | Xa35u | right sided abdominal pain |
| Abdominal pain | CTV3 | Xa7Vz | observation of abdominal rigidity |
| Abdominal pain | CTV3 | Xa7wu | right hypochondrial pain |
| Abdominal pain | CTV3 | Xa7wv | left hypochondrial pain |
| Abdominal pain | CTV3 | Xa7xW | tenderness of epigastrium |
| Abdominal pain | CTV3 | Xa7xf | rebound tenderness of epigastrium |
| Abdominal pain | CTV3 | Xa7xg | rebound tenderness of right hypochondrium |
| Abdominal pain | CTV3 | Xa7xh | rebound tenderness of left hypochondrium |
| Abdominal pain | CTV3 | Xa7xi | rebound tenderness of central region |
| Abdominal pain | CTV3 | Xa7xl | rebound tenderness of hypogastrium |
| Abdominal pain | CTV3 | Xa7xm | rebound tenderness of right iliac fossa |
| Abdominal pain | CTV3 | Xa7xn | rebound tenderness of left iliac fossa |
| Abdominal pain | CTV3 | Xa83q | left upper quadrant pain |
| Abdominal pain | CTV3 | Xa83r | left lower quadrant pain |
| Abdominal pain | CTV3 | Xa83s | right upper quadrant pain |
| Abdominal pain | CTV3 | Xa84T | right lower quadrant pain |
| Abdominal pain | CTV3 | XaA06 | nonspecific abdominal pain |
| Abdominal pain | CTV3 | XaB1I | psychosomatic abdominal pain |
| Abdominal pain | CTV3 | XaBDO | [d] right upper quadrant pain |
| Abdominal pain | CTV3 | XaBDQ | [d] left upper quadrant pain |
| Abdominal pain | CTV3 | XaBDR | [d] upper abdominal pain |
| Abdominal pain | CTV3 | XaBfE | o/e - epigastric pain on palp. |
| Abdominal pain | CTV3 | XaD2x | [d]left lower quadrant pain |
| Abdominal pain | CTV3 | XaD2y | [d]right lower quadrant pain |
| Abdominal pain | CTV3 | XaD2z | [d]nonspecific abdominal pain |
| Abdominal pain | CTV3 | XaEW1 | abdominal pain in early pregnancy |
| Abdominal pain | CTV3 | XaEiJ | unexplained abdominal pain |
| Abdominal pain | CTV3 | XaY2H | [d]functional abdominal pain syndrome |
| Abdominal bloating | Read v2 | 19A.. | abdominal distension symptom |
| Abdominal bloating | Read v2 | 19A2. | abdomen feels bloated |
| Abdominal bloating | Read v2 | 19A3. | abdomen feels distended |
| Abdominal bloating | Read v2 | 19AZ. | abd. distension symptom nos |
| Abdominal bloating | Read v2 | 19B.. | wind symptom |
| Abdominal bloating | Read v2 | 19B2. | excessive flatulence |
| Abdominal bloating | Read v2 | 19B4. | excessive eructation |
| Abdominal bloating | Read v2 | R073. | [d]flatulence, eructation and gas pain |
| Abdominal bloating | Read v2 | R0730 | [d]flatulence |
| Abdominal bloating | Read v2 | R0731 | [d]eructation |
| Abdominal bloating | Read v2 | R0733 | [d]abdominal distension, gaseous |
| Abdominal bloating | Read v2 | R0734 | [d]bloating |
| Abdominal bloating | Read v2 | R073z | [d]flatulence, eructation and gas pain nos |
| Abdominal bloating | CTV3 | .19A. | abdominal distension symptom |
| Abdominal bloating | CTV3 | .19A2 | abdomen feels bloated |
| Abdominal bloating | CTV3 | .19A3 | abdomen feels distended |
| Abdominal bloating | CTV3 | .19AZ | abd. distension symptom nos |
| Abdominal bloating | CTV3 | .19B. | bloating symptom |
| Abdominal bloating | CTV3 | .19B2 | excessive flatulence |
| Abdominal bloating | CTV3 | .19B4 | excessive eructation |
| Abdominal bloating | CTV3 | .R73. | [d]flatulence |
| Abdominal bloating | CTV3 | .R730 | [d]eructation |
| Abdominal bloating | CTV3 | .R732 | [d]abdominal distension, gaseous |
| Abdominal bloating | CTV3 | .R733 | [d]bloating |
| Abdominal bloating | CTV3 | .R73Z | [d]flatulence |
| Abdominal bloating | CTV3 | 19A.. | abdominal distension symptom |
| Abdominal bloating | CTV3 | 19A2. | abdomen feels bloated |
| Abdominal bloating | CTV3 | 19A3. | abdomen feels distended |
| Abdominal bloating | CTV3 | 19AZ. | abd. distension symptom nos |
| Abdominal bloating | CTV3 | 19B.. | bloating symptom |
| Abdominal bloating | CTV3 | 19B2. | excessive flatulence |
| Abdominal bloating | CTV3 | 19B4. | excessive eructation |
| Abdominal bloating | CTV3 | R073. | [d]flatulence, eructation and gas pain |
| Abdominal bloating | CTV3 | R0730 | [d]flatulence |
| Abdominal bloating | CTV3 | R0731 | [d]eructation |
| Abdominal bloating | CTV3 | R0733 | [d]abdominal distension, gaseous |
| Abdominal bloating | CTV3 | R0734 | [d]bloating |
| Abdominal bloating | CTV3 | R073z | [d]flatulence, eructation and gas pain nos |
| Abdominal bloating | CTV3 | Ub1Sa | belching voice |
| Abdominal bloating | CTV3 | X76FC | bloating symptom |
| Abdominal bloating | CTV3 | X76d2 | involuntary passage of flatulence |
| Abdominal bloating | CTV3 | XE0rC | flatulence/wind |
| Abdominal bloating | CTV3 | XM08M | burping |
| Abdominal bloating | CTV3 | XM08O | abdominal distension, gaseous |
| Abdominal bloating | CTV3 | XM1N0 | wind symptom |
| Abdominal bloating | CTV3 | XM1N1 | belching symptom |
| Abdominal bloating | CTV3 | Xa30B | burping in public |
| Abdominal bloating | CTV3 | Xa7V2 | observation of flatulence |
| Abdominal bloating | CTV3 | Xa7Vf | abdominal distension |
| Abdominal bloating | CTV3 | XaBYS | flatulence symptom |
| Abdominal bloating | CTV3 | XaBsc | [d]flatulence nos |
| Breast lump | Read v2 | 1A8.. | breast lump symptom |
| Breast lump | Read v2 | 1A82. | breast lump present |
| Breast lump | Read v2 | 1A83. | breast lump detected by clinician examination |
| Breast lump | Read v2 | 1A84. | breast lump detected by mammogram |
| Breast lump | Read v2 | 1A85. | breast lump detected by partner |
| Breast lump | Read v2 | 1A86. | breast lump detected by self-examination |
| Breast lump | Read v2 | 1A8Z. | breast lump symptom nos |
| Breast lump | Read v2 | 26B7. | lumpy breasts |
| Breast lump | Read v2 | 26E.. | o/e - breast lump palpated |
| Breast lump | Read v2 | 26E2. | o/e-breast lump-nipple/central |
| Breast lump | Read v2 | 26E3. | o/e-breast lump-upper in-quad |
| Breast lump | Read v2 | 26E4. | o/e -breast lump-lower in-quad |
| Breast lump | Read v2 | 26E5. | o/e-breast lump-upper out-quad |
| Breast lump | Read v2 | 26E6. | o/e-breast lump-lower out-quad |
| Breast lump | Read v2 | 26E7. | o/e-breast lump- axillary tail |
| Breast lump | Read v2 | 26EZ. | o/e - breast lump palpated nos |
| Breast lump | Read v2 | 26F.. | o/e - breast lump size |
| Breast lump | Read v2 | 26F1. | o/e - breast lump - pea size |
| Breast lump | Read v2 | 26F2. | o/e - breast lump - plum size |
| Breast lump | Read v2 | 26F3. | o/e-breast lump-tangerine size |
| Breast lump | Read v2 | 26F4. | o/e - breast lump-orange size |
| Breast lump | Read v2 | 26F5. | o/e-breast lump-grapefruit sze |
| Breast lump | Read v2 | 26F6. | o/e - breast lump - melon size |
| Breast lump | Read v2 | 26FZ. | o/e - breast lump size nos |
| Breast lump | Read v2 | 26G.. | o/e - breast lump consistency |
| Breast lump | Read v2 | 26G1. | o/e - breast lump soft |
| Breast lump | Read v2 | 26G2. | o/e - breast lump cystic |
| Breast lump | Read v2 | 26G3. | o/e - breast lump hard |
| Breast lump | Read v2 | 26GZ. | o/e - breast lump consist. nos |
| Breast lump | Read v2 | 26H.. | o/e - breast lump - outline |
| Breast lump | Read v2 | 26H1. | o/e - breast lump smooth |
| Breast lump | Read v2 | 26H2. | o/e - breast lump irregular |
| Breast lump | Read v2 | 26HZ. | o/e-breast lump regularity nos |
| Breast lump | Read v2 | 26I.. | o/e - breast lump tethering |
| Breast lump | Read v2 | 26I1. | o/e - breast lump not tethered |
| Breast lump | Read v2 | 26I2. | o/e -breast lump fixed to skin |
| Breast lump | Read v2 | 26I3. | o/e - breast lump fixed deep |
| Breast lump | Read v2 | 26IZ. | o/e - breast lump tethered nos |
| Breast lump | Read v2 | K3171 | breast mass |
| Breast lump | CTV3 | .1A8. | breast lump symptom |
| Breast lump | CTV3 | .1A82 | breast lump present |
| Breast lump | CTV3 | .1A83 | breast lump detected by clinician examination |
| Breast lump | CTV3 | .1A84 | breast lump detected by mammogram |
| Breast lump | CTV3 | .1A85 | breast lump detected by partner |
| Breast lump | CTV3 | .1A86 | breast lump detected by self-examination |
| Breast lump | CTV3 | .1A8Z | breast lump symptom nos |
| Breast lump | CTV3 | .26E. | o/e - breast lump palpated |
| Breast lump | CTV3 | .26E1 | o/e - no breast lump palpable |
| Breast lump | CTV3 | .26E2 | o/e-breast lump-nipple/central |
| Breast lump | CTV3 | .26E3 | o/e-breast lump-upper in-quad |
| Breast lump | CTV3 | .26E4 | o/e -breast lump-lower in-quad |
| Breast lump | CTV3 | .26E5 | o/e-breast lump-upper out-quad |
| Breast lump | CTV3 | .26E6 | o/e-breast lump-lower out-quad |
| Breast lump | CTV3 | .26E7 | o/e-breast lump- axillary tail |
| Breast lump | CTV3 | .26EZ | o/e - breast lump palpated nos |
| Breast lump | CTV3 | .26F. | o/e - breast lump size |
| Breast lump | CTV3 | .26F1 | o/e - breast lump - pea size |
| Breast lump | CTV3 | .26F2 | o/e - breast lump - plum size |
| Breast lump | CTV3 | .26F3 | o/e-breast lump-tangerine size |
| Breast lump | CTV3 | .26F4 | o/e - breast lump-orange size |
| Breast lump | CTV3 | .26F5 | o/e-breast lump-grapefruit sze |
| Breast lump | CTV3 | .26F6 | o/e - breast lump - melon size |
| Breast lump | CTV3 | .26FZ | o/e - breast lump size nos |
| Breast lump | CTV3 | .26G. | o/e - breast lump consistency |
| Breast lump | CTV3 | .26G1 | o/e - breast lump soft |
| Breast lump | CTV3 | .26G2 | o/e - breast lump cystic |
| Breast lump | CTV3 | .26G3 | o/e - breast lump hard |
| Breast lump | CTV3 | .26GZ | o/e - breast lump consist. nos |
| Breast lump | CTV3 | .26H. | o/e - breast lump regularity |
| Breast lump | CTV3 | .26H1 | o/e - breast lump smooth |
| Breast lump | CTV3 | .26H2 | o/e - breast lump irregular |
| Breast lump | CTV3 | .26HZ | o/e-breast lump regularity nos |
| Breast lump | CTV3 | .26I. | o/e - breast lump tethering |
| Breast lump | CTV3 | .26I1 | o/e - breast lump not tethered |
| Breast lump | CTV3 | .26I2 | o/e -breast lump fixed to skin |
| Breast lump | CTV3 | .26I3 | o/e - breast lump fixed deep |
| Breast lump | CTV3 | .26IZ | o/e - breast lump tethered nos |
| Breast lump | CTV3 | .7F11 | breast lumpectomy |
| Breast lump | CTV3 | .J41. | benign breast lump |
| Breast lump | CTV3 | 1A8.. | breast lump symptom |
| Breast lump | CTV3 | 1A82. | breast lump present |
| Breast lump | CTV3 | 1A83. | breast lump detected by clinician examination |
| Breast lump | CTV3 | 1A84. | breast lump detected by mammogram |
| Breast lump | CTV3 | 1A85. | breast lump detected by partner |
| Breast lump | CTV3 | 1A86. | breast lump detected by self-examination |
| Breast lump | CTV3 | 1A8Z. | breast lump symptom nos |
| Breast lump | CTV3 | 26B7. | lumpy breasts |
| Breast lump | CTV3 | 26E.. | o/e - breast lump palpated |
| Breast lump | CTV3 | 26E1. | o/e - no breast lump palpable |
| Breast lump | CTV3 | 26E2. | o/e-breast lump-nipple/central |
| Breast lump | CTV3 | 26E3. | o/e-breast lump-upper in-quad |
| Breast lump | CTV3 | 26E4. | o/e -breast lump-lower in-quad |
| Breast lump | CTV3 | 26E5. | o/e-breast lump-upper out-quad |
| Breast lump | CTV3 | 26E6. | o/e-breast lump-lower out-quad |
| Breast lump | CTV3 | 26E7. | o/e-breast lump- axillary tail |
| Breast lump | CTV3 | 26EZ. | o/e - breast lump palpated nos |
| Breast lump | CTV3 | 26F.. | o/e - breast lump size |
| Breast lump | CTV3 | 26F1. | o/e - breast lump - pea size |
| Breast lump | CTV3 | 26F2. | o/e - breast lump - plum size |
| Breast lump | CTV3 | 26F3. | o/e-breast lump-tangerine size |
| Breast lump | CTV3 | 26F4. | o/e - breast lump-orange size |
| Breast lump | CTV3 | 26F5. | o/e-breast lump-grapefruit sze |
| Breast lump | CTV3 | 26F6. | o/e - breast lump - melon size |
| Breast lump | CTV3 | 26FZ. | o/e - breast lump size nos |
| Breast lump | CTV3 | 26G.. | o/e - breast lump consistency |
| Breast lump | CTV3 | 26G1. | o/e - breast lump soft |
| Breast lump | CTV3 | 26G2. | o/e - breast lump cystic |
| Breast lump | CTV3 | 26G3. | o/e - breast lump hard |
| Breast lump | CTV3 | 26GZ. | o/e - breast lump consist. nos |
| Breast lump | CTV3 | 26H.. | o/e - breast lump regularity |
| Breast lump | CTV3 | 26H1. | o/e - breast lump smooth |
| Breast lump | CTV3 | 26H2. | o/e - breast lump irregular |
| Breast lump | CTV3 | 26HZ. | o/e-breast lump regularity nos |
| Breast lump | CTV3 | 26I.. | o/e - breast lump tethering |
| Breast lump | CTV3 | 26I1. | o/e - breast lump not tethered |
| Breast lump | CTV3 | 26I2. | o/e -breast lump fixed to skin |
| Breast lump | CTV3 | 26I3. | o/e - breast lump fixed deep |
| Breast lump | CTV3 | 26IZ. | o/e - breast lump tethered nos |
| Breast lump | CTV3 | 71312 | breast lumpectomy |
| Breast lump | CTV3 | K3171 | breast mass |
| Breast lump | CTV3 | XE1he | o/e - breast lump palpated |
| Breast lump | CTV3 | XE2Rd | benign breast lump |
| Breast lump | CTV3 | XM0AY | breast mass |
| Breast lump | CTV3 | XM1YU | o/e - breast lump position |
| Breast lump | CTV3 | Xa4iQ | lumpy breasts |
| Breast lump | CTV3 | XaKis | breast lump detected by clinician examination |
| Breast lump | CTV3 | XaKit | breast lump detected by mammogram |
| Breast lump | CTV3 | XaKiu | breast lump detected by partner |
| Breast lump | CTV3 | XaKiv | breast lump detected by self-examination |
| Change in bowel habit | Read v2 | 19EA. | altered bowel habit |
| Change in bowel habit | Read v2 | 6643. | git symptom changes |
| Change in bowel habit | Read v2 | R078. | [d]change in bowel habit |
| Change in bowel habit | CTV3 | .19EA | change in bowel habit |
| Change in bowel habit | CTV3 | .6643 | git symptom changes |
| Change in bowel habit | CTV3 | 19EA. | change in bowel habit |
| Change in bowel habit | CTV3 | 6643. | git symptom changes |
| Change in bowel habit | CTV3 | R078. | [d]change in bowel habit |
| Dyspepsia | Read v2 | 195.. | indigestion symptoms |
| Dyspepsia | Read v2 | 1954. | indigestion |
| Dyspepsia | Read v2 | 1958. | undiagnosed dyspepsia |
| Dyspepsia | Read v2 | 195Z. | indigestion symptom nos |
| Dyspepsia | Read v2 | 19B3. | excessive belching |
| Dyspepsia | Read v2 | 8Hg5. | discharged from care of dyspepsia specialist nurse |
| Dyspepsia | Read v2 | 8Hl0. | referral to dyspepsia specialist nurse |
| Dyspepsia | Read v2 | 9NNK. | under care of dyspepsia specialist nurse |
| Dyspepsia | Read v2 | E2644 | psychogenic dyspepsia |
| Dyspepsia | Read v2 | J101. | oesophagitis |
| Dyspepsia | Read v2 | J1011 | acid reflux |
| Dyspepsia | Read v2 | J1012 | chemical oesophagitis |
| Dyspepsia | Read v2 | J101y | other specified oesophagitis |
| Dyspepsia | Read v2 | J101z | oesophagitis nos |
| Dyspepsia | Read v2 | J10y4 | acid reflux |
| Dyspepsia | Read v2 | J16y4 | dyspepsia |
| Dyspepsia | Read v2 | J16yA | non-ulcer dyspepsia |
| Dyspepsia | CTV3 | .195. | indigestion symptoms |
| Dyspepsia | CTV3 | .1954 | indigestion |
| Dyspepsia | CTV3 | .1955 | acid reflux |
| Dyspepsia | CTV3 | .1958 | undiagnosed dyspepsia |
| Dyspepsia | CTV3 | .195Z | indigestion symptom nos |
| Dyspepsia | CTV3 | .19B3 | excessive belching |
| Dyspepsia | CTV3 | .8Hg5 | discharged from care of dyspepsia specialist nurse |
| Dyspepsia | CTV3 | .8Hl0 | referral to dyspepsia specialist nurse |
| Dyspepsia | CTV3 | .9NNK | under care of dyspepsia specialist nurse |
| Dyspepsia | CTV3 | .I212 | oesophagitis |
| Dyspepsia | CTV3 | .I264 | dyspepsia |
| Dyspepsia | CTV3 | 195.. | indigestion symptoms |
| Dyspepsia | CTV3 | 1954. | indigestion |
| Dyspepsia | CTV3 | 1955. | acid reflux |
| Dyspepsia | CTV3 | 1958. | undiagnosed dyspepsia |
| Dyspepsia | CTV3 | 195Z. | indigestion symptom nos |
| Dyspepsia | CTV3 | 19B3. | excessive belching |
| Dyspepsia | CTV3 | 8Hg5. | discharged from care of dyspepsia specialist nurse |
| Dyspepsia | CTV3 | 8Hl0. | referral to dyspepsia specialist nurse |
| Dyspepsia | CTV3 | 9NNK. | under care of dyspepsia specialist nurse |
| Dyspepsia | CTV3 | E2644 | psychogenic dyspepsia |
| Dyspepsia | CTV3 | J101. | oesophagitis |
| Dyspepsia | CTV3 | J1011 | acid reflux |
| Dyspepsia | CTV3 | J1012 | chemical oesophagitis |
| Dyspepsia | CTV3 | J101y | other specified oesophagitis |
| Dyspepsia | CTV3 | J101z | oesophagitis nos |
| Dyspepsia | CTV3 | J1020 | ulcerative oesophagitis |
| Dyspepsia | CTV3 | J10y4 | acid reflux |
| Dyspepsia | CTV3 | J16y4 | dyspepsia |
| Dyspepsia | CTV3 | J16yA | non-ulcer dyspepsia |
| Dyspepsia | CTV3 | X3003 | acid reflux |
| Dyspepsia | CTV3 | X3004 | biliary oesophagitis |
| Dyspepsia | CTV3 | X3007 | barrett's oesophagus with oesophagitis |
| Dyspepsia | CTV3 | X3008 | non-reflux oesophagitis |
| Dyspepsia | CTV3 | X301d | non-ulcer dyspepsia |
| Dyspepsia | CTV3 | X76d4 | indigestion nos |
| Dyspepsia | CTV3 | X76d5 | flatulent dyspepsia |
| Dyspepsia | CTV3 | X77H1 | acid reflux status |
| Dyspepsia | CTV3 | X7948 | regurgitated food |
| Dyspepsia | CTV3 | XC0BB | regurgitation |
| Dyspepsia | CTV3 | XE0aL | reflux oesophagitis |
| Dyspepsia | CTV3 | XE0bv | oesophagitis |
| Dyspepsia | CTV3 | XE0cB | dyspepsia |
| Dyspepsia | CTV3 | XM08H | acid reflux |
| Dyspepsia | CTV3 | Xa1q7 | peptic oesophagitis |
| Dyspepsia | CTV3 | Xa7MX | drug-induced dyspepsia |
| Dyspepsia | CTV3 | Xa96h | regurgitation |
| Dyspepsia | CTV3 | XaKbv | discharged from care of dyspepsia specialist nurse |
| Dyspepsia | CTV3 | XaKbw | under care of dyspepsia specialist nurse |
| Dyspepsia | CTV3 | XaLRu | undiagnosed dyspepsia |
| Dyspepsia | CTV3 | XaMDx | referral to dyspepsia specialist nurse |
| Dyspepsia | CTV3 | a24x. | pepcidtwo chewable indigestion tablet |
| Dyspepsia | CTV3 | x02AL | pepcid ac indigestion tablet |
| Dysphagia | Read v2 | 194.. | swallowing symptoms |
| Dysphagia | Read v2 | 1942. | difficulty swallowing solids |
| Dysphagia | Read v2 | 1943. | difficulty swallowing liquids |
| Dysphagia | Read v2 | 1944. | painful swallowing |
| Dysphagia | Read v2 | 194Z. | swallowing symptom nos |
| Dysphagia | Read v2 | 1952. | regurgitates food |
| Dysphagia | Read v2 | 1CB2. | choking sensation |
| Dysphagia | Read v2 | 8E42. | swallowing defect amelioration |
| Dysphagia | Read v2 | 9Oq7. | dysphagia screening completed |
| Dysphagia | Read v2 | D00y0 | sideropenic dysphagia |
| Dysphagia | Read v2 | H470. | pneumonitis due to inhalation of food or vomitus |
| Dysphagia | Read v2 | H4700 | pneumonitis due to inhalation of regurgitated food |
| Dysphagia | Read v2 | H5C.. | choking due to airways obstruction |
| Dysphagia | Read v2 | J100. | cardiospasm - oesophagus |
| Dysphagia | Read v2 | Q311. | massive aspiration syndrome |
| Dysphagia | Read v2 | Q311z | massive aspiration syndrome nos |
| Dysphagia | Read v2 | R04z0 | [d]choking sensation |
| Dysphagia | Read v2 | R072. | [d]dysphagia |
| Dysphagia | Read v2 | R0720 | [d]difficulty in swallowing |
| Dysphagia | Read v2 | R072z | [d]dysphagia nos |
| Dysphagia | Read v2 | SG31. | choked on regurgitated food |
| Dysphagia | Read v2 | TB14. | aspiration of fluid as the cause of abnormal react |
| Dysphagia | Read v2 | TB14z | aspiration of fluid as the cause of abnormal react |
| Dysphagia | Read v2 | TF1.. | inhalation and ingestion of food causing obstructi |
| Dysphagia | Read v2 | TF10. | asphyxia by food |
| Dysphagia | Read v2 | TF102 | asphyxia by regurgitated food |
| Dysphagia | Read v2 | TF11. | choked on food |
| Dysphagia | Read v2 | TF112 | choked on regurgitated food |
| Dysphagia | Read v2 | TF11z | choked on food nos |
| Dysphagia | Read v2 | TF12. | suffocation by food |
| Dysphagia | Read v2 | TF13. | respiratory obstruction due to food lodged in oeso |
| Dysphagia | Read v2 | TF130 | tracheal compression due to food lodged in oesopha |
| Dysphagia | Read v2 | TF131 | interruption of respiration due to food lodged in |
| Dysphagia | Read v2 | TF132 | respiratory obstruction due to food lodged in oeso |
| Dysphagia | Read v2 | TF133 | obstruction of pharynx by food |
| Dysphagia | Read v2 | TF13z | respiratory obstruction due to food lodged in oeso |
| Dysphagia | Read v2 | TF1z. | inhalation and ingestion of food causing respirato |
| Dysphagia | Read v2 | U144. | [x]inhalation and ingestion of food causing obstru |
| Dysphagia | Read v2 | U1441 | [x]inhalation and ingestion of food causing obstru |
| Dysphagia | Read v2 | U144z | [x]inhalation and ingestion of food causing obstru |
| Dysphagia | Read v2 | ZD34. |  |
| Dysphagia | Read v2 | ZD51. |  |
| Dysphagia | Read v2 | ZD53. |  |
| Dysphagia | Read v2 | ZD541 |  |
| Dysphagia | Read v2 | ZV416 | [v]problem with swallowing or mastication |
| Dysphagia | CTV3 | .194. | swallowing symptoms |
| Dysphagia | CTV3 | .1942 | difficulty swallowing solids |
| Dysphagia | CTV3 | .1943 | difficulty swallowing liquids |
| Dysphagia | CTV3 | .1944 | swallowing painful |
| Dysphagia | CTV3 | .194Z | swallowing symptom nos |
| Dysphagia | CTV3 | .1952 | regurgitates food |
| Dysphagia | CTV3 | .8E42 | swallowing defect amelioration |
| Dysphagia | CTV3 | .I211 | achalasia of cardia |
| Dysphagia | CTV3 | .Q4C1 | asphyxia by inhalation of food |
| Dysphagia | CTV3 | .R4Z0 | [d]choking sensation |
| Dysphagia | CTV3 | .R72. | [d]dysphagia |
| Dysphagia | CTV3 | .R720 | [d]difficulty in swallowing |
| Dysphagia | CTV3 | .R72Z | [d]dysphagia nos |
| Dysphagia | CTV3 | 194.. | swallowing symptoms |
| Dysphagia | CTV3 | 1942. | difficulty swallowing solids |
| Dysphagia | CTV3 | 1943. | difficulty swallowing liquids |
| Dysphagia | CTV3 | 1944. | swallowing painful |
| Dysphagia | CTV3 | 194Z. | swallowing symptom nos |
| Dysphagia | CTV3 | 1952. | regurgitates food |
| Dysphagia | CTV3 | 1CB2. | choking sensation |
| Dysphagia | CTV3 | 8E42. | swallowing defect amelioration |
| Dysphagia | CTV3 | 9Oq7. | dysphagia screening completed |
| Dysphagia | CTV3 | D00y0 | sideropenic dysphagia |
| Dysphagia | CTV3 | H470. | pneumonitis due to inhalation of food or vomitus |
| Dysphagia | CTV3 | H4700 | pneumonitis due to inhalation of regurgitated food |
| Dysphagia | CTV3 | H5C.. | choking due to airways obstruction |
| Dysphagia | CTV3 | J100. | achalasia of cardia |
| Dysphagia | CTV3 | Q311. | neonatal massive aspiration syndrome |
| Dysphagia | CTV3 | Q311z | massive aspiration syndrome nos |
| Dysphagia | CTV3 | R04z0 | [d]choking sensation |
| Dysphagia | CTV3 | R072. | [d]dysphagia |
| Dysphagia | CTV3 | R0720 | [d]difficulty in swallowing |
| Dysphagia | CTV3 | R072z | [d]dysphagia nos |
| Dysphagia | CTV3 | SG31. | choked on regurgitated food |
| Dysphagia | CTV3 | TB14. | aspiration of fluid as the cause of abnormal react |
| Dysphagia | CTV3 | TB14z | aspiration of fluid as the cause of abnormal react |
| Dysphagia | CTV3 | TF1.. | inhalation and ingestion of food causing obstructi |
| Dysphagia | CTV3 | TF10. | asphyxia by inhalation of food |
| Dysphagia | CTV3 | TF102 | asphyxia by regurgitated food in air passages |
| Dysphagia | CTV3 | TF11. | choked on food |
| Dysphagia | CTV3 | TF112 | choked on regurgitated food |
| Dysphagia | CTV3 | TF11z | choked on food nos |
| Dysphagia | CTV3 | TF12. | choked on food |
| Dysphagia | CTV3 | TF13. | respiratory obstruction due to food lodged in oeso |
| Dysphagia | CTV3 | TF130 | tracheal compression due to food lodged in oesopha |
| Dysphagia | CTV3 | TF131 | interruption of respiration due to food lodged in |
| Dysphagia | CTV3 | TF132 | respiratory obstruction due to food lodged in oeso |
| Dysphagia | CTV3 | TF133 | obstruction of pharynx by food |
| Dysphagia | CTV3 | TF13z | respiratory obstruction due to food lodged in oeso |
| Dysphagia | CTV3 | TF1z. | inhalation and ingestion of food causing respirato |
| Dysphagia | CTV3 | U144. | [x]inhalation and ingestion of food causing obstru |
| Dysphagia | CTV3 | U1441 | [x]inhalation and ingestion of food causing obstru |
| Dysphagia | CTV3 | U144z | [x]inhalation and ingestion of food causing obstru |
| Dysphagia | CTV3 | XE0r7 | swallowing symptoms |
| Dysphagia | CTV3 | XE0r8 | regurgitates food |
| Dysphagia | CTV3 | XE1eo | massive aspiration syndrome nos |
| Dysphagia | CTV3 | XM08J | dysphagia |
| Dysphagia | CTV3 | XaW9A | dysphagia screening completed |
| Dysphagia | CTV3 | ZD34. |  |
| Dysphagia | CTV3 | ZD51. |  |
| Dysphagia | CTV3 | ZD53. |  |
| Dysphagia | CTV3 | ZD541 |  |
| Dysphagia | CTV3 | ZV416 | [v]problem with swallowing or mastication |
| Dyspnoea | Read v2 | 173.. | breathlessness |
| Dyspnoea | Read v2 | 1732. | breathless - moderate exertion |
| Dyspnoea | Read v2 | 1733. | breathless - mild exertion |
| Dyspnoea | Read v2 | 1734. | breathless - at rest |
| Dyspnoea | Read v2 | 1735. | orthopnoea symptom |
| Dyspnoea | Read v2 | 1736. | paroxysmal nocturnal dyspnoea |
| Dyspnoea | Read v2 | 1738. | difficulty breathing |
| Dyspnoea | Read v2 | 1739. | shortness of breath |
| Dyspnoea | Read v2 | 173C. | dyspnoea on exertion |
| Dyspnoea | Read v2 | 173D. | nocturnal dyspnoea |
| Dyspnoea | Read v2 | 173F. | short of breath dressing/undressing |
| Dyspnoea | Read v2 | 173G. | breathless - strenuous exertion |
| Dyspnoea | Read v2 | 173H. | mrc breathlessness scale: grade 1 |
| Dyspnoea | Read v2 | 173I. | mrc breathlessness scale: grade 2 |
| Dyspnoea | Read v2 | 173J. | mrc breathlessness scale: grade 3 |
| Dyspnoea | Read v2 | 173K. | mrc breathlessness scale: grade 4 |
| Dyspnoea | Read v2 | 173L. | mrc breathlessness scale: grade 5 |
| Dyspnoea | Read v2 | 173N. | borg breathlessness score: 0.5 very, very slight ( |
| Dyspnoea | Read v2 | 173P. | borg breathlessness score: 1 very slight |
| Dyspnoea | Read v2 | 173Q. | borg breathlessness score: 2 slight |
| Dyspnoea | Read v2 | 173R. | borg breathlessness score: 3 moderate |
| Dyspnoea | Read v2 | 173S. | borg breathlessness score: 4 somewhat severe |
| Dyspnoea | Read v2 | 173T. | borg breathlessness score: 5 severe |
| Dyspnoea | Read v2 | 173V. | borg breathlessness score: 6 severe (+) |
| Dyspnoea | Read v2 | 173W. | borg breathlessness score: 7 very severe |
| Dyspnoea | Read v2 | 173X. | borg breathlessness score: 8 very severe (+) |
| Dyspnoea | Read v2 | 173Y. | borg breathlessness score: 9 very, very severe (al |
| Dyspnoea | Read v2 | 173Z. | breathlessness nos |
| Dyspnoea | Read v2 | 173a. | borg breathlessness score: 10 maximal |
| Dyspnoea | Read v2 | 173g. | breathlessness causing difficulty eating |
| Dyspnoea | Read v2 | 2322. | o/e - dyspnoea |
| Dyspnoea | Read v2 | 2323. | o/e - orthopnoea |
| Dyspnoea | Read v2 | 2324. | o/e - respiratory distress |
| Dyspnoea | Read v2 | 388H. | cardiovascular limitations and symptoms profile sh |
| Dyspnoea | Read v2 | 38Gb. | dyspnoea, airflow obstruction, smoking status, exa |
| Dyspnoea | Read v2 | R0602 | [d]orthopnoea |
| Dyspnoea | Read v2 | R0605 | [d]cheyne-stokes respiration |
| Dyspnoea | Read v2 | R0606 | [d]respiratory distress |
| Dyspnoea | Read v2 | R0607 | [d]respiratory insufficiency |
| Dyspnoea | Read v2 | R0608 | [d]shortness of breath |
| Dyspnoea | Read v2 | R060A | [d]dyspnoea |
| Dyspnoea | Read v2 | R060D | [d]breathlessness |
| Dyspnoea | Read v2 | ZR3Q. |  |
| Dyspnoea | CTV3 | .173. | breathlessness |
| Dyspnoea | CTV3 | .1732 | breathless - moderate exertion |
| Dyspnoea | CTV3 | .1733 | breathless - mild exertion |
| Dyspnoea | CTV3 | .1734 | breathless - at rest |
| Dyspnoea | CTV3 | .1735 | breathless - lying flat |
| Dyspnoea | CTV3 | .1736 | paroxysmal nocturnal dyspnoea |
| Dyspnoea | CTV3 | .1738 | difficulty breathing |
| Dyspnoea | CTV3 | .1739 | dyspnoea |
| Dyspnoea | CTV3 | .173C | dyspnoea on exertion |
| Dyspnoea | CTV3 | .173D | nocturnal dyspnoea |
| Dyspnoea | CTV3 | .173F | short of breath dressing/undressing |
| Dyspnoea | CTV3 | .173G | breathless - strenuous exertion |
| Dyspnoea | CTV3 | .173H | mrc breathlessness scale: grade 1 |
| Dyspnoea | CTV3 | .173I | mrc breathlessness scale: grade 2 |
| Dyspnoea | CTV3 | .173J | mrc breathlessness scale: grade 3 |
| Dyspnoea | CTV3 | .173K | mrc breathlessness scale: grade 4 |
| Dyspnoea | CTV3 | .173L | mrc breathlessness scale: grade 5 |
| Dyspnoea | CTV3 | .173N | borg breathlessness score: 0.5 very, very slight ( |
| Dyspnoea | CTV3 | .173P | borg breathlessness score: 1 very slight |
| Dyspnoea | CTV3 | .173Q | borg breathlessness score: 2 slight |
| Dyspnoea | CTV3 | .173R | borg breathlessness score: 3 moderate |
| Dyspnoea | CTV3 | .173S | borg breathlessness score: 4 somewhat severe |
| Dyspnoea | CTV3 | .173T | borg breathlessness score: 5 severe |
| Dyspnoea | CTV3 | .173V | borg breathlessness score: 6 severe (+) |
| Dyspnoea | CTV3 | .173W | borg breathlessness score: 7 very severe |
| Dyspnoea | CTV3 | .173X | borg breathlessness score: 8 very severe (+) |
| Dyspnoea | CTV3 | .173Y | borg breathlessness score: 9 very, very severe (al |
| Dyspnoea | CTV3 | .173Z | breathlessness nos |
| Dyspnoea | CTV3 | .173a | borg breathlessness score: 10 maximal |
| Dyspnoea | CTV3 | .2322 | o/e - dyspnoea |
| Dyspnoea | CTV3 | .2323 | o/e - orthopnoea |
| Dyspnoea | CTV3 | .2324 | o/e - respiratory distress |
| Dyspnoea | CTV3 | .388H | cardiovascular limitations and symptoms profile sh |
| Dyspnoea | CTV3 | .R600 | [d]dyspnoea |
| Dyspnoea | CTV3 | .R602 | [d]orthopnoea |
| Dyspnoea | CTV3 | .R605 | [d]cheyne-stokes respiration |
| Dyspnoea | CTV3 | .R606 | [d]respiratory distress |
| Dyspnoea | CTV3 | .R607 | respiratory insufficiency [d] |
| Dyspnoea | CTV3 | .R608 | [d]shortness of breath |
| Dyspnoea | CTV3 | 173.. | breathlessness |
| Dyspnoea | CTV3 | 1732. | breathless - moderate exertion |
| Dyspnoea | CTV3 | 1733. | breathless - mild exertion |
| Dyspnoea | CTV3 | 1734. | breathless - at rest |
| Dyspnoea | CTV3 | 1735. | breathless - lying flat |
| Dyspnoea | CTV3 | 1736. | paroxysmal nocturnal dyspnoea |
| Dyspnoea | CTV3 | 1738. | difficulty breathing |
| Dyspnoea | CTV3 | 1739. | dyspnoea |
| Dyspnoea | CTV3 | 173C. | dyspnoea on exertion |
| Dyspnoea | CTV3 | 173D. | nocturnal dyspnoea |
| Dyspnoea | CTV3 | 173F. | short of breath dressing/undressing |
| Dyspnoea | CTV3 | 173G. | breathless - strenuous exertion |
| Dyspnoea | CTV3 | 173H. | mrc breathlessness scale: grade 1 |
| Dyspnoea | CTV3 | 173I. | mrc breathlessness scale: grade 2 |
| Dyspnoea | CTV3 | 173J. | mrc breathlessness scale: grade 3 |
| Dyspnoea | CTV3 | 173K. | mrc breathlessness scale: grade 4 |
| Dyspnoea | CTV3 | 173L. | mrc breathlessness scale: grade 5 |
| Dyspnoea | CTV3 | 173N. | borg breathlessness score: 0.5 very, very slight ( |
| Dyspnoea | CTV3 | 173P. | borg breathlessness score: 1 very slight |
| Dyspnoea | CTV3 | 173Q. | borg breathlessness score: 2 slight |
| Dyspnoea | CTV3 | 173R. | borg breathlessness score: 3 moderate |
| Dyspnoea | CTV3 | 173S. | borg breathlessness score: 4 somewhat severe |
| Dyspnoea | CTV3 | 173T. | borg breathlessness score: 5 severe |
| Dyspnoea | CTV3 | 173V. | borg breathlessness score: 6 severe (+) |
| Dyspnoea | CTV3 | 173W. | borg breathlessness score: 7 very severe |
| Dyspnoea | CTV3 | 173X. | borg breathlessness score: 8 very severe (+) |
| Dyspnoea | CTV3 | 173Y. | borg breathlessness score: 9 very, very severe (al |
| Dyspnoea | CTV3 | 173Z. | breathlessness nos |
| Dyspnoea | CTV3 | 173a. | borg breathlessness score: 10 maximal |
| Dyspnoea | CTV3 | 173g. | breathlessness causing difficulty eating |
| Dyspnoea | CTV3 | 2322. | o/e - dyspnoea |
| Dyspnoea | CTV3 | 2323. | o/e - orthopnoea |
| Dyspnoea | CTV3 | 2324. | o/e - respiratory distress |
| Dyspnoea | CTV3 | 388H. | cardiovascular limitations and symptoms profile sh |
| Dyspnoea | CTV3 | 38Gb. | dyspnoea, airflow obstruction, smoking status, exa |
| Dyspnoea | CTV3 | H59.. | respiratory insufficiency |
| Dyspnoea | CTV3 | R0602 | [d]orthopnoea |
| Dyspnoea | CTV3 | R0605 | [d]cheyne-stokes respiration |
| Dyspnoea | CTV3 | R0606 | [d]respiratory distress |
| Dyspnoea | CTV3 | R0607 | [d]respiratory insufficiency |
| Dyspnoea | CTV3 | R0608 | [d]shortness of breath |
| Dyspnoea | CTV3 | R060A | [d]dyspnoea |
| Dyspnoea | CTV3 | R060D | [d]breathlessness |
| Dyspnoea | CTV3 | X76Gy | nocturnal dyspnoea |
| Dyspnoea | CTV3 | X76Gz | dyspnoea on exertion |
| Dyspnoea | CTV3 | X77QT | breathlessness rating |
| Dyspnoea | CTV3 | XE0qq | dyspnoea |
| Dyspnoea | CTV3 | XE0qr | orthopnoea |
| Dyspnoea | CTV3 | XM07z | respiratory distress |
| Dyspnoea | CTV3 | XM09V | respiratory insufficiency |
| Dyspnoea | CTV3 | Xa6Xt | respiratory insufficiency [d] |
| Dyspnoea | CTV3 | Xa6a8 | increasing breathlessness |
| Dyspnoea | CTV3 | XaBMU | [d]breathlessness |
| Dyspnoea | CTV3 | XaINB | cardiovascular limitations and symptoms profile sh |
| Dyspnoea | CTV3 | XaIQ2 | short of breath dressing/undressing |
| Dyspnoea | CTV3 | XaIQ3 | breathless - strenuous exertion |
| Dyspnoea | CTV3 | XaIUi | mrc breathlessness scale: grade 1 |
| Dyspnoea | CTV3 | XaIUl | mrc breathlessness scale: grade 2 |
| Dyspnoea | CTV3 | XaIUm | mrc breathlessness scale: grade 3 |
| Dyspnoea | CTV3 | XaIUn | mrc breathlessness scale: grade 4 |
| Dyspnoea | CTV3 | XaIUo | mrc breathlessness scale: grade 5 |
| Dyspnoea | CTV3 | XaIvo | borg breathlessness score: 0.5 very, very slight ( |
| Dyspnoea | CTV3 | XaIvs | borg breathlessness score: 1 very slight |
| Dyspnoea | CTV3 | XaIvt | borg breathlessness score: 2 slight |
| Dyspnoea | CTV3 | XaIvu | borg breathlessness score: 3 moderate |
| Dyspnoea | CTV3 | XaIvv | borg breathlessness score: 4 somewhat severe |
| Dyspnoea | CTV3 | XaIvy | borg breathlessness score: 5 severe |
| Dyspnoea | CTV3 | XaIw0 | borg breathlessness score: 6 severe (+) |
| Dyspnoea | CTV3 | XaIw5 | borg breathlessness score: 7 very severe |
| Dyspnoea | CTV3 | XaIw6 | borg breathlessness score: 8 very severe (+) |
| Dyspnoea | CTV3 | XaIw7 | borg breathlessness score: 9 very, very severe (al |
| Dyspnoea | CTV3 | XaIw8 | borg breathlessness score: 10 maximal |
| Dyspnoea | CTV3 | XaX8r | body mass, airflow obstruction, dyspnoea and exerc |
| Dyspnoea | CTV3 | XaXfI | chronic respiratory disease questionnaire dyspnoea |
| Dyspnoea | CTV3 | XaZZr | dyspnoea, airflow obstruction, smoking status, exa |
| Dyspnoea | CTV3 | XaagZ | breathlessness causing difficulty eating |
| Dyspnoea | CTV3 | ZR3Q. |  |
| Fatigue | Read v2 | 168.. | c/o "muzzy head" |
| Fatigue | Read v2 | 1682. | fatigue |
| Fatigue | Read v2 | 1683. | tired all the time |
| Fatigue | Read v2 | 1684. | c/o - overwork |
| Fatigue | Read v2 | 1688. | exhaustion |
| Fatigue | Read v2 | 168Z. | tiredness symptom nos |
| Fatigue | Read v2 | 1B3.. | motor symptoms |
| Fatigue | Read v2 | 1B32. | weakness present |
| Fatigue | Read v2 | 2832. | o/e - paresis |
| Fatigue | Read v2 | 8HkW. | referral to chronic fatigue syndrome specialist te |
| Fatigue | Read v2 | 8HlL. | referral for chronic fatigue syndrome activity man |
| Fatigue | Read v2 | 8Q1.. | activity management for chronic fatigue syndrome |
| Fatigue | Read v2 | E205. | nervous exhaustion |
| Fatigue | Read v2 | Eu460 | [x]neurasthenia |
| Fatigue | Read v2 | F286. | chronic fatigue syndrome |
| Fatigue | Read v2 | F2860 | mild chronic fatigue syndrome |
| Fatigue | Read v2 | F2861 | moderate chronic fatigue syndrome |
| Fatigue | Read v2 | F2862 | severe chronic fatigue syndrome |
| Fatigue | Read v2 | R007. | [d]malaise and fatigue |
| Fatigue | Read v2 | R0070 | [d]malaise |
| Fatigue | Read v2 | R0071 | [d]fatigue |
| Fatigue | Read v2 | R0072 | [d]asthenia nos |
| Fatigue | Read v2 | R0073 | [d]lethargy |
| Fatigue | Read v2 | R0074 | [d]post viral debility |
| Fatigue | Read v2 | R0075 | [d]tiredness |
| Fatigue | Read v2 | R007z | [d]overwork |
| Fatigue | Read v2 | R204. | [d]senile exhaustion |
| Fatigue | Read v2 | R2y3. | [d] self neglect |
| Fatigue | CTV3 | .168. | fatigue - symptom |
| Fatigue | CTV3 | .1682 | fatigue |
| Fatigue | CTV3 | .1683 | tired all the time |
| Fatigue | CTV3 | .1688 | exhaustion |
| Fatigue | CTV3 | .168Z | tiredness symptom nos |
| Fatigue | CTV3 | .1B3. | weakness symptoms |
| Fatigue | CTV3 | .1B32 | weakness present |
| Fatigue | CTV3 | .2832 | o/e - weakness |
| Fatigue | CTV3 | .8Q1. | activity management for chronic fatigue syndrome |
| Fatigue | CTV3 | .A871 | postviral infection debility |
| Fatigue | CTV3 | .F38. | chronic fatigue syndrome |
| Fatigue | CTV3 | .P9C5 | transient heat fatigue |
| Fatigue | CTV3 | .P9EZ | overexertion exhaustion |
| Fatigue | CTV3 | .R07. | [d]malaise and fatigue |
| Fatigue | CTV3 | .R070 | [d]malaise |
| Fatigue | CTV3 | .R071 | [d]fatigue |
| Fatigue | CTV3 | .R072 | asthenia [d] |
| Fatigue | CTV3 | .R073 | [d]lethargy |
| Fatigue | CTV3 | .R074 | [d]postviral (asthenic) syndrome |
| Fatigue | CTV3 | .R07Z | [d]malaise and fatigue nos |
| Fatigue | CTV3 | .RH4. | [d]senile exhaustion |
| Fatigue | CTV3 | .RJ3. | [d]debility, unspecified |
| Fatigue | CTV3 | 168.. | c/o "muzzy head" |
| Fatigue | CTV3 | 1682. | fatigue |
| Fatigue | CTV3 | 1683. | tired all the time |
| Fatigue | CTV3 | 1684. | c/o - overwork |
| Fatigue | CTV3 | 1688. | exhaustion |
| Fatigue | CTV3 | 168Z. | tiredness symptom nos |
| Fatigue | CTV3 | 1B3.. | weakness symptoms |
| Fatigue | CTV3 | 1B32. | weakness present |
| Fatigue | CTV3 | 2832. | o/e - weakness |
| Fatigue | CTV3 | 8HkW. | referral to chronic fatigue syndrome specialist te |
| Fatigue | CTV3 | 8HlL. | referral for chronic fatigue syndrome activity man |
| Fatigue | CTV3 | 8Q1.. | activity management for chronic fatigue syndrome |
| Fatigue | CTV3 | E205. | tired all the time |
| Fatigue | CTV3 | Eu460 | neurasthenia |
| Fatigue | CTV3 | F286. | chronic fatigue syndrome |
| Fatigue | CTV3 | F2860 | mild chronic fatigue syndrome |
| Fatigue | CTV3 | F2861 | moderate chronic fatigue syndrome |
| Fatigue | CTV3 | F2862 | severe chronic fatigue syndrome |
| Fatigue | CTV3 | R007. | [d]malaise and fatigue |
| Fatigue | CTV3 | R0070 | [d]malaise |
| Fatigue | CTV3 | R0071 | [d]fatigue |
| Fatigue | CTV3 | R0072 | [d]asthenia nos |
| Fatigue | CTV3 | R0073 | [d]lethargy |
| Fatigue | CTV3 | R0074 | [d]postviral (asthenic) syndrome |
| Fatigue | CTV3 | R0075 | [d]tiredness |
| Fatigue | CTV3 | R007z | [d]malaise and fatigue nos |
| Fatigue | CTV3 | R204. | [d]senile exhaustion |
| Fatigue | CTV3 | R2y3. | [d] self neglect |
| Fatigue | CTV3 | SN26. | transient heat fatigue |
| Fatigue | CTV3 | X75s8 | chronic fatigue syndrome |
| Fatigue | CTV3 | X76Ac | easily tired |
| Fatigue | CTV3 | X76Ad | tired on least exertion |
| Fatigue | CTV3 | X76Ae | exhaustion |
| Fatigue | CTV3 | X76Am | weakness - general |
| Fatigue | CTV3 | X76p0 | rapid fatigue of gait |
| Fatigue | CTV3 | X76qY | muscle fatigue |
| Fatigue | CTV3 | X77QS | leg fatigue rating |
| Fatigue | CTV3 | XE0UW | postviral infection debility |
| Fatigue | CTV3 | XE0qj | tiredness symptom |
| Fatigue | CTV3 | XE0qk | tired all the time |
| Fatigue | CTV3 | XE0ql | malaise/lethargy |
| Fatigue | CTV3 | XE0uN | fatigue - symptom |
| Fatigue | CTV3 | XE1YD | neurasthenia |
| Fatigue | CTV3 | XE1t7 | overexertion exhaustion |
| Fatigue | CTV3 | XE2y5 | [d]debility, unspecified |
| Fatigue | CTV3 | XM03n | muscle weakness |
| Fatigue | CTV3 | XM06l | malaise and fatigue |
| Fatigue | CTV3 | XM06m | malaise |
| Fatigue | CTV3 | XM06o | lethargy |
| Fatigue | CTV3 | XM06p | chronic fatigue syndrome |
| Fatigue | CTV3 | XM0Ce | c/o - debility - malaise |
| Fatigue | CTV3 | XM0Cg | c/o - postviral syndrome |
| Fatigue | CTV3 | XM0D3 | fatigue - symptom |
| Fatigue | CTV3 | XM0D4 | c/o "muzzy head" |
| Fatigue | CTV3 | XM0D5 | c/o - "tired all the time" |
| Fatigue | CTV3 | XM0yx | asthenia [d] |
| Fatigue | CTV3 | XM1AV | weakness |
| Fatigue | CTV3 | Xa01F | chronic fatigue syndrome |
| Fatigue | CTV3 | XaPeC | activity management for chronic fatigue syndrome |
| Fatigue | CTV3 | XaPom | mild chronic fatigue syndrome |
| Fatigue | CTV3 | XaPon | moderate chronic fatigue syndrome |
| Fatigue | CTV3 | XaPoo | severe chronic fatigue syndrome |
| Fatigue | CTV3 | XaR7C | referral to chronic fatigue syndrome specialist te |
| Fatigue | CTV3 | XaRAz | referral for chronic fatigue syndrome activity man |
| Fatigue | CTV3 | XabDw | symptom assessment scale - fatigue score |
| Haematuria | Read v2 | 14D5. | h/o: haematuria |
| Haematuria | Read v2 | 1A45. | haematuria - symptom |
| Haematuria | Read v2 | 4625. | urine: red - blood |
| Haematuria | Read v2 | 4695. | urine blood test = + |
| Haematuria | Read v2 | 4696. | urine blood test = ++ |
| Haematuria | Read v2 | 4697. | urine blood test = +++ |
| Haematuria | Read v2 | 46G2. | rbcs- red blood cells in urine |
| Haematuria | Read v2 | 8T10. | referral to haematuria clinic |
| Haematuria | Read v2 | K0321 | recurrent benign haematuria syndrome |
| Haematuria | Read v2 | K0A2. | recurrent and persistent haematuria |
| Haematuria | Read v2 | K0A26 | recurrent and persistent haematuria, dense deposit |
| Haematuria | Read v2 | K197. | haematuria |
| Haematuria | Read v2 | K1970 | painless haematuria |
| Haematuria | Read v2 | K1971 | painful haematuria |
| Haematuria | Read v2 | K1973 | frank haematuria |
| Haematuria | Read v2 | K1974 | clot haematuria |
| Haematuria | CTV3 | .14D5 | h/o: haematuria |
| Haematuria | CTV3 | .1A45 | haematuria - symptom |
| Haematuria | CTV3 | .4625 | urine: red - blood |
| Haematuria | CTV3 | .4695 | urine blood test = + |
| Haematuria | CTV3 | .4696 | urine blood test = ++ |
| Haematuria | CTV3 | .4697 | urine blood test = +++ |
| Haematuria | CTV3 | .46G2 | rbcs- red blood cells in urine |
| Haematuria | CTV3 | .J2A1 | frank haematuria |
| Haematuria | CTV3 | .J2A2 | painless haematuria |
| Haematuria | CTV3 | 14D5. | h/o: haematuria |
| Haematuria | CTV3 | 1A45. | haematuria - symptom |
| Haematuria | CTV3 | 4625. | urine: red - blood |
| Haematuria | CTV3 | 4695. | urine blood test = + |
| Haematuria | CTV3 | 4696. | urine blood test = ++ |
| Haematuria | CTV3 | 4697. | urine blood test = +++ |
| Haematuria | CTV3 | 46G2. | rbcs- red blood cells in urine |
| Haematuria | CTV3 | 8T10. | referral to haematuria clinic |
| Haematuria | CTV3 | K0321 | recurrent benign haematuria syndrome |
| Haematuria | CTV3 | K0A2. | recurrent and persistent haematuria |
| Haematuria | CTV3 | K0A26 | recurrent and persistent haematuria, dense deposit |
| Haematuria | CTV3 | K197. | haematuria |
| Haematuria | CTV3 | K1970 | painless haematuria |
| Haematuria | CTV3 | K1971 | painful haematuria |
| Haematuria | CTV3 | K1973 | frank haematuria |
| Haematuria | CTV3 | K1974 | clot haematuria |
| Haematuria | CTV3 | X30Pw | traumatic haematuria |
| Haematuria | CTV3 | X30Px | loin pain - haematuria syndrome |
| Haematuria | CTV3 | X30Pz | upper urinary tract haematuria |
| Haematuria | CTV3 | XE0e5 | haematuria |
| Haematuria | CTV3 | XE0rU | blood in urine - haematuria |
| Haematuria | CTV3 | XE0un | haematuria - symptom |
| Haematuria | CTV3 | XE2sK | urine microscopy:rbc's present |
| Haematuria | CTV3 | Xa1cp | rbcs- red blood cells in urine |
| Haematuria | CTV3 | Xa1uI | persistent frank haematuria |
| Haematuria | CTV3 | Xa1uJ | recurrent frank haematuria |
| Haematuria | CTV3 | Xa1uM | persistent haematuria |
| Haematuria | CTV3 | Xa1uN | recurrent haematuria |
| Haematuria | CTV3 | Xaafu | referral to haematuria clinic |
| Haemoptysis | Read v2 | 172.. | blood in sputum - haemoptysis |
| Haemoptysis | Read v2 | R063. | [d]haemoptysis |
| Haemoptysis | Read v2 | R0630 | [d]cough with haemorrhage |
| Haemoptysis | Read v2 | R0631 | [d]pulmonary haemorrhage nos |
| Haemoptysis | Read v2 | R063z | [d]haemoptysis nos |
| Haemoptysis | CTV3 | .172. | blood in sputum - haemoptysis |
| Haemoptysis | CTV3 | .R63. | [d]haemoptysis |
| Haemoptysis | CTV3 | .R630 | [d]cough with haemorrhage |
| Haemoptysis | CTV3 | .R63Z | [d]haemoptysis nos |
| Haemoptysis | CTV3 | 172.. | blood in sputum - haemoptysis |
| Haemoptysis | CTV3 | 4E24. | blood in sputum - haemoptysis |
| Haemoptysis | CTV3 | R063. | [d]haemoptysis |
| Haemoptysis | CTV3 | R0630 | [d]cough with haemorrhage |
| Haemoptysis | CTV3 | R0631 | [d]pulmonary haemorrhage nos |
| Haemoptysis | CTV3 | R063z | [d]haemoptysis nos |
| Haemoptysis | CTV3 | XE0qp | blood in sputum - haemoptysis |
| Jaundice | Read v2 | 1675. | yellow/jaundiced colour |
| Jaundice | Read v2 | 2274. | o/e - jaundiced colour |
| Jaundice | Read v2 | 4223. | o/e: blood looks deep yellow |
| Jaundice | Read v2 | D100. | acholuric familial jaundice |
| Jaundice | Read v2 | J66y6 | obstructive jaundice nos |
| Jaundice | Read v2 | R024. | [d]jaundice (not of newborn) |
| Jaundice | Read v2 | R0240 | [d]cholaemia nos |
| Jaundice | Read v2 | R0241 | [d]icterus nos |
| Jaundice | Read v2 | R024z | [d]jaundice (not of newborn) nos |
| Jaundice | CTV3 | .1675 | yellow/jaundiced colour |
| Jaundice | CTV3 | .2274 | o/e - jaundiced colour |
| Jaundice | CTV3 | .4223 | o/e: blood looks deep yellow |
| Jaundice | CTV3 | .D131 | hereditary spherocytosis |
| Jaundice | CTV3 | .R24. | [d]jaundice (not of newborn) |
| Jaundice | CTV3 | .R24Z | [d]jaundice (not of newborn) nos |
| Jaundice | CTV3 | 1675. | yellow/jaundiced colour |
| Jaundice | CTV3 | 2274. | o/e - jaundiced colour |
| Jaundice | CTV3 | 4223. | o/e: blood looks deep yellow |
| Jaundice | CTV3 | D100. | hereditary spherocytosis |
| Jaundice | CTV3 | J66y6 | obstructive jaundice nos |
| Jaundice | CTV3 | R024. | [d]jaundice (not of newborn) |
| Jaundice | CTV3 | R0240 | [d]cholaemia nos |
| Jaundice | CTV3 | R0241 | [d]icterus nos |
| Jaundice | CTV3 | R024z | [d]jaundice (not of newborn) nos |
| Jaundice | CTV3 | X769K | yellow - symptom |
| Jaundice | CTV3 | X769z | jaundice |
| Jaundice | CTV3 | XC08w | jaundice |
| Jaundice | CTV3 | XE0qi | yellow or jaundiced colour |
| Night sweats | Read v2 | 1662. | night sweats |
| Night sweats | Read v2 | R0084 | [d]night sweats |
| Night sweats | CTV3 | .1662 | night sweats |
| Night sweats | CTV3 | 1662. | night sweats |
| Night sweats | CTV3 | R0084 | night sweats |
| Night sweats | CTV3 | X76A5 | night sweats |
| Post-menopausal bleeding | Read v2 | K5A1. | postmenopausal bleeding |
| Post-menopausal bleeding | CTV3 | .J772 | postmenopausal bleeding |
| Post-menopausal bleeding | CTV3 | K5A1. | postmenopausal bleeding |
| Rectal bleeding | Read v2 | 196B. | painful rectal bleeding |
| Rectal bleeding | Read v2 | 196C. | painless rectal bleeding |
| Rectal bleeding | Read v2 | 19E6. | blood in faeces |
| Rectal bleeding | Read v2 | 19ED. | blood on toilet paper |
| Rectal bleeding | Read v2 | 19EG. | blood on pants |
| Rectal bleeding | Read v2 | 4762. | faeces: fresh blood present |
| Rectal bleeding | Read v2 | 4793. | faecal occult blood: trace |
| Rectal bleeding | Read v2 | 4794. | faecal occult blood: positive |
| Rectal bleeding | Read v2 | 8HTE0 | referral to rectal bleeding clinic |
| Rectal bleeding | Read v2 | G8480 | bleeding haemorrhoids nos |
| Rectal bleeding | Read v2 | J573. | haemorrhage of rectum and anus |
| Rectal bleeding | Read v2 | J5730 | rectal haemorrhage |
| Rectal bleeding | Read v2 | J5731 | anal haemorrhage |
| Rectal bleeding | Read v2 | J573z | haemorrhage of rectum and anus nos |
| Rectal bleeding | Read v2 | J68.. | gastrointestinal haemorrhage |
| Rectal bleeding | Read v2 | J681. | blood in stool |
| Rectal bleeding | Read v2 | J68z. | gastrointestinal haemorrhage unspecified |
| Rectal bleeding | Read v2 | J68z1 | intestinal haemorrhage nos |
| Rectal bleeding | Read v2 | J68zz | gastrointestinal tract haemorrhage nos |
| Rectal bleeding | Read v2 | Q4142 | perinatal rectal haemorrhage |
| Rectal bleeding | CTV3 | .196B | painful rectal bleeding |
| Rectal bleeding | CTV3 | .196C | painless rectal bleeding |
| Rectal bleeding | CTV3 | .19E6 | blood in faeces |
| Rectal bleeding | CTV3 | .19ED | blood on toilet paper |
| Rectal bleeding | CTV3 | .19EG | blood on pants |
| Rectal bleeding | CTV3 | .4762 | faeces: fresh blood present |
| Rectal bleeding | CTV3 | .4793 | faecal occult blood: trace |
| Rectal bleeding | CTV3 | .4794 | faecal occult blood: positive |
| Rectal bleeding | CTV3 | .I78. | gastrointestinal haemorrhage |
| Rectal bleeding | CTV3 | .I782 | melaena |
| Rectal bleeding | CTV3 | .I78Z | gastrointestinal tract haemorrhage nos |
| Rectal bleeding | CTV3 | 196B. | painful rectal bleeding |
| Rectal bleeding | CTV3 | 196C. | painless rectal bleeding |
| Rectal bleeding | CTV3 | 19E6. | blood in faeces |
| Rectal bleeding | CTV3 | 19ED. | blood on toilet paper |
| Rectal bleeding | CTV3 | 19EG. | blood on pants |
| Rectal bleeding | CTV3 | 4762. | faeces: fresh blood present |
| Rectal bleeding | CTV3 | 4793. | faecal occult blood: trace |
| Rectal bleeding | CTV3 | 4794. | faecal occult blood: positive |
| Rectal bleeding | CTV3 | 8HTE0 | referral to rectal bleeding clinic |
| Rectal bleeding | CTV3 | G8480 | bleeding haemorrhoids nos |
| Rectal bleeding | CTV3 | J573. | haemorrhage of rectum and anus |
| Rectal bleeding | CTV3 | J5730 | rectal haemorrhage |
| Rectal bleeding | CTV3 | J5731 | anal haemorrhage |
| Rectal bleeding | CTV3 | J573z | haemorrhage of rectum and anus nos |
| Rectal bleeding | CTV3 | J68.. | gastrointestinal haemorrhage |
| Rectal bleeding | CTV3 | J68z. | gastrointestinal haemorrhage unspecified |
| Rectal bleeding | CTV3 | J68z1 | intestinal haemorrhage nos |
| Rectal bleeding | CTV3 | J68zz | gastrointestinal tract haemorrhage nos |
| Rectal bleeding | CTV3 | Q4142 | perinatal rectal haemorrhage |
| Rectal bleeding | CTV3 | X30Bj | bleeding per rectum |
| Rectal bleeding | CTV3 | X76fI | blood in faeces symptom |
| Rectal bleeding | CTV3 | XE0b0 | haemorrhage of rectum and anus |
| Rectal bleeding | CTV3 | XE0bI | melaena |
| Rectal bleeding | CTV3 | XE0bJ | gastrointestinal haemorrhage unspecified |
| Rectal bleeding | CTV3 | XE0rJ | blood in stool |
| Rectal bleeding | CTV3 | XE26B | faeces: fresh blood present |
| Rectal bleeding | CTV3 | XM1VX | blood in stool |
| Rectal bleeding | CTV3 | XaFt2 | blood on toilet paper |
| Rectal bleeding | CTV3 | XaIMX | blood on pants |
| Rectal bleeding | CTV3 | XaJuu | painful rectal bleeding |
| Rectal bleeding | CTV3 | XaJuv | painless rectal bleeding |
| Rectal bleeding | CTV3 | XaYVt | referral to rectal bleeding clinic |
| Weight loss | Read v2 | 1623. | weight decreasing |
| Weight loss | Read v2 | 1625. | abnormal weight loss |
| Weight loss | Read v2 | 1627. | unintentional weight loss |
| Weight loss | Read v2 | 1D1A. | complaining of weight loss |
| Weight loss | Read v2 | 2224. | o/e - cachexic |
| Weight loss | Read v2 | 2287. | abnormally thin |
| Weight loss | Read v2 | 22A6. | o/e - underweight |
| Weight loss | Read v2 | 22A8. | weight loss from baseline weight |
| Weight loss | Read v2 | R032. | [d]abnormal loss of weight |
| Weight loss | Read v2 | R0348 | [d]underweight |
| Weight loss | Read v2 | R2y4. | [d]cachexia |
| Weight loss | Read v2 | R2y40 | [d]wasting disease |
| Weight loss | Read v2 | R2y4z | [d]cachexia nos |
| Weight loss | CTV3 | .1623 | weight decreasing |
| Weight loss | CTV3 | .1D1A | complaining of weight loss |
| Weight loss | CTV3 | .2224 | o/e - cachexic |
| Weight loss | CTV3 | .2287 | abnormally thin |
| Weight loss | CTV3 | .22A6 | o/e - underweight |
| Weight loss | CTV3 | .22A8 | weight loss from baseline weight |
| Weight loss | CTV3 | .R302 | [d]abnormal loss of weight |
| Weight loss | CTV3 | .RJ4. | [d]cachexia |
| Weight loss | CTV3 | 1623. | weight decreasing |
| Weight loss | CTV3 | 1625. | abnormal weight loss - symptom |
| Weight loss | CTV3 | 1627. | unintentional weight loss |
| Weight loss | CTV3 | 1D1A. | complaining of weight loss |
| Weight loss | CTV3 | 2224. | o/e - cachexic |
| Weight loss | CTV3 | 2287. | abnormally thin |
| Weight loss | CTV3 | 22A6. | o/e - underweight |
| Weight loss | CTV3 | 22A8. | weight loss from baseline weight |
| Weight loss | CTV3 | R032. | [d]abnormal loss of weight |
| Weight loss | CTV3 | R0348 | [d]underweight |
| Weight loss | CTV3 | R2y4. | [d]cachexia |
| Weight loss | CTV3 | R2y40 | [d]wasting disease |
| Weight loss | CTV3 | R2y4z | [d]cachexia nos |
| Weight loss | CTV3 | XE0qb | abnormal weight loss |
| Weight loss | CTV3 | XaIu3 | abnormally thin |
| Weight loss | CTV3 | XaIxC | weight loss from baseline weight |
| Weight loss | CTV3 | XaJM4 | [d]underweight |
| Weight loss | CTV3 | XaKwR | complaining of weight loss |
| Weight loss | CTV3 | XaXTs | unintentional weight loss |
| Abdominal lump | Read v2 | 25J.. | o/e - abdominal mass palpated |
| Abdominal lump | Read v2 | 25J1. | o/e - abd. mass not palpated |
| Abdominal lump | Read v2 | 25J7. | right iliac fossa mass |
| Abdominal lump | Read v2 | 25J8. | o/e left lower abdominal mass |
| Abdominal lump | Read v2 | 25JZ. | o/e - abd. mass palpated nos |
| Abdominal lump | Read v2 | 25K.. | o/e-abdominal mass consistency |
| Abdominal lump | Read v2 | 25K4. | o/e - abdominal mass-pulsatile |
| Abdominal lump | Read v2 | 25L.. | o/e - abdominal mass shape |
| Abdominal lump | Read v2 | 25Q3. | o/e - pr - rectal mass |
| Abdominal lump | Read v2 | 25R3. | o/e - dullness over abd. mass |
| Abdominal lump | Read v2 | 7H2C5 | biopsy of abdominal mass |
| Abdominal lump | Read v2 | J50.. | intestinal obstruction without mention of hernia |
| Abdominal lump | Read v2 | J50y. | other intestinal obstruction |
| Abdominal lump | Read v2 | J50y0 | intestinal adhesions with obstruction |
| Abdominal lump | Read v2 | J50yz | other intestinal obstruction nos |
| Abdominal lump | Read v2 | J50z. | intestinal obstruction nos |
| Abdominal lump | Read v2 | J50z4 | acute intestinal obstruction |
| Abdominal lump | Read v2 | J50z5 | subacute intestinal obstructuon |
| Abdominal lump | Read v2 | J50zz | intestinal obstruction nos |
| Abdominal lump | Read v2 | R093. | [d]swelling, mass or lump within abdomen or pelvis |
| Abdominal lump | Read v2 | R0930 | [d]abdominal swelling |
| Abdominal lump | Read v2 | R0931 | [d]abdominal mass |
| Abdominal lump | Read v2 | R0932 | [d]abdominal lump |
| Abdominal lump | Read v2 | R093z | [d]swelling, mass or lump within abdomen or pelvis |
| Abdominal lump | Read v2 | SP144 | intestinal obstruction as a complication of care n |
| Abdominal lump | CTV3 | .25J1 | o/e - abd. mass not palpated |
| Abdominal lump | CTV3 | .25J7 | right iliac fossa mass |
| Abdominal lump | CTV3 | .25J8 | o/e left lower abdominal mass |
| Abdominal lump | CTV3 | .25JZ | o/e - abd. mass palpated nos |
| Abdominal lump | CTV3 | .25K. | o/e-abdominal mass consistency |
| Abdominal lump | CTV3 | .25K4 | o/e - abdominal mass-pulsatile |
| Abdominal lump | CTV3 | .25L. | o/e - abdominal mass shape |
| Abdominal lump | CTV3 | .25Q3 | o/e - pr - rectal mass |
| Abdominal lump | CTV3 | .25R3 | o/e - dullness over abd. mass |
| Abdominal lump | CTV3 | .I61. | bowel obstruction |
| Abdominal lump | CTV3 | .R731 | [d]abdominal swelling |
| Abdominal lump | CTV3 | .R93. | [d]swelling, mass or lump within abdomen or pelvis |
| Abdominal lump | CTV3 | .R932 | [d]abdominal mass |
| Abdominal lump | CTV3 | .R933 | [d]abdominal lump |
| Abdominal lump | CTV3 | .R93Z | [d]swelling, mass or lump within abdomen or pelvis |
| Abdominal lump | CTV3 | 25J.. | o/e - abdominal mass palpated |
| Abdominal lump | CTV3 | 25J1. | o/e - abd. mass not palpated |
| Abdominal lump | CTV3 | 25J7. | right iliac fossa mass |
| Abdominal lump | CTV3 | 25J8. | o/e left lower abdominal mass |
| Abdominal lump | CTV3 | 25JZ. | o/e - abd. mass palpated nos |
| Abdominal lump | CTV3 | 25K.. | o/e-abdominal mass consistency |
| Abdominal lump | CTV3 | 25K4. | o/e - abdominal mass-pulsatile |
| Abdominal lump | CTV3 | 25L.. | o/e - abdominal mass shape |
| Abdominal lump | CTV3 | 25Q3. | o/e - pr - rectal mass |
| Abdominal lump | CTV3 | 25R3. | o/e - dullness over abd. mass |
| Abdominal lump | CTV3 | 7H2C5 | biopsy of abdominal mass |
| Abdominal lump | CTV3 | J50.. | intestinal obstruction without mention of hernia |
| Abdominal lump | CTV3 | J50y. | other intestinal obstruction |
| Abdominal lump | CTV3 | J50y0 | intestinal adhesions with obstruction |
| Abdominal lump | CTV3 | J50yz | other intestinal obstruction nos |
| Abdominal lump | CTV3 | J50z. | intestinal obstruction nos |
| Abdominal lump | CTV3 | J50z4 | acute intestinal obstruction |
| Abdominal lump | CTV3 | J50z5 | subacute intestinal obstruction |
| Abdominal lump | CTV3 | J50zz | intestinal obstruction nos |
| Abdominal lump | CTV3 | R093. | [d]swelling, mass or lump within abdomen or pelvis |
| Abdominal lump | CTV3 | R0930 | [d]abdominal swelling |
| Abdominal lump | CTV3 | R0931 | [d]abdominal mass |
| Abdominal lump | CTV3 | R0932 | [d]abdominal lump |
| Abdominal lump | CTV3 | R093z | [d]swelling, mass or lump within abdomen or pelvis |
| Abdominal lump | CTV3 | SP144 | intestinal obstruction as a complication of care n |
| Abdominal lump | CTV3 | X305B | bowel obstruction |
| Abdominal lump | CTV3 | X305C | subacute intestinal obstruction nos |
| Abdominal lump | CTV3 | X305D | small bowel obstruction nos |
| Abdominal lump | CTV3 | X305E | large bowel obstruction nos |
| Abdominal lump | CTV3 | X305F | colonic obstruction nos |
| Abdominal lump | CTV3 | XE0aq | other intestinal obstruction |
| Abdominal lump | CTV3 | XE2nV | [d]abdominal mass |
| Abdominal lump | CTV3 | XaDsr | o/e left lower abdominal mass |
| Constipation | Read v2 | 19C.. | constipation |
| Constipation | Read v2 | 19C2. | constipated |
| Constipation | Read v2 | 19CZ. | constipation nos |
| Constipation | Read v2 | J5031 | faecal impaction |
| Constipation | Read v2 | J520. | constipation - functional |
| Constipation | Read v2 | J5200 | acute constipation |
| Constipation | Read v2 | J5201 | chronic constipation with overflow |
| Constipation | Read v2 | J5202 | chronic constipation without overflow |
| Constipation | Read v2 | J5203 | drug induced constipation |
| Constipation | Read v2 | J5204 | chronic constipation |
| Constipation | Read v2 | J520y | other specified constipation |
| Constipation | Read v2 | J520z | constipation nos |
| Constipation | CTV3 | .19C. | constipation |
| Constipation | CTV3 | .19C2 | constipation |
| Constipation | CTV3 | .19CZ | constipation nos |
| Constipation | CTV3 | 19C.. | constipation |
| Constipation | CTV3 | 19C2. | constipated |
| Constipation | CTV3 | 19CZ. | constipation nos |
| Constipation | CTV3 | J5031 | faecal impaction |
| Constipation | CTV3 | J520. | constipation - functional |
| Constipation | CTV3 | J5200 | acute constipation |
| Constipation | CTV3 | J5201 | chronic constipation with overflow |
| Constipation | CTV3 | J5202 | chronic constipation without overflow |
| Constipation | CTV3 | J5203 | drug induced constipation |
| Constipation | CTV3 | J5204 | chronic constipation |
| Constipation | CTV3 | J520y | other specified constipation |
| Constipation | CTV3 | J520z | constipation nos |
| Constipation | CTV3 | X30Bl | chronic constipation |
| Constipation | CTV3 | XE0rD | constipated |
| Constipation | CTV3 | Xa7n2 | drug induced constipation |
| Cough | Read v2 | 1419. | h/o: whooping cough |
| Cough | Read v2 | 171.. | cough |
| Cough | Read v2 | 1712. | dry cough |
| Cough | Read v2 | 1713. | productive cough -clear sputum |
| Cough | Read v2 | 1714. | productive cough -green sputum |
| Cough | Read v2 | 1715. | productive cough-yellow sputum |
| Cough | Read v2 | 1716. | productive cough nos |
| Cough | Read v2 | 1717. | night cough present |
| Cough | Read v2 | 1718. | night cough absent |
| Cough | Read v2 | 1719. | chesty cough |
| Cough | Read v2 | 171A. | chronic cough |
| Cough | Read v2 | 171B. | persistent cough |
| Cough | Read v2 | 171C. | morning cough |
| Cough | Read v2 | 171D. | evening cough |
| Cough | Read v2 | 171E. | unexplained cough |
| Cough | Read v2 | 171F. | cough with fever |
| Cough | Read v2 | 171G. | bovine cough |
| Cough | Read v2 | 171H. | difficulty in coughing up sputum |
| Cough | Read v2 | 171J. | reflux cough |
| Cough | Read v2 | 171K. | barking cough |
| Cough | Read v2 | 171Z. | cough symptom nos |
| Cough | Read v2 | 173B. | nocturnal cough / wheeze |
| Cough | Read v2 | 174.. | hiccough |
| Cough | Read v2 | 1742. | hiccough present |
| Cough | Read v2 | 174Z. | hiccough nos |
| Cough | Read v2 | 1D87. | cough aggravates symptom |
| Cough | Read v2 | 25P5. | o/e-hernia-cough impulse shown |
| Cough | Read v2 | 655.. | whooping cough vaccination |
| Cough | Read v2 | 65VA. | notification of whooping cough |
| Cough | Read v2 | A33.. | whooping cough |
| Cough | Read v2 | A33y. | whooping cough - other specified organism |
| Cough | Read v2 | A33yz | other whooping cough nos |
| Cough | Read v2 | A33z. | whooping cough nos |
| Cough | Read v2 | E2611 | psychogenic cough |
| Cough | Read v2 | E2612 | psychogenic hiccough |
| Cough | Read v2 | H243. | pneumonia with whooping cough |
| Cough | Read v2 | H3101 | smokers' cough |
| Cough | Read v2 | R062. | [d]cough |
| Cough | Read v2 | R0620 | [d]cough syncope |
| Cough | Read v2 | R068. | [d]hiccough |
| Cough | Read v2 | S120A | cough fracture |
| Cough | Read v2 | S1271 | cough fracture of ribs |
| Cough | Read v2 | ZV036 | [v]whooping cough vaccination |
| Cough | Read v2 | ZV74B | [v]screening for whooping cough |
| Cough | CTV3 | .1419 | h/o: whooping cough |
| Cough | CTV3 | .171. | cough |
| Cough | CTV3 | .1712 | dry cough |
| Cough | CTV3 | .1713 | productive cough -clear sputum |
| Cough | CTV3 | .1714 | productive cough -green sputum |
| Cough | CTV3 | .1715 | productive cough-yellow sputum |
| Cough | CTV3 | .1716 | productive cough nos |
| Cough | CTV3 | .1717 | night cough present |
| Cough | CTV3 | .1718 | night cough absent |
| Cough | CTV3 | .1719 | chesty cough |
| Cough | CTV3 | .171A | chronic cough |
| Cough | CTV3 | .171B | persistent cough |
| Cough | CTV3 | .171C | unexplained cough |
| Cough | CTV3 | .171D | evening cough |
| Cough | CTV3 | .171E | morning cough |
| Cough | CTV3 | .171F | cough with fever |
| Cough | CTV3 | .171G | bovine cough |
| Cough | CTV3 | .171H | difficulty in coughing up sputum |
| Cough | CTV3 | .171J | reflux cough |
| Cough | CTV3 | .171K | barking cough |
| Cough | CTV3 | .171Z | cough symptom nos |
| Cough | CTV3 | .173B | nocturnal cough / wheeze |
| Cough | CTV3 | .174. | hiccough |
| Cough | CTV3 | .1742 | hiccough present |
| Cough | CTV3 | .174Z | hiccough nos |
| Cough | CTV3 | .1D87 | cough aggravates symptom |
| Cough | CTV3 | .25P5 | o/e-hernia-cough impulse shown |
| Cough | CTV3 | .655. | whooping cough vaccination |
| Cough | CTV3 | .65VA | notification of whooping cough |
| Cough | CTV3 | .A34. | whooping cough |
| Cough | CTV3 | .H344 | pneumonia with whooping cough |
| Cough | CTV3 | .R62. | [d]cough |
| Cough | CTV3 | .R68. | [d]hiccough |
| Cough | CTV3 | 1419. | h/o: whooping cough |
| Cough | CTV3 | 171.. | cough |
| Cough | CTV3 | 1712. | dry cough |
| Cough | CTV3 | 1713. | productive cough -clear sputum |
| Cough | CTV3 | 1714. | productive cough -green sputum |
| Cough | CTV3 | 1715. | productive cough-yellow sputum |
| Cough | CTV3 | 1716. | productive cough nos |
| Cough | CTV3 | 1717. | night cough present |
| Cough | CTV3 | 1718. | night cough absent |
| Cough | CTV3 | 1719. | chesty cough |
| Cough | CTV3 | 171A. | chronic cough |
| Cough | CTV3 | 171B. | persistent cough |
| Cough | CTV3 | 171C. | morning cough |
| Cough | CTV3 | 171D. | evening cough |
| Cough | CTV3 | 171E. | unexplained cough |
| Cough | CTV3 | 171F. | cough with fever |
| Cough | CTV3 | 171G. | bovine cough |
| Cough | CTV3 | 171H. | difficulty in coughing up sputum |
| Cough | CTV3 | 171J. | reflux cough |
| Cough | CTV3 | 171K. | barking cough |
| Cough | CTV3 | 171Z. | cough symptom nos |
| Cough | CTV3 | 173B. | nocturnal cough / wheeze |
| Cough | CTV3 | 174.. | hiccough |
| Cough | CTV3 | 1742. | hiccough present |
| Cough | CTV3 | 174Z. | hiccough nos |
| Cough | CTV3 | 1D87. | cough aggravates symptom |
| Cough | CTV3 | 25P5. | o/e-hernia-cough impulse shown |
| Cough | CTV3 | 655.. | whooping cough vaccination |
| Cough | CTV3 | 65VA. | notification of whooping cough |
| Cough | CTV3 | A33.. | whooping cough |
| Cough | CTV3 | A33y. | whooping cough - other specified organism |
| Cough | CTV3 | A33yz | other whooping cough nos |
| Cough | CTV3 | A33z. | whooping cough nos |
| Cough | CTV3 | E2611 | psychogenic cough |
| Cough | CTV3 | E2612 | psychogenic hiccough |
| Cough | CTV3 | H243. | pneumonia with whooping cough |
| Cough | CTV3 | H3101 | smokers' cough |
| Cough | CTV3 | R062. | [d]cough |
| Cough | CTV3 | R0620 | [d]cough syncope |
| Cough | CTV3 | R068. | [d]hiccough |
| Cough | CTV3 | S120A | cough fracture |
| Cough | CTV3 | S1271 | cough fracture |
| Cough | CTV3 | X00Ss | somatoform autonomic dysfunction |
| Cough | CTV3 | X76Hy | productive cough |
| Cough | CTV3 | XE0Qw | whooping cough |
| Cough | CTV3 | XE0qn | cough |
| Cough | CTV3 | XE0qo | productive cough nos |
| Cough | CTV3 | XE0qt | hiccoughs |
| Cough | CTV3 | XM0Ch | c/o - cough |
| Cough | CTV3 | Xa2kc | persistent cough |
| Cough | CTV3 | Xa4fN | barking cough |
| Cough | CTV3 | Xa7uA | bovine cough |
| Cough | CTV3 | Xa7uK | difficulty in coughing up sputum |
| Cough | CTV3 | XaFwR | unexplained cough |
| Cough | CTV3 | XaIO1 | cough with fever |
| Cough | CTV3 | XaLCS | reflux cough |
| Cough | CTV3 | ZV036 | [v]whooping cough vaccination |
| Cough | CTV3 | ZV74B | [v]screening for whooping cough |
| Diarrhoea | Read v2 | 19F.. | diarrhoea symptoms |
| Diarrhoea | Read v2 | 19F1. | diarrhoea not present |
| Diarrhoea | Read v2 | 19F2. | diarrhoea |
| Diarrhoea | Read v2 | 19F3. | spurious (overflow) diarrhoea |
| Diarrhoea | Read v2 | 19G.. | diarrhoea and vomiting |
| Diarrhoea | Read v2 | A0740 | diarrhoea due to staphylococcus |
| Diarrhoea | Read v2 | A076. | viral diarrhoea |
| Diarrhoea | Read v2 | A082. | infectious diarrhoea |
| Diarrhoea | Read v2 | A0820 | dysenteric diarrhoea |
| Diarrhoea | Read v2 | A082z | infectious diarrhoea nos |
| Diarrhoea | Read v2 | A083. | diarrhoea of presumed infectious origin |
| Diarrhoea | Read v2 | Ayu0H | [x]diarrhoea and gastroenteritis of presumed infec |
| Diarrhoea | Read v2 | E2643 | psychogenic diarrhoea |
| Diarrhoea | Read v2 | J4... | noninfective diarrhoea |
| Diarrhoea | Read v2 | J43z. | chronic diarrhoea |
| Diarrhoea | Read v2 | J4z.. | presumed noninfectious diarrhoea |
| Diarrhoea | Read v2 | J4zz. | diarrhoea - presumed non-infectious |
| Diarrhoea | Read v2 | J5210 | irritable bowel syndrome with diarrhoea |
| Diarrhoea | Read v2 | J525. | functional diarrhoea |
| Diarrhoea | Read v2 | R0771 | [d] stools loose |
| Diarrhoea | CTV3 | .19F. | diarrhoea symptoms |
| Diarrhoea | CTV3 | .19F1 | diarrhoea not present |
| Diarrhoea | CTV3 | .19F2 | diarrhoea |
| Diarrhoea | CTV3 | .19F3 | spurious diarrhoea - overflow |
| Diarrhoea | CTV3 | .19G. | diarrhoea and vomiting |
| Diarrhoea | CTV3 | .A122 | travellers' diarrhoea |
| Diarrhoea | CTV3 | .A12Z | infectious diarrhoea nos |
| Diarrhoea | CTV3 | .I653 | psychogenic diarrhoea |
| Diarrhoea | CTV3 | 19F.. | diarrhoea symptoms |
| Diarrhoea | CTV3 | 19F1. | diarrhoea not present |
| Diarrhoea | CTV3 | 19F2. | diarrhoea |
| Diarrhoea | CTV3 | 19F3. | spurious diarrhoea - overflow |
| Diarrhoea | CTV3 | 19G.. | diarrhoea and vomiting |
| Diarrhoea | CTV3 | A0740 | diarrhoea due to staphylococcus |
| Diarrhoea | CTV3 | A076. | viral diarrhoea |
| Diarrhoea | CTV3 | A07y0 | viral diarrhoea |
| Diarrhoea | CTV3 | A0803 | infectious gastroenteritis |
| Diarrhoea | CTV3 | A082. | infectious diarrhoea |
| Diarrhoea | CTV3 | A0820 | dysenteric diarrhoea |
| Diarrhoea | CTV3 | A082z | infectious diarrhoea nos |
| Diarrhoea | CTV3 | A083. | diarrhoea of presumed infectious origin |
| Diarrhoea | CTV3 | Ayu0H | [x]diarrhoea and gastroenteritis of presumed infec |
| Diarrhoea | CTV3 | E2643 | spurious diarrhoea |
| Diarrhoea | CTV3 | J4... | noninfective diarrhoea |
| Diarrhoea | CTV3 | J43z. | chronic diarrhoea |
| Diarrhoea | CTV3 | J4z.. | presumed noninfectious diarrhoea |
| Diarrhoea | CTV3 | J4zz. | diarrhoea - presumed non-infectious |
| Diarrhoea | CTV3 | J5210 | irritable bowel syndrome with diarrhoea |
| Diarrhoea | CTV3 | J525. | functional diarrhoea |
| Diarrhoea | CTV3 | Jyu42 | [x]other specified non-infective gastroenteritis a |
| Diarrhoea | CTV3 | R0771 | [d] stools loose |
| Diarrhoea | CTV3 | X30Bn | chronic diarrhoea |
| Diarrhoea | CTV3 | X30Bp | non-infective diarrhoea |
| Diarrhoea | CTV3 | X70GO | travellers' diarrhoea |
| Diarrhoea | CTV3 | X76dE | diarrhoea and vomiting |
| Diarrhoea | CTV3 | X76fC | loose stool |
| Diarrhoea | CTV3 | XE0Qq | diarrhoea of presumed infectious origin |
| Diarrhoea | CTV3 | XE0rN | diarrhoea symptoms |
| Diarrhoea | CTV3 | XE0rO | diarrhoea symptom nos |
| Diarrhoea | CTV3 | XE1Ye | psychogenic diarrhoea |
| Diarrhoea | CTV3 | XM1Mz | diarrhoea and vomiting, symptom |
| Diarrhoea | CTV3 | Xa97X | diarrhoea due to staphylococcus |
| Diarrhoea | CTV3 | XaQbO | functional diarrhoea |
| Pelvic pain | Read v2 | 1A5A. | c/o perineal pain |
| Pelvic pain | Read v2 | N3212 | ischiopubic synchondrosis |
| Pelvic pain | Read v2 | N33A0 | bony pelvic pain |
| Pelvic pain | Read v2 | R090G | [d] perineal pain |
| Pelvic pain | Read v2 | S53.. | groin sprain |
| Pelvic pain | CTV3 | .1A5A | c/o perineal pain |
| Pelvic pain | CTV3 | .P2Q5 | groin strain |
| Pelvic pain | CTV3 | 1A5A. | c/o perineal pain |
| Pelvic pain | CTV3 | N3212 | ischiopubic synchondrosis |
| Pelvic pain | CTV3 | N33A0 | bony pelvic pain |
| Pelvic pain | CTV3 | R090G | [d] perineal pain |
| Pelvic pain | CTV3 | S53.. | groin sprain |
| Pelvic pain | CTV3 | XA0Bu | strain of tendon of adductor longus |
| Pelvic pain | CTV3 | Xa1nC | groin strain |
| Nausea / vomiting | Read v2 | 161.. | appetite symptom |
| Nausea / vomiting | Read v2 | 1612. | appetite loss - anorexia |
| Nausea / vomiting | Read v2 | 1615. | reduced appetite |
| Nausea / vomiting | Read v2 | 161Z. | appetite symptom nos |
| Nausea / vomiting | Read v2 | 1957. | gastric reflux |
| Nausea / vomiting | Read v2 | 1984. | upset stomach |
| Nausea / vomiting | Read v2 | 199.. | vomiting |
| Nausea / vomiting | Read v2 | 1992. | vomiting |
| Nausea / vomiting | Read v2 | 1993. | projectile vomiting |
| Nausea / vomiting | Read v2 | 1994. | vomiting blood - fresh |
| Nausea / vomiting | Read v2 | 1995. | vomiting blood - coffee ground |
| Nausea / vomiting | Read v2 | 1996. | vomiting - bile stained |
| Nausea / vomiting | Read v2 | 199Z. | vomiting nos |
| Nausea / vomiting | Read v2 | 19FZ. | diarrhoea & vomiting, symptom |
| Nausea / vomiting | Read v2 | 4A... | vomit examination |
| Nausea / vomiting | Read v2 | 4A1.. | vomit - o/e, general |
| Nausea / vomiting | Read v2 | 4A1Z. | vomit exam. general nos |
| Nausea / vomiting | Read v2 | 4A2.. | vomit appearance |
| Nausea / vomiting | Read v2 | 4A22. | vomit appearance - abnormal |
| Nausea / vomiting | Read v2 | 4A23. | vomit: frank blood present |
| Nausea / vomiting | Read v2 | 4A24. | vomit: coffee ground |
| Nausea / vomiting | Read v2 | 4A25. | vomit: bilious |
| Nausea / vomiting | Read v2 | 4A26. | vomit: faeculant |
| Nausea / vomiting | Read v2 | 4A27. | vomit: mucous present |
| Nausea / vomiting | Read v2 | 4A2Z. | vomit: appearance nos |
| Nausea / vomiting | Read v2 | 4A3.. | vomit odour |
| Nausea / vomiting | Read v2 | 4A4Z. | vomit ph nos |
| Nausea / vomiting | Read v2 | 4A5.. | vomit occult blood |
| Nausea / vomiting | Read v2 | 4A51. | vomit occult blood positive |
| Nausea / vomiting | Read v2 | 4A5Z. | vomit occult blood nos |
| Nausea / vomiting | Read v2 | 4A6.. | vomit toxicology |
| Nausea / vomiting | Read v2 | 4AZ.. | vomit examination nos |
| Nausea / vomiting | Read v2 | 7N301 | [so]stomach |
| Nausea / vomiting | Read v2 | A78y1 | epidemic vomiting syndrome |
| Nausea / vomiting | Read v2 | E2642 | cyclical vomiting - psychogenic |
| Nausea / vomiting | Read v2 | E2754 | psychogenic vomiting nos |
| Nausea / vomiting | Read v2 | E2756 | non-organic loss of appetite |
| Nausea / vomiting | Read v2 | Eu505 | [x]vomiting associated with other psychological di |
| Nausea / vomiting | Read v2 | Eu50y | [x]psychogenic loss of appetite |
| Nausea / vomiting | Read v2 | J1... | oesophageal, stomach and duodenal diseases |
| Nausea / vomiting | Read v2 | J16.. | disorders of stomach function |
| Nausea / vomiting | Read v2 | J162. | persistent vomiting |
| Nausea / vomiting | Read v2 | J1620 | cyclical vomiting nos |
| Nausea / vomiting | Read v2 | J162z | persistent vomiting nos |
| Nausea / vomiting | Read v2 | J16y. | other specified stomach function disorders |
| Nausea / vomiting | Read v2 | J16y5 | functional vomiting |
| Nausea / vomiting | Read v2 | J16z. | stomach function disorder nos |
| Nausea / vomiting | Read v2 | J680. | vomiting of blood |
| Nausea / vomiting | Read v2 | R0300 | [d]appetite loss |
| Nausea / vomiting | Read v2 | R070. | [d]nausea and vomiting |
| Nausea / vomiting | Read v2 | R0701 | [d]vomiting |
| Nausea / vomiting | Read v2 | R0703 | [d]drug induced vomiting |
| Nausea / vomiting | Read v2 | R0704 | [d]projectile vomiting |
| Nausea / vomiting | Read v2 | R070z | [d]nausea and vomiting nos |
| Nausea / vomiting | CTV3 | .161. | appetite symptom |
| Nausea / vomiting | CTV3 | .1612 | appetite loss - anorexia |
| Nausea / vomiting | CTV3 | .1615 | reduced appetite |
| Nausea / vomiting | CTV3 | .161Z | appetite symptom nos |
| Nausea / vomiting | CTV3 | .1957 | gastric reflux |
| Nausea / vomiting | CTV3 | .199. | vomiting |
| Nausea / vomiting | CTV3 | .1992 | vomiting |
| Nausea / vomiting | CTV3 | .1993 | projectile vomiting |
| Nausea / vomiting | CTV3 | .1994 | vomiting blood - fresh |
| Nausea / vomiting | CTV3 | .1995 | vomiting blood - coffee ground |
| Nausea / vomiting | CTV3 | .1996 | vomiting - bile stained |
| Nausea / vomiting | CTV3 | .199Z | vomiting nos |
| Nausea / vomiting | CTV3 | .19FZ | diarrhoea & vomiting, symptom |
| Nausea / vomiting | CTV3 | .4A.. | vomit examination |
| Nausea / vomiting | CTV3 | .4A1. | vomit - o/e, general |
| Nausea / vomiting | CTV3 | .4A1Z | vomit exam. general nos |
| Nausea / vomiting | CTV3 | .4A2. | vomit appearance |
| Nausea / vomiting | CTV3 | .4A22 | vomit appearance - abnormal |
| Nausea / vomiting | CTV3 | .4A23 | vomit: frank blood present |
| Nausea / vomiting | CTV3 | .4A24 | vomit contains coffee grounds |
| Nausea / vomiting | CTV3 | .4A25 | vomit: bilious |
| Nausea / vomiting | CTV3 | .4A26 | vomit: faeculant |
| Nausea / vomiting | CTV3 | .4A27 | vomit: mucous present |
| Nausea / vomiting | CTV3 | .4A2Z | vomit: appearance nos |
| Nausea / vomiting | CTV3 | .4A3. | vomit odour |
| Nausea / vomiting | CTV3 | .4A4Z | vomit ph nos |
| Nausea / vomiting | CTV3 | .4A5. | vomit occult blood |
| Nausea / vomiting | CTV3 | .4A51 | vomit occult blood positive |
| Nausea / vomiting | CTV3 | .4A5Z | vomit occult blood nos |
| Nausea / vomiting | CTV3 | .4A6. | vomit toxicology |
| Nausea / vomiting | CTV3 | .4AZ. | vomit examination nos |
| Nausea / vomiting | CTV3 | .I2.. | oesophageal, stomach and duodenal diseases |
| Nausea / vomiting | CTV3 | .I26. | disorder of gastric function |
| Nausea / vomiting | CTV3 | .I26Z | stomach function disorder nos |
| Nausea / vomiting | CTV3 | .I781 | haematemesis |
| Nausea / vomiting | CTV3 | .R300 | [d]appetite loss |
| Nausea / vomiting | CTV3 | .R70. | [d]nausea and vomiting |
| Nausea / vomiting | CTV3 | .R701 | [d]vomiting |
| Nausea / vomiting | CTV3 | .R70Z | [d]nausea and vomiting nos |
| Nausea / vomiting | CTV3 | 161.. | appetite symptom |
| Nausea / vomiting | CTV3 | 1612. | appetite loss - anorexia |
| Nausea / vomiting | CTV3 | 1615. | reduced appetite |
| Nausea / vomiting | CTV3 | 161Z. | appetite symptom nos |
| Nausea / vomiting | CTV3 | 1957. | gastric reflux |
| Nausea / vomiting | CTV3 | 1984. | upset stomach |
| Nausea / vomiting | CTV3 | 199.. | vomiting |
| Nausea / vomiting | CTV3 | 1992. | vomiting |
| Nausea / vomiting | CTV3 | 1993. | projectile vomiting |
| Nausea / vomiting | CTV3 | 1994. | vomiting blood - fresh |
| Nausea / vomiting | CTV3 | 1995. | vomiting blood - coffee ground |
| Nausea / vomiting | CTV3 | 1996. | vomiting - bile stained |
| Nausea / vomiting | CTV3 | 199Z. | vomiting nos |
| Nausea / vomiting | CTV3 | 19FZ. | diarrhoea & vomiting, symptom |
| Nausea / vomiting | CTV3 | 4A... | vomit examination |
| Nausea / vomiting | CTV3 | 4A1.. | vomit - o/e, general |
| Nausea / vomiting | CTV3 | 4A1Z. | vomit exam. general nos |
| Nausea / vomiting | CTV3 | 4A2.. | vomit appearance |
| Nausea / vomiting | CTV3 | 4A22. | vomit appearance - abnormal |
| Nausea / vomiting | CTV3 | 4A23. | vomit: frank blood present |
| Nausea / vomiting | CTV3 | 4A24. | vomit contains coffee grounds |
| Nausea / vomiting | CTV3 | 4A25. | vomit: bilious |
| Nausea / vomiting | CTV3 | 4A26. | vomit: faeculant |
| Nausea / vomiting | CTV3 | 4A27. | vomit: mucous present |
| Nausea / vomiting | CTV3 | 4A2Z. | vomit: appearance nos |
| Nausea / vomiting | CTV3 | 4A3.. | vomit odour |
| Nausea / vomiting | CTV3 | 4A4Z. | vomit ph nos |
| Nausea / vomiting | CTV3 | 4A5.. | vomit occult blood |
| Nausea / vomiting | CTV3 | 4A51. | vomit occult blood positive |
| Nausea / vomiting | CTV3 | 4A5Z. | vomit occult blood nos |
| Nausea / vomiting | CTV3 | 4A6.. | vomit toxicology |
| Nausea / vomiting | CTV3 | 4AZ.. | vomit examination nos |
| Nausea / vomiting | CTV3 | 7N301 | stomach |
| Nausea / vomiting | CTV3 | A78y1 | epidemic vomiting syndrome |
| Nausea / vomiting | CTV3 | E2642 | cyclical vomiting - psychogenic |
| Nausea / vomiting | CTV3 | E2754 | psychogenic vomiting nos |
| Nausea / vomiting | CTV3 | E2756 | non-organic loss of appetite |
| Nausea / vomiting | CTV3 | Eu505 | vomiting associated with other psychological distu |
| Nausea / vomiting | CTV3 | Eu50y | [x]psychogenic loss of appetite |
| Nausea / vomiting | CTV3 | J1... | oesophageal, stomach and duodenal diseases |
| Nausea / vomiting | CTV3 | J16.. | disorder of gastric function |
| Nausea / vomiting | CTV3 | J162. | persistent vomiting |
| Nausea / vomiting | CTV3 | J1620 | cyclical vomiting nos |
| Nausea / vomiting | CTV3 | J162z | persistent vomiting nos |
| Nausea / vomiting | CTV3 | J16y. | other specified stomach function disorders |
| Nausea / vomiting | CTV3 | J16y5 | functional vomiting |
| Nausea / vomiting | CTV3 | J16z. | stomach function disorder nos |
| Nausea / vomiting | CTV3 | J680. | haematemesis |
| Nausea / vomiting | CTV3 | R0300 | [d]appetite loss |
| Nausea / vomiting | CTV3 | R070. | [d]nausea and vomiting |
| Nausea / vomiting | CTV3 | R0701 | [d]vomiting |
| Nausea / vomiting | CTV3 | R0703 | [d]drug-induced vomiting |
| Nausea / vomiting | CTV3 | R0704 | [d]projectile vomiting |
| Nausea / vomiting | CTV3 | R070z | [d]nausea and vomiting nos |
| Nausea / vomiting | CTV3 | Ua1iv | reduced appetite |
| Nausea / vomiting | CTV3 | Ua1kQ | gastric reflux |
| Nausea / vomiting | CTV3 | X00Sx | eating disorder |
| Nausea / vomiting | CTV3 | X76cJ | loss of appetite - symptom |
| Nausea / vomiting | CTV3 | X76co | vomiting symptoms |
| Nausea / vomiting | CTV3 | X76cq | haematemesis |
| Nausea / vomiting | CTV3 | XE0aK | oesophageal, stomach and duodenal diseases |
| Nausea / vomiting | CTV3 | XE0aO | gastro-oesophageal reflux disease without oesophag |
| Nausea / vomiting | CTV3 | XE0rA | vomiting |
| Nausea / vomiting | CTV3 | XE0rB | vomiting blood - fresh |
| Nausea / vomiting | CTV3 | XE24f | appetite loss - anorexia |
| Nausea / vomiting | CTV3 | XE26J | vomit: frank blood present |
| Nausea / vomiting | CTV3 | XE2sN | vomit: bilious |
| Nausea / vomiting | CTV3 | XE2sO | vomit: faeculant |
| Nausea / vomiting | CTV3 | XM0Cm | c/o - vomiting |
| Nausea / vomiting | CTV3 | XM1Uu | bilious vomit o/e |
| Nausea / vomiting | CTV3 | XM1Uv | faeculant vomit o/e |
| Nausea / vomiting | CTV3 | XM1Va | blood in vomit o/e |
| Nausea / vomiting | CTV3 | Xa1dj | blood in vomit - symptom |
| Nausea / vomiting | CTV3 | Xa7Uv | vomit contains coffee grounds |
| Nausea / vomiting | CTV3 | XaBMW | [d]projectile vomiting |
| Albumin | Read v2 | 44M4. | Serum albumin |
| Albumin | Read v2 | 44M40 | Serum albumin normal |
| Albumin | Read v2 | 44M41 | Serum albumin low |
| Albumin | Read v2 | 44MI. | Plasma albumin level |
| Albumin | CTV3 | .44M4 | Serum albumin |
| Albumin | CTV3 | .44MI | Plasma albumin level |
| Albumin | CTV3 | 44M4. | Serum albumin |
| Albumin | CTV3 | 44M40 | Serum albumin normal |
| Albumin | CTV3 | 44M41 | Serum albumin low |
| Albumin | CTV3 | 44MI. | Plasma albumin level |
| Albumin | CTV3 | XE2eA | Serum albumin |
| Albumin | CTV3 | XaIRc | Plasma albumin level |
| CRP | Read v2 | 44CC. | Plasma C reactive protein |
| CRP | Read v2 | 44CC0 | C reactive protein normal |
| CRP | Read v2 | 44CC1 | C reactive protein abnormal |
| CRP | Read v2 | 44CS. | Serum C reactive protein level |
| CRP | CTV3 | .44CC | Plasma C reactive protein |
| CRP | CTV3 | .44CS | Serum C reactive protein level |
| CRP | CTV3 | 44CC. | Plasma C reactive protein |
| CRP | CTV3 | 44CC0 | C-reactive protein normal |
| CRP | CTV3 | 44CC1 | C-reactive protein abnormal |
| CRP | CTV3 | 44CS. | Serum C reactive protein level |
| CRP | CTV3 | XE2dy | Plasma C-reactive protein level |
| CRP | CTV3 | XaINL | Serum C reactive protein level |
| ESR | Read v2 | 42B6. | Erythrocyte sedimentation rate |
| ESR | Read v2 | 42B60 | ESR abnormal |
| ESR | Read v2 | 42B61 | ESR low |
| ESR | Read v2 | 42B62 | ESR normal |
| ESR | Read v2 | 42B63 | ESR raised |
| ESR | Read v2 | 42B6z | Erythrocyte sediment rate NOS |
| ESR | CTV3 | .42B6 | Erythrocyte sedimentation rate |
| ESR | CTV3 | 42B6. | Erythrocyte sedimentation rate |
| ESR | CTV3 | 42B60 | ESR abnormal |
| ESR | CTV3 | 42B61 | ESR low |
| ESR | CTV3 | 42B62 | ESR normal |
| ESR | CTV3 | 42B63 | ESR raised |
| ESR | CTV3 | 42B6z | Erythrocyte sediment rate NOS |
| ESR | CTV3 | XE2m7 | Erythrocyte sedimentation rate |
| Ferritin | Read v2 | 42R4. | Serum ferritin |
| Ferritin | Read v2 | 42R41 | Ferritin level low |
| Ferritin | Read v2 | 42R42 | Serum ferritin normal |
| Ferritin | Read v2 | 42R43 | Serum ferritin high |
| Ferritin | Read v2 | 42d4. | Plasma ferritin level |
| Ferritin | CTV3 | .42R4 | Serum ferritin level |
| Ferritin | CTV3 | .42d4 | Plasma ferritin level |
| Ferritin | CTV3 | 42R4. | Serum ferritin |
| Ferritin | CTV3 | 42R41 | Ferritin level low |
| Ferritin | CTV3 | 42R42 | Serum ferritin normal |
| Ferritin | CTV3 | 42R43 | Serum ferritin high |
| Ferritin | CTV3 | 42d4. | Plasma ferritin level |
| Ferritin | CTV3 | XE24r | Serum ferritin level |
| Ferritin | CTV3 | XaIRQ | Serum ferritin high |
| Ferritin | CTV3 | XaItW | Plasma ferritin level |
| Haematocritperc | Read v2 | 425.. | Haematocrit - PCV |
| Haematocritperc | Read v2 | 4251. | Haematocrit - PCV - normal |
| Haematocritperc | Read v2 | 4252. | Haematocrit - borderline high |
| Haematocritperc | Read v2 | 4253. | Haematocrit - PCV - high |
| Haematocritperc | Read v2 | 4254. | Haematocrit - PCV - low |
| Haematocritperc | Read v2 | 4255. | Haematocrit - borderline low |
| Haematocritperc | Read v2 | 4256. | Haematocrit - PCV - abnormal |
| Haematocritperc | Read v2 | 4257. | Packed cell volume |
| Haematocritperc | Read v2 | 4258. | Haematocrit |
| Haematocritperc | Read v2 | 425Z. | Haematocrit - PCV - NOS |
| Haematocritperc | CTV3 | 4251. | Haematocrit - PCV - normal |
| Haematocritperc | CTV3 | 4254. | Haematocrit - PCV - low |
| Haematocritperc | CTV3 | 425Z. | Haematocrit - PCV - NOS |
| Haematocritperc | CTV3 | X76tb | Haematocrit |
| Haematocritperc | CTV3 | X76tc | Packed cell volume |
| Haematocritperc | CTV3 | XE2Zq | Haematocrit - PCV level |
| Haemoglobinconc | Read v2 | 423.. | Haemoglobin estimation |
| Haemoglobinconc | Read v2 | 4234. | Haemoglobin very low |
| Haemoglobinconc | Read v2 | 4235. | Haemoglobin low |
| Haemoglobinconc | Read v2 | 4236. | Haemoglobin borderline low |
| Haemoglobinconc | Read v2 | 4237. | Haemoglobin normal |
| Haemoglobinconc | Read v2 | 4238. | Haemoglobin borderline high |
| Haemoglobinconc | Read v2 | 4239. | Haemoglobin high |
| Haemoglobinconc | Read v2 | 423A. | Haemoglobin very high |
| Haemoglobinconc | Read v2 | 423B. | Haemoglobin abnormal |
| Haemoglobinconc | Read v2 | 423Z. | Haemoglobin estimation NOS |
| Haemoglobinconc | CTV3 | 4234. | Haemoglobin very low |
| Haemoglobinconc | CTV3 | 4235. | Haemoglobin low |
| Haemoglobinconc | CTV3 | 4236. | Haemoglobin borderline low |
| Haemoglobinconc | CTV3 | 4237. | Haemoglobin normal |
| Haemoglobinconc | CTV3 | 4239. | Haemoglobin high |
| Haemoglobinconc | CTV3 | 423B. | Haemoglobin abnormal |
| Haemoglobinconc | CTV3 | 423Z. | Haemoglobin estimation NOS |
| Haemoglobinconc | CTV3 | X76ti | Haemoglobin H inclusion |
| Haemoglobinconc | CTV3 | XE2m6 | Haemoglobin estimation |
| Haemoglobinconc | CTV3 | XM1Vu | Hb estimation |
| Haemoglobinconc | CTV3 | Xa96v | Haemoglobin concentration |
| PV | Read v2 | 42B.. | Plasma viscosity |
| PV | Read v2 | 42B1. | Plasma viscosity normal |
| PV | Read v2 | 42B2. | Plasma visc. borderline raised |
| PV | Read v2 | 42B3. | Plasma viscosity raised |
| PV | Read v2 | 42B4. | Plasma viscosity low |
| PV | Read v2 | 42B5. | Plasma visc. borderline low |
| PV | Read v2 | 42BZ. | Plasma viscosity NOS |
| PV | Read v2 | 5884. | Blood/plasma viscosimetry |
| PV | Read v2 | R108. | [D]Abnormal plasma viscosity |
| PV | CTV3 | .42B. | Plasma viscosity |
| PV | CTV3 | .42B1 | Plasma viscosity normal |
| PV | CTV3 | .42B2 | Plasma visc. borderline raised |
| PV | CTV3 | .42B3 | Plasma viscosity raised |
| PV | CTV3 | .42B4 | Plasma viscosity low |
| PV | CTV3 | .42B5 | Plasma visc. borderline low |
| PV | CTV3 | .42BZ | Plasma viscosity NOS |
| PV | CTV3 | .5884 | Blood/plasma viscosimetry |
| PV | CTV3 | 42B.. | Plasma viscosity |
| PV | CTV3 | 42B1. | Plasma viscosity normal |
| PV | CTV3 | 42B2. | Plasma visc. borderline raised |
| PV | CTV3 | 42B3. | Plasma viscosity raised |
| PV | CTV3 | 42B4. | Plasma viscosity low |
| PV | CTV3 | 42B5. | Plasma visc. borderline low |
| PV | CTV3 | 42BZ. | Plasma viscosity NOS |
| PV | CTV3 | 5884. | Blood/plasma viscosimetry |
| PV | CTV3 | R108. | [D]Abnormal plasma viscosity |
| PV | CTV3 | XE2pd | Plasma viscosity |
| Platelets | Read v2 | 42P.. | Platelet count |
| Platelets | Read v2 | 42P1. | Platelet count normal |
| Platelets | Read v2 | 42P2. | Thrombocytopenia |
| Platelets | Read v2 | 42P3. | Thrombocythaemia |
| Platelets | Read v2 | 42P4. | Platelet count abnormal |
| Platelets | Read v2 | 42PZ. | Platelet count NOS |
| Platelets | CTV3 | .42P. | Platelet count |
| Platelets | CTV3 | .42P1 | Platelet count normal |
| Platelets | CTV3 | .42P2 | Thrombocytopenia |
| Platelets | CTV3 | .42P3 | Thrombocythaemia |
| Platelets | CTV3 | .42P4 | Platelet count abnormal |
| Platelets | CTV3 | .42PZ | Platelet count NOS |
| Platelets | CTV3 | 42P.. | Platelet count |
| Platelets | CTV3 | 42P1. | Platelet count normal |
| Platelets | CTV3 | 42P2. | Thrombocytopenia |
| Platelets | CTV3 | 42P3. | Thrombocythaemia |
| Platelets | CTV3 | 42P4. | Platelet count abnormal |
| Platelets | CTV3 | 42PZ. | Platelet count NOS |
| Platelets | CTV3 | XE24o | Thrombocytopenia |
