# Supplementary material for: Phenotypes and rates of cancer-relevant symptoms and tests in the year before cancer diagnosis in UK Biobank and CPRD Gold
Source: PLOS Digit Health. 2023 Dec 15;2(12):e0000383. doi: 10.1371/journal.pdig.0000383 (PMC10723831; doi:10.1371/journal.pdig.0000383)
Supplement: S3 Table — (DOCX) [file pdig.0000383.s004.docx]

***S3 Table. All feature comparisons between UK Biobank and CPRD.***

| **Cancer site** | **Feature type** | **Feature** | **Rate ratio, UK Biobank vs CPRD** | **(95% CI)** |
| --- | --- | --- | --- | --- |
| All cancers | GP consultations | Total consults | 0.86 | (0.85, 0.87) |
| All cancers | Any relevant symptom | Any relevant symptom | 0.61 | (0.59, 0.63) |
| All cancers | Any relevant symptom | Multiple symptoms | 0.47 | (0.45, 0.50) |
| All cancers | 'Alarm' symptoms | Alarm' symptoms | 0.80 | (0.76, 0.84) |
| All cancers | 'Alarm' symptoms | Abdominal lump | 0.62 | (0.47, 0.83) |
| All cancers | 'Alarm' symptoms | Change in bowel habit | 0.69 | (0.55, 0.87) |
| All cancers | 'Alarm' symptoms | Breast lump | 0.60 | (0.55, 0.66) |
| All cancers | 'Alarm' symptoms | Dysphagia | 0.60 | (0.48, 0.76) |
| All cancers | 'Alarm' symptoms | Haematuria | 1.26 | (1.16, 1.36) |
| All cancers | 'Alarm' symptoms | Haemoptysis | 0.70 | (0.50, 0.98) |
| All cancers | 'Alarm' symptoms | Jaundice | 0.61 | (0.44, 0.85) |
| All cancers | 'Alarm' symptoms | PM bleeding | 0.83 | (0.71, 0.96) |
| All cancers | 'Alarm' symptoms | Rectal bleeding | 0.65 | (0.56, 0.75) |
| All cancers | 'Non-alarm' symptoms | 'Non-alarm' symptoms | 0.54 | (0.52, 0.56) |
| All cancers | 'Non-alarm' symptoms | Abdominal bloating | 0.51 | (0.41, 0.62) |
| All cancers | 'Non-alarm' symptoms | Abdominal pain | 0.52 | (0.48, 0.57) |
| All cancers | 'Non-alarm' symptoms | Constipation | 0.52 | (0.44, 0.60) |
| All cancers | 'Non-alarm' symptoms | Cough | 0.48 | (0.45, 0.52) |
| All cancers | 'Non-alarm' symptoms | Diarrhoea | 0.53 | (0.45, 0.62) |
| All cancers | 'Non-alarm' symptoms | Dyspepsia | 0.80 | (0.72, 0.89) |
| All cancers | 'Non-alarm' symptoms | Dyspnoea | 0.65 | (0.59, 0.71) |
| All cancers | 'Non-alarm' symptoms | Fatigue | 0.57 | (0.49, 0.66) |
| All cancers | 'Non-alarm' symptoms | Night sweats | 0.46 | (0.26, 0.79) |
| All cancers | 'Non-alarm' symptoms | Pelvic pain | 0.75 | (0.38, 1.48) |
| All cancers | 'Non-alarm' symptoms | Nausea / vomiting | 0.38 | (0.31, 0.48) |
| All cancers | 'Non-alarm' symptoms | Weight loss | 0.23 | (0.17, 0.31) |
| All cancers | Blood tests | Any blood test | 0.93 | (0.90, 0.95) |
| All cancers | Blood tests | Albumin | 0.98 | (0.96, 1.01) |
| All cancers | Blood tests | CRP | 0.91 | (0.86, 0.97) |
| All cancers | Blood tests | ESR | 1.03 | (0.97, 1.09) |
| All cancers | Blood tests | PV | 0.72 | (0.60, 0.86) |
| All cancers | Blood tests | Ferritin | 1.43 | (1.35, 1.50) |
| All cancers | Blood tests | Haematocrit % | 0.91 | (0.88, 0.93) |
| All cancers | Blood tests | Haemoglobin | 0.89 | (0.86, 0.91) |
| All cancers | Blood tests | Platelets | 0.90 | (0.88, 0.92) |
| Breast | GP consultations | Total consults | 0.86 | (0.83, 0.88) |
| Breast | Any relevant symptom | Any relevant symptom | 0.59 | (0.55, 0.63) |
| Breast | Any relevant symptom | Multiple symptoms | 0.39 | (0.33, 0.45) |
| Breast | 'Alarm' symptoms | Alarm' symptoms | 0.65 | (0.60, 0.70) |
| Breast | 'Alarm' symptoms | Abdominal lump | Suppressed, small numbers |  |
| Breast | 'Alarm' symptoms | Change in bowel habit | Suppressed, small numbers |  |
| Breast | 'Alarm' symptoms | Breast lump | 0.59 | (0.54, 0.65) |
| Breast | 'Alarm' symptoms | Dysphagia | Suppressed, small numbers |  |
| Breast | 'Alarm' symptoms | Haematuria | 2.18 | (1.63, 2.91) |
| Breast | 'Alarm' symptoms | Haemoptysis | Suppressed, small numbers |  |
| Breast | 'Alarm' symptoms | Jaundice | Suppressed, small numbers |  |
| Breast | 'Alarm' symptoms | PM bleeding | 0.92 | (0.53, 1.58) |
| Breast | 'Alarm' symptoms | Rectal bleeding | Suppressed, small numbers |  |
| Breast | 'Non-alarm' symptoms | 'Non-alarm' symptoms | 0.53 | (0.48, 0.59) |
| Breast | 'Non-alarm' symptoms | Abdominal bloating | 0.58 | (0.34, 0.97) |
| Breast | 'Non-alarm' symptoms | Abdominal pain | 0.55 | (0.44, 0.69) |
| Breast | 'Non-alarm' symptoms | Constipation | 0.55 | (0.34, 0.89) |
| Breast | 'Non-alarm' symptoms | Cough | 0.44 | (0.36, 0.53) |
| Breast | 'Non-alarm' symptoms | Diarrhoea | 0.46 | (0.33, 0.64) |
| Breast | 'Non-alarm' symptoms | Dyspepsia | 0.79 | (0.62, 0.99) |
| Breast | 'Non-alarm' symptoms | Dyspnoea | 0.56 | (0.41, 0.77) |
| Breast | 'Non-alarm' symptoms | Fatigue | 0.60 | (0.42, 0.85) |
| Breast | 'Non-alarm' symptoms | Night sweats | Suppressed, small numbers |  |
| Breast | 'Non-alarm' symptoms | Pelvic pain | Suppressed, small numbers |  |
| Breast | 'Non-alarm' symptoms | Nausea / vomiting | Suppressed, small numbers |  |
| Breast | 'Non-alarm' symptoms | Weight loss | Suppressed, small numbers |  |
| Breast | Blood tests | Any blood test | 0.92 | (0.85, 1.00) |
| Breast | Blood tests | Albumin | 0.97 | (0.91, 1.04) |
| Breast | Blood tests | CRP | 0.94 | (0.75, 1.17) |
| Breast | Blood tests | ESR | 0.91 | (0.74, 1.11) |
| Breast | Blood tests | PV | 0.77 | (0.49, 1.21) |
| Breast | Blood tests | Ferritin | 1.72 | (1.50, 1.99) |
| Breast | Blood tests | Haematocrit % | 0.90 | (0.84, 0.96) |
| Breast | Blood tests | Haemoglobin | 0.88 | (0.82, 0.95) |
| Breast | Blood tests | Platelets | 0.89 | (0.83, 0.96) |
| Prostate | GP consultations | Total consults | 0.89 | (0.87, 0.91) |
| Prostate | Any relevant symptom | Any relevant symptom | 0.59 | (0.53, 0.65) |
| Prostate | Any relevant symptom | Multiple symptoms | 0.45 | (0.37, 0.54) |
| Prostate | 'Alarm' symptoms | Alarm' symptoms | 1.04 | (0.88, 1.24) |
| Prostate | 'Alarm' symptoms | Abdominal lump | Suppressed, small numbers |  |
| Prostate | 'Alarm' symptoms | Change in bowel habit | Suppressed, small numbers |  |
| Prostate | 'Alarm' symptoms | Breast lump | Suppressed, small numbers |  |
| Prostate | 'Alarm' symptoms | Dysphagia | Suppressed, small numbers |  |
| Prostate | 'Alarm' symptoms | Haematuria | 1.17 | (0.96, 1.42) |
| Prostate | 'Alarm' symptoms | Haemoptysis | Suppressed, small numbers |  |
| Prostate | 'Alarm' symptoms | Jaundice | Suppressed, small numbers |  |
| Prostate | 'Alarm' symptoms | PM bleeding | Suppressed, small numbers |  |
| Prostate | 'Alarm' symptoms | Rectal bleeding | 0.88 | (0.56, 1.39) |
| Prostate | 'Non-alarm' symptoms | 'Non-alarm' symptoms | 0.49 | (0.44, 0.55) |
| Prostate | 'Non-alarm' symptoms | Abdominal bloating | Suppressed, small numbers |  |
| Prostate | 'Non-alarm' symptoms | Abdominal pain | 0.49 | (0.37, 0.64) |
| Prostate | 'Non-alarm' symptoms | Constipation | 0.52 | (0.34, 0.82) |
| Prostate | 'Non-alarm' symptoms | Cough | 0.44 | (0.36, 0.54) |
| Prostate | 'Non-alarm' symptoms | Diarrhoea | 0.62 | (0.41, 0.95) |
| Prostate | 'Non-alarm' symptoms | Dyspepsia | 0.71 | (0.51, 0.99) |
| Prostate | 'Non-alarm' symptoms | Dyspnoea | 0.59 | (0.47, 0.73) |
| Prostate | 'Non-alarm' symptoms | Fatigue | 0.46 | (0.32, 0.67) |
| Prostate | 'Non-alarm' symptoms | Night sweats | Suppressed, small numbers |  |
| Prostate | 'Non-alarm' symptoms | Pelvic pain | Suppressed, small numbers |  |
| Prostate | 'Non-alarm' symptoms | Nausea / vomiting | Suppressed, small numbers |  |
| Prostate | 'Non-alarm' symptoms | Weight loss | Suppressed, small numbers |  |
| Prostate | Blood tests | Any blood test | 0.90 | (0.85, 0.95) |
| Prostate | Blood tests | Albumin | 0.94 | (0.89, 0.99) |
| Prostate | Blood tests | CRP | 0.90 | (0.78, 1.03) |
| Prostate | Blood tests | ESR | 0.95 | (0.83, 1.08) |
| Prostate | Blood tests | PV | 0.81 | (0.57, 1.15) |
| Prostate | Blood tests | Ferritin | 1.42 | (1.22, 1.65) |
| Prostate | Blood tests | Haematocrit % | 0.89 | (0.84, 0.94) |
| Prostate | Blood tests | Haemoglobin | 0.87 | (0.82, 0.92) |
| Prostate | Blood tests | Platelets | 0.88 | (0.83, 0.93) |
| Colorectal | GP consultations | Total consults | 0.85 | (0.82, 0.89) |
| Colorectal | Any relevant symptom | Any relevant symptom | 0.55 | (0.50, 0.60) |
| Colorectal | Any relevant symptom | Multiple symptoms | 0.42 | (0.36, 0.48) |
| Colorectal | 'Alarm' symptoms | Alarm' symptoms | 0.71 | (0.61, 0.81) |
| Colorectal | 'Alarm' symptoms | Abdominal lump | Suppressed, small numbers |  |
| Colorectal | 'Alarm' symptoms | Change in bowel habit | 0.67 | (0.48, 0.93) |
| Colorectal | 'Alarm' symptoms | Breast lump | Suppressed, small numbers |  |
| Colorectal | 'Alarm' symptoms | Dysphagia | Suppressed, small numbers |  |
| Colorectal | 'Alarm' symptoms | Haematuria | 2.30 | (1.46, 3.61) |
| Colorectal | 'Alarm' symptoms | Haemoptysis | Suppressed, small numbers |  |
| Colorectal | 'Alarm' symptoms | Jaundice | Suppressed, small numbers |  |
| Colorectal | 'Alarm' symptoms | PM bleeding | Suppressed, small numbers |  |
| Colorectal | 'Alarm' symptoms | Rectal bleeding | 0.63 | (0.53, 0.76) |
| Colorectal | 'Non-alarm' symptoms | 'Non-alarm' symptoms | 0.49 | (0.44, 0.56) |
| Colorectal | 'Non-alarm' symptoms | Abdominal bloating | 0.64 | (0.40, 1.03) |
| Colorectal | 'Non-alarm' symptoms | Abdominal pain | 0.50 | (0.41, 0.62) |
| Colorectal | 'Non-alarm' symptoms | Constipation | 0.47 | (0.35, 0.65) |
| Colorectal | 'Non-alarm' symptoms | Cough | 0.50 | (0.39, 0.63) |
| Colorectal | 'Non-alarm' symptoms | Diarrhoea | 0.32 | (0.23, 0.43) |
| Colorectal | 'Non-alarm' symptoms | Dyspepsia | 0.74 | (0.55, 1.00) |
| Colorectal | 'Non-alarm' symptoms | Dyspnoea | 0.62 | (0.48, 0.79) |
| Colorectal | 'Non-alarm' symptoms | Fatigue | 0.55 | (0.37, 0.82) |
| Colorectal | 'Non-alarm' symptoms | Night sweats | Suppressed, small numbers |  |
| Colorectal | 'Non-alarm' symptoms | Pelvic pain | Suppressed, small numbers |  |
| Colorectal | 'Non-alarm' symptoms | Nausea / vomiting | 0.43 | (0.21, 0.87) |
| Colorectal | 'Non-alarm' symptoms | Weight loss | Suppressed, small numbers |  |
| Colorectal | Blood tests | Any blood test | 0.86 | (0.80, 0.92) |
| Colorectal | Blood tests | Albumin | 0.86 | (0.81, 0.92) |
| Colorectal | Blood tests | CRP | 0.73 | (0.63, 0.83) |
| Colorectal | Blood tests | ESR | 0.93 | (0.79, 1.09) |
| Colorectal | Blood tests | PV | 0.47 | (0.31, 0.70) |
| Colorectal | Blood tests | Ferritin | 1.32 | (1.17, 1.49) |
| Colorectal | Blood tests | Haematocrit % | 0.84 | (0.78, 0.91) |
| Colorectal | Blood tests | Haemoglobin | 0.84 | (0.78, 0.90) |
| Colorectal | Blood tests | Platelets | 0.84 | (0.78, 0.90) |
| Lung | GP consultations | Total consults | 0.87 | (0.84, 0.91) |
| Lung | Any relevant symptom | Any relevant symptom | 0.67 | (0.60, 0.74) |
| Lung | Any relevant symptom | Multiple symptoms | 0.60 | (0.52, 0.69) |
| Lung | 'Alarm' symptoms | Alarm' symptoms | 1.22 | (0.93, 1.58) |
| Lung | 'Alarm' symptoms | Abdominal lump | Suppressed, small numbers |  |
| Lung | 'Alarm' symptoms | Change in bowel habit | Suppressed, small numbers |  |
| Lung | 'Alarm' symptoms | Breast lump | Suppressed, small numbers |  |
| Lung | 'Alarm' symptoms | Dysphagia | Suppressed, small numbers |  |
| Lung | 'Alarm' symptoms | Haematuria | 3.82 | (2.40, 6.09) |
| Lung | 'Alarm' symptoms | Haemoptysis | 0.72 | (0.49, 1.08) |
| Lung | 'Alarm' symptoms | Jaundice | Suppressed, small numbers |  |
| Lung | 'Alarm' symptoms | PM bleeding | Suppressed, small numbers |  |
| Lung | 'Alarm' symptoms | Rectal bleeding | Suppressed, small numbers |  |
| Lung | 'Non-alarm' symptoms | 'Non-alarm' symptoms | 0.62 | (0.55, 0.69) |
| Lung | 'Non-alarm' symptoms | Abdominal bloating | Suppressed, small numbers |  |
| Lung | 'Non-alarm' symptoms | Abdominal pain | 0.53 | (0.38, 0.76) |
| Lung | 'Non-alarm' symptoms | Constipation | 0.44 | (0.25, 0.76) |
| Lung | 'Non-alarm' symptoms | Cough | 0.58 | (0.49, 0.69) |
| Lung | 'Non-alarm' symptoms | Diarrhoea | 0.44 | (0.26, 0.74) |
| Lung | 'Non-alarm' symptoms | Dyspepsia | 1.05 | (0.75, 1.47) |
| Lung | 'Non-alarm' symptoms | Dyspnoea | 0.74 | (0.62, 0.87) |
| Lung | 'Non-alarm' symptoms | Fatigue | 0.77 | (0.50, 1.18) |
| Lung | 'Non-alarm' symptoms | Night sweats | Suppressed, small numbers |  |
| Lung | 'Non-alarm' symptoms | Pelvic pain | Suppressed, small numbers |  |
| Lung | 'Non-alarm' symptoms | Nausea / vomiting | 0.46 | (0.26, 0.81) |
| Lung | 'Non-alarm' symptoms | Weight loss | Suppressed, small numbers |  |
| Lung | Blood tests | Any blood test | 0.97 | (0.88, 1.06) |
| Lung | Blood tests | Albumin | 1.11 | (1.02, 1.21) |
| Lung | Blood tests | CRP | 1.09 | (0.93, 1.28) |
| Lung | Blood tests | ESR | 1.22 | (1.04, 1.43) |
| Lung | Blood tests | PV | 0.93 | (0.59, 1.48) |
| Lung | Blood tests | Ferritin | 1.21 | (1.00, 1.47) |
| Lung | Blood tests | Haematocrit % | 0.93 | (0.85, 1.01) |
| Lung | Blood tests | Haemoglobin | 0.90 | (0.82, 0.98) |
| Lung | Blood tests | Platelets | 0.91 | (0.84, 0.99) |
| Melanoma | GP consultations | Total consults | 0.92 | (0.87, 0.97) |
| Melanoma | Any relevant symptom | Any relevant symptom | 0.66 | (0.53, 0.81) |
| Melanoma | Any relevant symptom | Multiple symptoms | 0.43 | (0.27, 0.69) |
| Melanoma | 'Alarm' symptoms | Alarm' symptoms | 1.24 | (0.75, 2.05) |
| Melanoma | 'Alarm' symptoms | Abdominal lump | Suppressed, small numbers |  |
| Melanoma | 'Alarm' symptoms | Change in bowel habit | Suppressed, small numbers |  |
| Melanoma | 'Alarm' symptoms | Breast lump | Suppressed, small numbers |  |
| Melanoma | 'Alarm' symptoms | Dysphagia | Suppressed, small numbers |  |
| Melanoma | 'Alarm' symptoms | Haematuria | Suppressed, small numbers |  |
| Melanoma | 'Alarm' symptoms | Haemoptysis | Suppressed, small numbers |  |
| Melanoma | 'Alarm' symptoms | Jaundice | Suppressed, small numbers |  |
| Melanoma | 'Alarm' symptoms | PM bleeding | Suppressed, small numbers |  |
| Melanoma | 'Alarm' symptoms | Rectal bleeding | Suppressed, small numbers |  |
| Melanoma | 'Non-alarm' symptoms | 'Non-alarm' symptoms | 0.61 | (0.49, 0.76) |
| Melanoma | 'Non-alarm' symptoms | Abdominal bloating | Suppressed, small numbers |  |
| Melanoma | 'Non-alarm' symptoms | Abdominal pain | 0.87 | (0.56, 1.35) |
| Melanoma | 'Non-alarm' symptoms | Constipation | Suppressed, small numbers |  |
| Melanoma | 'Non-alarm' symptoms | Cough | 0.47 | (0.33, 0.67) |
| Melanoma | 'Non-alarm' symptoms | Diarrhoea | Suppressed, small numbers |  |
| Melanoma | 'Non-alarm' symptoms | Dyspepsia | 0.98 | (0.56, 1.73) |
| Melanoma | 'Non-alarm' symptoms | Dyspnoea | 0.52 | (0.29, 0.94) |
| Melanoma | 'Non-alarm' symptoms | Fatigue | Suppressed, small numbers |  |
| Melanoma | 'Non-alarm' symptoms | Night sweats | Suppressed, small numbers |  |
| Melanoma | 'Non-alarm' symptoms | Pelvic pain | Suppressed, small numbers |  |
| Melanoma | 'Non-alarm' symptoms | Nausea / vomiting | Suppressed, small numbers |  |
| Melanoma | 'Non-alarm' symptoms | Weight loss | Suppressed, small numbers |  |
| Melanoma | Blood tests | Any blood test | 0.91 | (0.81, 1.04) |
| Melanoma | Blood tests | Albumin | 0.96 | (0.86, 1.08) |
| Melanoma | Blood tests | CRP | 0.91 | (0.71, 1.18) |
| Melanoma | Blood tests | ESR | 0.93 | (0.71, 1.21) |
| Melanoma | Blood tests | PV | Suppressed, small numbers |  |
| Melanoma | Blood tests | Ferritin | 1.96 | (1.44, 2.66) |
| Melanoma | Blood tests | Haematocrit % | 0.91 | (0.81, 1.03) |
| Melanoma | Blood tests | Haemoglobin | 0.87 | (0.77, 0.98) |
| Melanoma | Blood tests | Platelets | 0.90 | (0.80, 1.02) |
| NHL | GP consultations | Total consults | 0.82 | (0.77, 0.86) |
| NHL | Any relevant symptom | Any relevant symptom | 0.62 | (0.53, 0.74) |
| NHL | Any relevant symptom | Multiple symptoms | 0.50 | (0.38, 0.66) |
| NHL | 'Alarm' symptoms | Alarm' symptoms | 0.72 | (0.48, 1.08) |
| NHL | 'Alarm' symptoms | Abdominal lump | Suppressed, small numbers |  |
| NHL | 'Alarm' symptoms | Change in bowel habit | Suppressed, small numbers |  |
| NHL | 'Alarm' symptoms | Breast lump | Suppressed, small numbers |  |
| NHL | 'Alarm' symptoms | Dysphagia | Suppressed, small numbers |  |
| NHL | 'Alarm' symptoms | Haematuria | Suppressed, small numbers |  |
| NHL | 'Alarm' symptoms | Haemoptysis | Suppressed, small numbers |  |
| NHL | 'Alarm' symptoms | Jaundice | Suppressed, small numbers |  |
| NHL | 'Alarm' symptoms | PM bleeding | Suppressed, small numbers |  |
| NHL | 'Alarm' symptoms | Rectal bleeding | Suppressed, small numbers |  |
| NHL | 'Non-alarm' symptoms | 'Non-alarm' symptoms | 0.61 | (0.51, 0.73) |
| NHL | 'Non-alarm' symptoms | Abdominal bloating | Suppressed, small numbers |  |
| NHL | 'Non-alarm' symptoms | Abdominal pain | 0.58 | (0.42, 0.81) |
| NHL | 'Non-alarm' symptoms | Constipation | Suppressed, small numbers |  |
| NHL | 'Non-alarm' symptoms | Cough | 0.45 | (0.33, 0.63) |
| NHL | 'Non-alarm' symptoms | Diarrhoea | Suppressed, small numbers |  |
| NHL | 'Non-alarm' symptoms | Dyspepsia | 1.02 | (0.68, 1.52) |
| NHL | 'Non-alarm' symptoms | Dyspnoea | 0.89 | (0.53, 1.49) |
| NHL | 'Non-alarm' symptoms | Fatigue | 0.49 | (0.29, 0.83) |
| NHL | 'Non-alarm' symptoms | Night sweats | Suppressed, small numbers |  |
| NHL | 'Non-alarm' symptoms | Pelvic pain | Suppressed, small numbers |  |
| NHL | 'Non-alarm' symptoms | Nausea / vomiting | Suppressed, small numbers |  |
| NHL | 'Non-alarm' symptoms | Weight loss | Suppressed, small numbers |  |
| NHL | Blood tests | Any blood test | 0.94 | (0.83, 1.06) |
| NHL | Blood tests | Albumin | 1.00 | (0.89, 1.13) |
| NHL | Blood tests | CRP | 0.83 | (0.68, 1.00) |
| NHL | Blood tests | ESR | 1.04 | (0.87, 1.25) |
| NHL | Blood tests | PV | 1.00 | (0.34, 2.99) |
| NHL | Blood tests | Ferritin | 1.42 | (1.14, 1.77) |
| NHL | Blood tests | Haematocrit % | 0.93 | (0.82, 1.05) |
| NHL | Blood tests | Haemoglobin | 0.91 | (0.81, 1.03) |
| NHL | Blood tests | Platelets | 0.93 | (0.82, 1.05) |
| Bladder | GP consultations | Total consults | 0.98 | (0.92, 1.05) |
| Bladder | Any relevant symptom | Any relevant symptom | 0.83 | (0.74, 0.92) |
| Bladder | Any relevant symptom | Multiple symptoms | 0.57 | (0.45, 0.73) |
| Bladder | 'Alarm' symptoms | Alarm' symptoms | 1.01 | (0.90, 1.13) |
| Bladder | 'Alarm' symptoms | Abdominal lump | Suppressed, small numbers |  |
| Bladder | 'Alarm' symptoms | Change in bowel habit | Suppressed, small numbers |  |
| Bladder | 'Alarm' symptoms | Breast lump | Suppressed, small numbers |  |
| Bladder | 'Alarm' symptoms | Dysphagia | Suppressed, small numbers |  |
| Bladder | 'Alarm' symptoms | Haematuria | 1.02 | (0.91, 1.14) |
| Bladder | 'Alarm' symptoms | Haemoptysis | Suppressed, small numbers |  |
| Bladder | 'Alarm' symptoms | Jaundice | Suppressed, small numbers |  |
| Bladder | 'Alarm' symptoms | PM bleeding | Suppressed, small numbers |  |
| Bladder | 'Alarm' symptoms | Rectal bleeding | Suppressed, small numbers |  |
| Bladder | 'Non-alarm' symptoms | 'Non-alarm' symptoms | 0.52 | (0.41, 0.67) |
| Bladder | 'Non-alarm' symptoms | Abdominal bloating | Suppressed, small numbers |  |
| Bladder | 'Non-alarm' symptoms | Abdominal pain | 0.48 | (0.29, 0.80) |
| Bladder | 'Non-alarm' symptoms | Constipation | Suppressed, small numbers |  |
| Bladder | 'Non-alarm' symptoms | Cough | 0.56 | (0.38, 0.83) |
| Bladder | 'Non-alarm' symptoms | Diarrhoea | Suppressed, small numbers |  |
| Bladder | 'Non-alarm' symptoms | Dyspepsia | Suppressed, small numbers |  |
| Bladder | 'Non-alarm' symptoms | Dyspnoea | 0.57 | (0.37, 0.89) |
| Bladder | 'Non-alarm' symptoms | Fatigue | Suppressed, small numbers |  |
| Bladder | 'Non-alarm' symptoms | Night sweats | Suppressed, small numbers |  |
| Bladder | 'Non-alarm' symptoms | Pelvic pain | Suppressed, small numbers |  |
| Bladder | 'Non-alarm' symptoms | Nausea / vomiting | Suppressed, small numbers |  |
| Bladder | 'Non-alarm' symptoms | Weight loss | Suppressed, small numbers |  |
| Bladder | Blood tests | Any blood test | 1.03 | (0.87, 1.20) |
| Bladder | Blood tests | Albumin | 1.07 | (0.95, 1.20) |
| Bladder | Blood tests | CRP | 1.02 | (0.70, 1.48) |
| Bladder | Blood tests | ESR | 1.17 | (0.82, 1.69) |
| Bladder | Blood tests | PV | Suppressed, small numbers |  |
| Bladder | Blood tests | Ferritin | 2.00 | (1.42, 2.81) |
| Bladder | Blood tests | Haematocrit % | 1.02 | (0.89, 1.18) |
| Bladder | Blood tests | Haemoglobin | 1.00 | (0.87, 1.16) |
| Bladder | Blood tests | Platelets | 1.00 | (0.87, 1.15) |
| Uterine | GP consultations | Total consults | 0.88 | (0.82, 0.94) |
| Uterine | Any relevant symptom | Any relevant symptom | 0.72 | (0.63, 0.83) |
| Uterine | Any relevant symptom | Multiple symptoms | 0.56 | (0.41, 0.77) |
| Uterine | 'Alarm' symptoms | Alarm' symptoms | 0.91 | (0.78, 1.06) |
| Uterine | 'Alarm' symptoms | Abdominal lump | Suppressed, small numbers |  |
| Uterine | 'Alarm' symptoms | Change in bowel habit | Suppressed, small numbers |  |
| Uterine | 'Alarm' symptoms | Breast lump | Suppressed, small numbers |  |
| Uterine | 'Alarm' symptoms | Dysphagia | Suppressed, small numbers |  |
| Uterine | 'Alarm' symptoms | Haematuria | 1.38 | (0.86, 2.20) |
| Uterine | 'Alarm' symptoms | Haemoptysis | Suppressed, small numbers |  |
| Uterine | 'Alarm' symptoms | Jaundice | Suppressed, small numbers |  |
| Uterine | 'Alarm' symptoms | PM bleeding | 0.84 | (0.71, 1.00) |
| Uterine | 'Alarm' symptoms | Rectal bleeding | Suppressed, small numbers |  |
| Uterine | 'Non-alarm' symptoms | 'Non-alarm' symptoms | 0.48 | (0.35, 0.65) |
| Uterine | 'Non-alarm' symptoms | Abdominal bloating | Suppressed, small numbers |  |
| Uterine | 'Non-alarm' symptoms | Abdominal pain | Suppressed, small numbers |  |
| Uterine | 'Non-alarm' symptoms | Constipation | Suppressed, small numbers |  |
| Uterine | 'Non-alarm' symptoms | Cough | 0.46 | (0.29, 0.71) |
| Uterine | 'Non-alarm' symptoms | Diarrhoea | Suppressed, small numbers |  |
| Uterine | 'Non-alarm' symptoms | Dyspepsia | Suppressed, small numbers |  |
| Uterine | 'Non-alarm' symptoms | Dyspnoea | Suppressed, small numbers |  |
| Uterine | 'Non-alarm' symptoms | Fatigue | Suppressed, small numbers |  |
| Uterine | 'Non-alarm' symptoms | Night sweats | Suppressed, small numbers |  |
| Uterine | 'Non-alarm' symptoms | Pelvic pain | Suppressed, small numbers |  |
| Uterine | 'Non-alarm' symptoms | Nausea / vomiting | Suppressed, small numbers |  |
| Uterine | 'Non-alarm' symptoms | Weight loss | Suppressed, small numbers |  |
| Uterine | Blood tests | Any blood test | 0.98 | (0.82, 1.17) |
| Uterine | Blood tests | Albumin | 1.02 | (0.87, 1.19) |
| Uterine | Blood tests | CRP | 1.18 | (0.78, 1.79) |
| Uterine | Blood tests | ESR | 0.99 | (0.66, 1.49) |
| Uterine | Blood tests | PV | Suppressed, small numbers |  |
| Uterine | Blood tests | Ferritin | 2.13 | (1.60, 2.84) |
| Uterine | Blood tests | Haematocrit % | 0.92 | (0.78, 1.08) |
| Uterine | Blood tests | Haemoglobin | 0.92 | (0.78, 1.08) |
| Uterine | Blood tests | Platelets | 0.93 | (0.79, 1.10) |
| Kidney | GP consultations | Total consults | 0.86 | (0.80, 0.92) |
| Kidney | Any relevant symptom | Any relevant symptom | 0.60 | (0.50, 0.72) |
| Kidney | Any relevant symptom | Multiple symptoms | 0.63 | (0.47, 0.85) |
| Kidney | 'Alarm' symptoms | Alarm' symptoms | 0.80 | (0.63, 1.02) |
| Kidney | 'Alarm' symptoms | Abdominal lump | Suppressed, small numbers |  |
| Kidney | 'Alarm' symptoms | Change in bowel habit | Suppressed, small numbers |  |
| Kidney | 'Alarm' symptoms | Breast lump | Suppressed, small numbers |  |
| Kidney | 'Alarm' symptoms | Dysphagia | Suppressed, small numbers |  |
| Kidney | 'Alarm' symptoms | Haematuria | 0.79 | (0.60, 1.05) |
| Kidney | 'Alarm' symptoms | Haemoptysis | Suppressed, small numbers |  |
| Kidney | 'Alarm' symptoms | Jaundice | Suppressed, small numbers |  |
| Kidney | 'Alarm' symptoms | PM bleeding | Suppressed, small numbers |  |
| Kidney | 'Alarm' symptoms | Rectal bleeding | Suppressed, small numbers |  |
| Kidney | 'Non-alarm' symptoms | 'Non-alarm' symptoms | 0.51 | (0.40, 0.67) |
| Kidney | 'Non-alarm' symptoms | Abdominal bloating | Suppressed, small numbers |  |
| Kidney | 'Non-alarm' symptoms | Abdominal pain | 0.59 | (0.40, 0.86) |
| Kidney | 'Non-alarm' symptoms | Constipation | Suppressed, small numbers |  |
| Kidney | 'Non-alarm' symptoms | Cough | 0.40 | (0.26, 0.60) |
| Kidney | 'Non-alarm' symptoms | Diarrhoea | Suppressed, small numbers |  |
| Kidney | 'Non-alarm' symptoms | Dyspepsia | 0.76 | (0.40, 1.45) |
| Kidney | 'Non-alarm' symptoms | Dyspnoea | 0.54 | (0.29, 0.97) |
| Kidney | 'Non-alarm' symptoms | Fatigue | Suppressed, small numbers |  |
| Kidney | 'Non-alarm' symptoms | Night sweats | Suppressed, small numbers |  |
| Kidney | 'Non-alarm' symptoms | Pelvic pain | Suppressed, small numbers |  |
| Kidney | 'Non-alarm' symptoms | Nausea / vomiting | Suppressed, small numbers |  |
| Kidney | 'Non-alarm' symptoms | Weight loss | Suppressed, small numbers |  |
| Kidney | Blood tests | Any blood test | 0.91 | (0.77, 1.08) |
| Kidney | Blood tests | Albumin | 1.01 | (0.86, 1.18) |
| Kidney | Blood tests | CRP | 0.98 | (0.73, 1.31) |
| Kidney | Blood tests | ESR | 0.99 | (0.74, 1.34) |
| Kidney | Blood tests | PV | Suppressed, small numbers |  |
| Kidney | Blood tests | Ferritin | 1.32 | (0.97, 1.80) |
| Kidney | Blood tests | Haematocrit % | 0.88 | (0.75, 1.05) |
| Kidney | Blood tests | Haemoglobin | 0.86 | (0.73, 1.01) |
| Kidney | Blood tests | Platelets | 0.88 | (0.75, 1.04) |
| Upper GI | GP consultations | Total consults | 0.88 | (0.82, 0.94) |
| Upper GI | Any relevant symptom | Any relevant symptom | 0.56 | (0.49, 0.64) |
| Upper GI | Any relevant symptom | Multiple symptoms | 0.46 | (0.36, 0.58) |
| Upper GI | 'Alarm' symptoms | Alarm' symptoms | 0.65 | (0.52, 0.81) |
| Upper GI | 'Alarm' symptoms | Abdominal lump | Suppressed, small numbers |  |
| Upper GI | 'Alarm' symptoms | Change in bowel habit | Suppressed, small numbers |  |
| Upper GI | 'Alarm' symptoms | Breast lump | Suppressed, small numbers |  |
| Upper GI | 'Alarm' symptoms | Dysphagia | 0.58 | (0.44, 0.76) |
| Upper GI | 'Alarm' symptoms | Haematuria | Suppressed, small numbers |  |
| Upper GI | 'Alarm' symptoms | Haemoptysis | Suppressed, small numbers |  |
| Upper GI | 'Alarm' symptoms | Jaundice | Suppressed, small numbers |  |
| Upper GI | 'Alarm' symptoms | PM bleeding | Suppressed, small numbers |  |
| Upper GI | 'Alarm' symptoms | Rectal bleeding | Suppressed, small numbers |  |
| Upper GI | 'Non-alarm' symptoms | 'Non-alarm' symptoms | 0.53 | (0.45, 0.64) |
| Upper GI | 'Non-alarm' symptoms | Abdominal bloating | Suppressed, small numbers |  |
| Upper GI | 'Non-alarm' symptoms | Abdominal pain | 0.47 | (0.32, 0.70) |
| Upper GI | 'Non-alarm' symptoms | Constipation | Suppressed, small numbers |  |
| Upper GI | 'Non-alarm' symptoms | Cough | 0.45 | (0.28, 0.70) |
| Upper GI | 'Non-alarm' symptoms | Diarrhoea | Suppressed, small numbers |  |
| Upper GI | 'Non-alarm' symptoms | Dyspepsia | 0.78 | (0.57, 1.09) |
| Upper GI | 'Non-alarm' symptoms | Dyspnoea | 0.63 | (0.39, 1.00) |
| Upper GI | 'Non-alarm' symptoms | Fatigue | Suppressed, small numbers |  |
| Upper GI | 'Non-alarm' symptoms | Night sweats | Suppressed, small numbers |  |
| Upper GI | 'Non-alarm' symptoms | Pelvic pain | Suppressed, small numbers |  |
| Upper GI | 'Non-alarm' symptoms | Nausea / vomiting | Suppressed, small numbers |  |
| Upper GI | 'Non-alarm' symptoms | Weight loss | Suppressed, small numbers |  |
| Upper GI | Blood tests | Any blood test | 0.96 | (0.83, 1.11) |
| Upper GI | Blood tests | Albumin | 1.01 | (0.88, 1.17) |
| Upper GI | Blood tests | CRP | 0.99 | (0.69, 1.41) |
| Upper GI | Blood tests | ESR | 0.93 | (0.66, 1.32) |
| Upper GI | Blood tests | PV | Suppressed, small numbers |  |
| Upper GI | Blood tests | Ferritin | 1.41 | (1.12, 1.77) |
| Upper GI | Blood tests | Haematocrit % | 0.96 | (0.83, 1.10) |
| Upper GI | Blood tests | Haemoglobin | 0.92 | (0.80, 1.06) |
| Upper GI | Blood tests | Platelets | 0.93 | (0.81, 1.07) |
| Other | GP consultations | Total consults | 0.81 | (0.79, 0.83) |
| Other | Any relevant symptom | Any relevant symptom | 0.60 | (0.56, 0.65) |
| Other | Any relevant symptom | Multiple symptoms | 0.49 | (0.43, 0.55) |
| Other | 'Alarm' symptoms | Alarm' symptoms | 0.92 | (0.80, 1.05) |
| Other | 'Alarm' symptoms | Abdominal lump | 0.66 | (0.45, 0.98) |
| Other | 'Alarm' symptoms | Change in bowel habit | 0.69 | (0.43, 1.10) |
| Other | 'Alarm' symptoms | Breast lump | 1.35 | (0.69, 2.63) |
| Other | 'Alarm' symptoms | Dysphagia | 0.45 | (0.21, 0.97) |
| Other | 'Alarm' symptoms | Haematuria | 1.66 | (1.34, 2.05) |
| Other | 'Alarm' symptoms | Haemoptysis | Suppressed, small numbers |  |
| Other | 'Alarm' symptoms | Jaundice | 0.59 | (0.41, 0.85) |
| Other | 'Alarm' symptoms | PM bleeding | 0.63 | (0.40, 1.01) |
| Other | 'Alarm' symptoms | Rectal bleeding | 0.73 | (0.49, 1.09) |
| Other | 'Non-alarm' symptoms | 'Non-alarm' symptoms | 0.54 | (0.50, 0.59) |
| Other | 'Non-alarm' symptoms | Abdominal bloating | 0.44 | (0.32, 0.61) |
| Other | 'Non-alarm' symptoms | Abdominal pain | 0.53 | (0.45, 0.62) |
| Other | 'Non-alarm' symptoms | Constipation | 0.51 | (0.39, 0.68) |
| Other | 'Non-alarm' symptoms | Cough | 0.46 | (0.39, 0.54) |
| Other | 'Non-alarm' symptoms | Diarrhoea | 0.75 | (0.53, 1.06) |
| Other | 'Non-alarm' symptoms | Dyspepsia | 0.73 | (0.60, 0.88) |
| Other | 'Non-alarm' symptoms | Dyspnoea | 0.65 | (0.54, 0.80) |
| Other | 'Non-alarm' symptoms | Fatigue | 0.67 | (0.49, 0.93) |
| Other | 'Non-alarm' symptoms | Night sweats | Suppressed, small numbers |  |
| Other | 'Non-alarm' symptoms | Pelvic pain | Suppressed, small numbers |  |
| Other | 'Non-alarm' symptoms | Nausea / vomiting | 0.40 | (0.28, 0.56) |
| Other | 'Non-alarm' symptoms | Weight loss | 0.22 | (0.13, 0.38) |
| Other | Blood tests | Any blood test | 0.96 | (0.91, 1.01) |
| Other | Blood tests | Albumin | 1.02 | (0.97, 1.07) |
| Other | Blood tests | CRP | 0.94 | (0.85, 1.04) |
| Other | Blood tests | ESR | 1.13 | (1.02, 1.25) |
| Other | Blood tests | PV | 0.85 | (0.59, 1.23) |
| Other | Blood tests | Ferritin | 1.38 | (1.25, 1.53) |
| Other | Blood tests | Haematocrit % | 0.94 | (0.89, 0.99) |
| Other | Blood tests | Haemoglobin | 0.91 | (0.87, 0.96) |
| Other | Blood tests | Platelets | 0.93 | (0.88, 0.97) |
