# Supplementary material for: Phenotypes and rates of cancer-relevant symptoms and tests in the year before cancer diagnosis in UK Biobank and CPRD Gold
Source: PLOS Digit Health. 2023 Dec 15;2(12):e0000383. doi: 10.1371/journal.pdig.0000383 (PMC10723831; doi:10.1371/journal.pdig.0000383)
Supplement: S4 Table — These comparisons relate to all cancer sites combined. (DOCX) [file pdig.0000383.s005.docx]

***S4 Table. All feature comparisons between individual countries in UK Biobank and CPRD. These comparisons relate to all cancer sites combined.***

| **Cancer site** | **Feature type** | **Feature** | **Country** | **Rate ratio, UK Biobank vs CPRD** | **(95% CI)** |
| --- | --- | --- | --- | --- | --- |
| All cancers | GP consultations | Total consults | UKB - English centres | 0.87 | (0.86, 0.88) |
| All cancers | GP consultations | Total consults | UKB - Scottish centres | 0.68 | (0.66, 0.71) |
| All cancers | GP consultations | Total consults | UKB - Welsh centres | 1.01 | (0.98, 1.05) |
| All cancers | Any relevant symptom | Any relevant symptom | UKB - English centres | 0.63 | (0.61, 0.66) |
| All cancers | Any relevant symptom | Any relevant symptom | UKB - Scottish centres | 0.29 | (0.25, 0.33) |
| All cancers | Any relevant symptom | Any relevant symptom | UKB - Welsh centres | 0.84 | (0.78, 0.91) |
| All cancers | Any relevant symptom | Multiple symptoms | UKB - English centres | 0.48 | (0.45, 0.52) |
| All cancers | Any relevant symptom | Multiple symptoms | UKB - Scottish centres | 0.18 | (0.14, 0.23) |
| All cancers | Any relevant symptom | Multiple symptoms | UKB - Welsh centres | 0.75 | (0.65, 0.85) |
| All cancers | 'Alarm' symptoms | 'Alarm' symptoms | UKB - English centres | 0.84 | (0.79, 0.88) |
| All cancers | 'Alarm' symptoms | 'Alarm' symptoms | UKB - Scottish centres | 0.43 | (0.36, 0.52) |
| All cancers | 'Alarm' symptoms | 'Alarm' symptoms | UKB - Welsh centres | 1.03 | (0.90, 1.17) |
| All cancers | 'Alarm' symptoms | Abdominal lump | UKB - English centres | 0.71 | (0.52, 0.97) |
| All cancers | 'Alarm' symptoms | Abdominal lump | UKB - Scottish centres | Suppressed, small numbers |  |
| All cancers | 'Alarm' symptoms | Abdominal lump | UKB - Welsh centres | 0.43 | (0.18, 1.04) |
| All cancers | 'Alarm' symptoms | Change in bowel habit | UKB - English centres | 0.70 | (0.56, 0.88) |
| All cancers | 'Alarm' symptoms | Change in bowel habit | UKB - Scottish centres | Suppressed, small numbers |  |
| All cancers | 'Alarm' symptoms | Change in bowel habit | UKB - Welsh centres | 1.40 | (0.68, 2.85) |
| All cancers | 'Alarm' symptoms | Breast lump | UKB - English centres | 0.62 | (0.56, 0.68) |
| All cancers | 'Alarm' symptoms | Breast lump | UKB - Scottish centres | 0.25 | (0.17, 0.37) |
| All cancers | 'Alarm' symptoms | Breast lump | UKB - Welsh centres | 0.98 | (0.80, 1.20) |
| All cancers | 'Alarm' symptoms | Dysphagia | UKB - English centres | 0.53 | (0.41, 0.69) |
| All cancers | 'Alarm' symptoms | Dysphagia | UKB - Scottish centres | 0.53 | (0.25, 1.12) |
| All cancers | 'Alarm' symptoms | Dysphagia | UKB - Welsh centres | 1.29 | (0.76, 2.19) |
| All cancers | 'Alarm' symptoms | Haematuria | UKB - English centres | 1.36 | (1.25, 1.49) |
| All cancers | 'Alarm' symptoms | Haematuria | UKB - Scottish centres | 0.97 | (0.73, 1.29) |
| All cancers | 'Alarm' symptoms | Haematuria | UKB - Welsh centres | 0.72 | (0.55, 0.94) |
| All cancers | 'Alarm' symptoms | Haemoptysis | UKB - English centres | 0.59 | (0.38, 0.91) |
| All cancers | 'Alarm' symptoms | Haemoptysis | UKB - Scottish centres | 0.73 | (0.34, 1.55) |
| All cancers | 'Alarm' symptoms | Haemoptysis | UKB - Welsh centres | 1.49 | (0.76, 2.89) |
| All cancers | 'Alarm' symptoms | Jaundice | UKB - English centres | 0.53 | (0.37, 0.78) |
| All cancers | 'Alarm' symptoms | Jaundice | UKB - Scottish centres | 0.33 | (0.14, 0.80) |
| All cancers | 'Alarm' symptoms | Jaundice | UKB - Welsh centres | 1.42 | (0.69, 2.92) |
| All cancers | 'Alarm' symptoms | PM bleeding | UKB - English centres | 0.90 | (0.76, 1.06) |
| All cancers | 'Alarm' symptoms | PM bleeding | UKB - Scottish centres | 0.22 | (0.11, 0.46) |
| All cancers | 'Alarm' symptoms | PM bleeding | UKB - Welsh centres | 1.12 | (0.80, 1.56) |
| All cancers | 'Alarm' symptoms | Rectal bleeding | UKB - English centres | 0.63 | (0.54, 0.75) |
| All cancers | 'Alarm' symptoms | Rectal bleeding | UKB - Scottish centres | 0.25 | (0.15, 0.44) |
| All cancers | 'Alarm' symptoms | Rectal bleeding | UKB - Welsh centres | 1.29 | (0.98, 1.71) |
| All cancers | 'Non-alarm' symptoms | 'Non-alarm' symptoms | UKB - English centres | 0.56 | (0.53, 0.59) |
| All cancers | 'Non-alarm' symptoms | 'Non-alarm' symptoms | UKB - Scottish centres | 0.24 | (0.20, 0.28) |
| All cancers | 'Non-alarm' symptoms | 'Non-alarm' symptoms | UKB - Welsh centres | 0.77 | (0.70, 0.85) |
| All cancers | 'Non-alarm' symptoms | Abdominal bloating | UKB - English centres | 0.51 | (0.40, 0.65) |
| All cancers | 'Non-alarm' symptoms | Abdominal bloating | UKB - Scottish centres | Suppressed, small numbers |  |
| All cancers | 'Non-alarm' symptoms | Abdominal bloating | UKB - Welsh centres | 0.92 | (0.59, 1.42) |
| All cancers | 'Non-alarm' symptoms | Abdominal pain | UKB - English centres | 0.53 | (0.48, 0.59) |
| All cancers | 'Non-alarm' symptoms | Abdominal pain | UKB - Scottish centres | 0.13 | (0.08, 0.21) |
| All cancers | 'Non-alarm' symptoms | Abdominal pain | UKB - Welsh centres | 0.90 | (0.74, 1.10) |
| All cancers | 'Non-alarm' symptoms | Constipation | UKB - English centres | 0.58 | (0.49, 0.68) |
| All cancers | 'Non-alarm' symptoms | Constipation | UKB - Scottish centres | 0.22 | (0.12, 0.40) |
| All cancers | 'Non-alarm' symptoms | Constipation | UKB - Welsh centres | 0.41 | (0.26, 0.63) |
| All cancers | 'Non-alarm' symptoms | Cough | UKB - English centres | 0.48 | (0.44, 0.53) |
| All cancers | 'Non-alarm' symptoms | Cough | UKB - Scottish centres | 0.20 | (0.15, 0.28) |
| All cancers | 'Non-alarm' symptoms | Cough | UKB - Welsh centres | 0.83 | (0.71, 0.99) |
| All cancers | 'Non-alarm' symptoms | Diarrhoea | UKB - English centres | 0.58 | (0.48, 0.69) |
| All cancers | 'Non-alarm' symptoms | Diarrhoea | UKB - Scottish centres | 0.14 | (0.07, 0.29) |
| All cancers | 'Non-alarm' symptoms | Diarrhoea | UKB - Welsh centres | 0.66 | (0.41, 1.06) |
| All cancers | 'Non-alarm' symptoms | Dyspepsia | UKB - English centres | 0.86 | (0.77, 0.97) |
| All cancers | 'Non-alarm' symptoms | Dyspepsia | UKB - Scottish centres | 0.39 | (0.27, 0.57) |
| All cancers | 'Non-alarm' symptoms | Dyspepsia | UKB - Welsh centres | 0.84 | (0.63, 1.12) |
| All cancers | 'Non-alarm' symptoms | Dyspnoea | UKB - English centres | 0.68 | (0.62, 0.76) |
| All cancers | 'Non-alarm' symptoms | Dyspnoea | UKB - Scottish centres | 0.47 | (0.37, 0.62) |
| All cancers | 'Non-alarm' symptoms | Dyspnoea | UKB - Welsh centres | 0.62 | (0.48, 0.81) |
| All cancers | 'Non-alarm' symptoms | Fatigue | UKB - English centres | 0.58 | (0.49, 0.69) |
| All cancers | 'Non-alarm' symptoms | Fatigue | UKB - Scottish centres | 0.16 | (0.07, 0.35) |
| All cancers | 'Non-alarm' symptoms | Fatigue | UKB - Welsh centres | 0.98 | (0.72, 1.34) |
| All cancers | 'Non-alarm' symptoms | Night sweats | UKB - English centres | 0.49 | (0.27, 0.88) |
| All cancers | 'Non-alarm' symptoms | Night sweats | UKB - Scottish centres | Suppressed, small numbers |  |
| All cancers | 'Non-alarm' symptoms | Night sweats | UKB - Welsh centres | Suppressed, small numbers |  |
| All cancers | 'Non-alarm' symptoms | Pelvic pain | UKB - English centres | Suppressed, small numbers |  |
| All cancers | 'Non-alarm' symptoms | Pelvic pain | UKB - Scottish centres | Suppressed, small numbers |  |
| All cancers | 'Non-alarm' symptoms | Pelvic pain | UKB - Welsh centres | Suppressed, small numbers |  |
| All cancers | 'Non-alarm' symptoms | Nausea / vomiting | UKB - English centres | 0.35 | (0.27, 0.46) |
| All cancers | 'Non-alarm' symptoms | Nausea / vomiting | UKB - Scottish centres | 0.38 | (0.22, 0.64) |
| All cancers | 'Non-alarm' symptoms | Nausea / vomiting | UKB - Welsh centres | 0.65 | (0.41, 1.03) |
| All cancers | 'Non-alarm' symptoms | Weight loss | UKB - English centres | 0.23 | (0.16, 0.32) |
| All cancers | 'Non-alarm' symptoms | Weight loss | UKB - Scottish centres | 0.18 | (0.06, 0.51) |
| All cancers | 'Non-alarm' symptoms | Weight loss | UKB - Welsh centres | 0.30 | (0.14, 0.65) |
| All cancers | Blood tests | Any blood test | UKB - English centres | 0.96 | (0.93, 0.99) |
| All cancers | Blood tests | Any blood test | UKB - Scottish centres | 0.58 | (0.52, 0.65) |
| All cancers | Blood tests | Any blood test | UKB - Welsh centres | 1.13 | (1.03, 1.23) |
| All cancers | Blood tests | Albumin | UKB - English centres | 1.02 | (1.00, 1.05) |
| All cancers | Blood tests | Albumin | UKB - Scottish centres | 0.52 | (0.46, 0.58) |
| All cancers | Blood tests | Albumin | UKB - Welsh centres | 1.23 | (1.13, 1.33) |
| All cancers | Blood tests | CRP | UKB - English centres | 0.91 | (0.86, 0.97) |
| All cancers | Blood tests | CRP | UKB - Scottish centres | 0.39 | (0.32, 0.47) |
| All cancers | Blood tests | CRP | UKB - Welsh centres | 1.59 | (1.34, 1.87) |
| All cancers | Blood tests | ESR | UKB - English centres | 1.00 | (0.94, 1.06) |
| All cancers | Blood tests | ESR | UKB - Scottish centres | 0.99 | (0.83, 1.17) |
| All cancers | Blood tests | ESR | UKB - Welsh centres | 1.28 | (1.05, 1.55) |
| All cancers | Blood tests | PV | UKB - English centres | 0.79 | (0.66, 0.95) |
| All cancers | Blood tests | PV | UKB - Scottish centres | Suppressed, small numbers |  |
| All cancers | Blood tests | PV | UKB - Welsh centres | 1.05 | (0.56, 1.97) |
| All cancers | Blood tests | Ferritin | UKB - English centres | 1.40 | (1.32, 1.48) |
| All cancers | Blood tests | Ferritin | UKB - Scottish centres | 0.83 | (0.69, 1.00) |
| All cancers | Blood tests | Ferritin | UKB - Welsh centres | 2.38 | (2.05, 2.77) |
| All cancers | Blood tests | Haematocrit % | UKB - English centres | 0.96 | (0.93, 0.98) |
| All cancers | Blood tests | Haematocrit % | UKB - Scottish centres | 0.55 | (0.48, 0.61) |
| All cancers | Blood tests | Haematocrit % | UKB - Welsh centres | 0.99 | (0.91, 1.08) |
| All cancers | Blood tests | Haemoglobin | UKB - English centres | 0.93 | (0.90, 0.95) |
| All cancers | Blood tests | Haemoglobin | UKB - Scottish centres | 0.55 | (0.49, 0.61) |
| All cancers | Blood tests | Haemoglobin | UKB - Welsh centres | 1.00 | (0.92, 1.09) |
| All cancers | Blood tests | Platelets | UKB - English centres | 0.94 | (0.92, 0.97) |
| All cancers | Blood tests | Platelets | UKB - Scottish centres | 0.56 | (0.50, 0.62) |
| All cancers | Blood tests | Platelets | UKB - Welsh centres | 1.01 | (0.93, 1.10) |
