# Supplementary material for: Phenotypes and rates of cancer-relevant symptoms and tests in the year before cancer diagnosis in UK Biobank and CPRD Gold
Source: PLOS Digit Health. 2023 Dec 15;2(12):e0000383. doi: 10.1371/journal.pdig.0000383 (PMC10723831; doi:10.1371/journal.pdig.0000383)

# S1 Text. Plots of cancer-specific UK Biobank-CPRD comparisons

***Fig A. Breast cancer.***


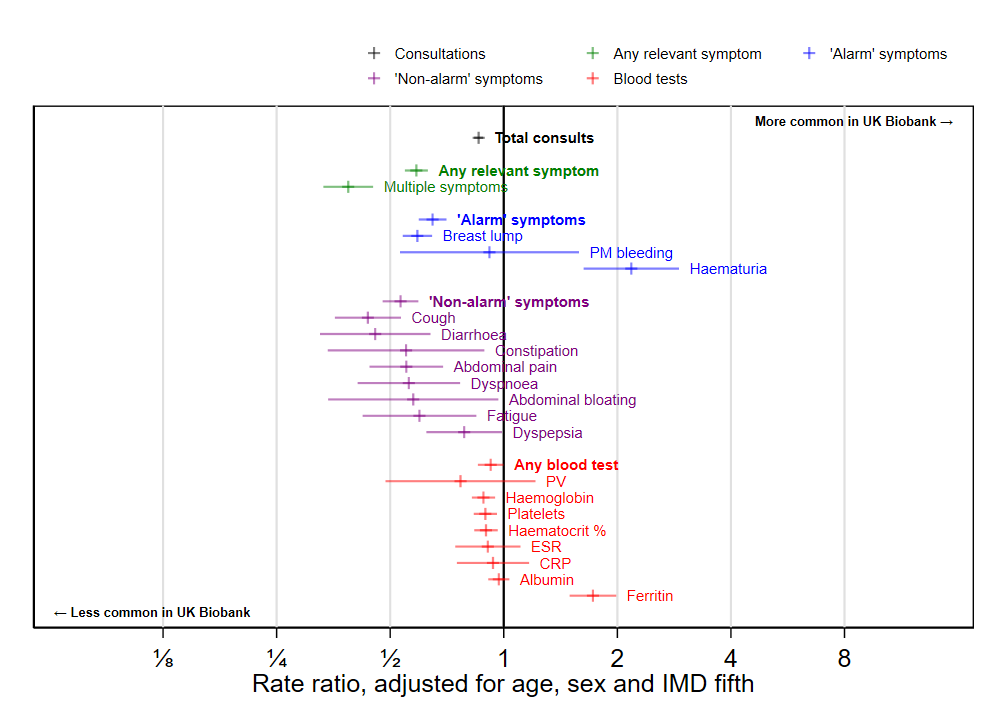


***Fig B. Prostate cancer.***


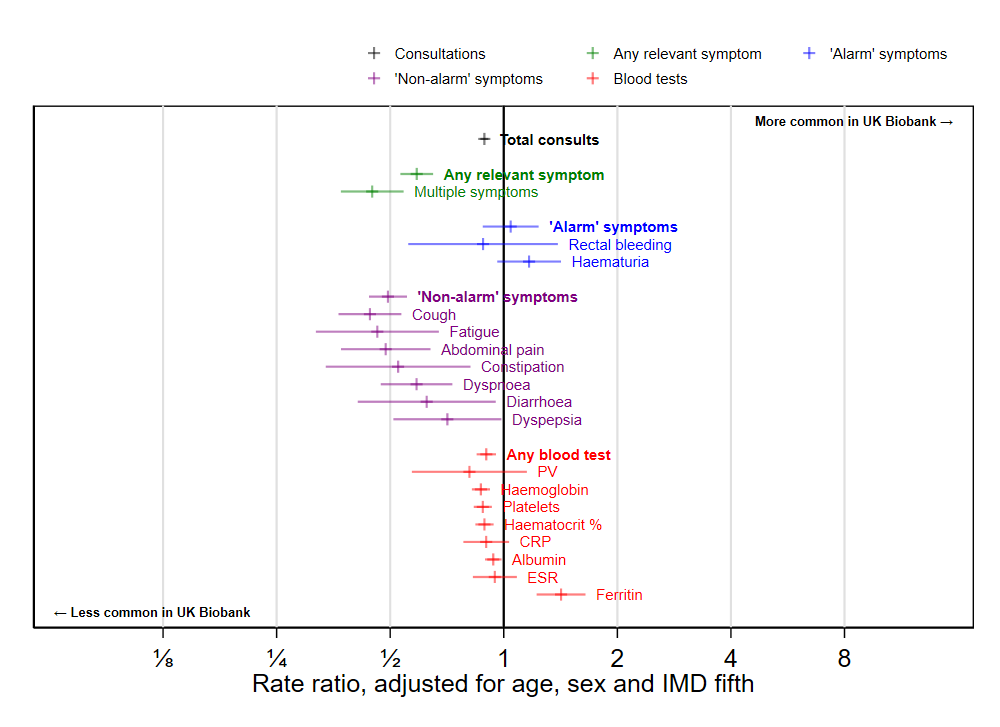


***Fig C. Colorectal cancer.***


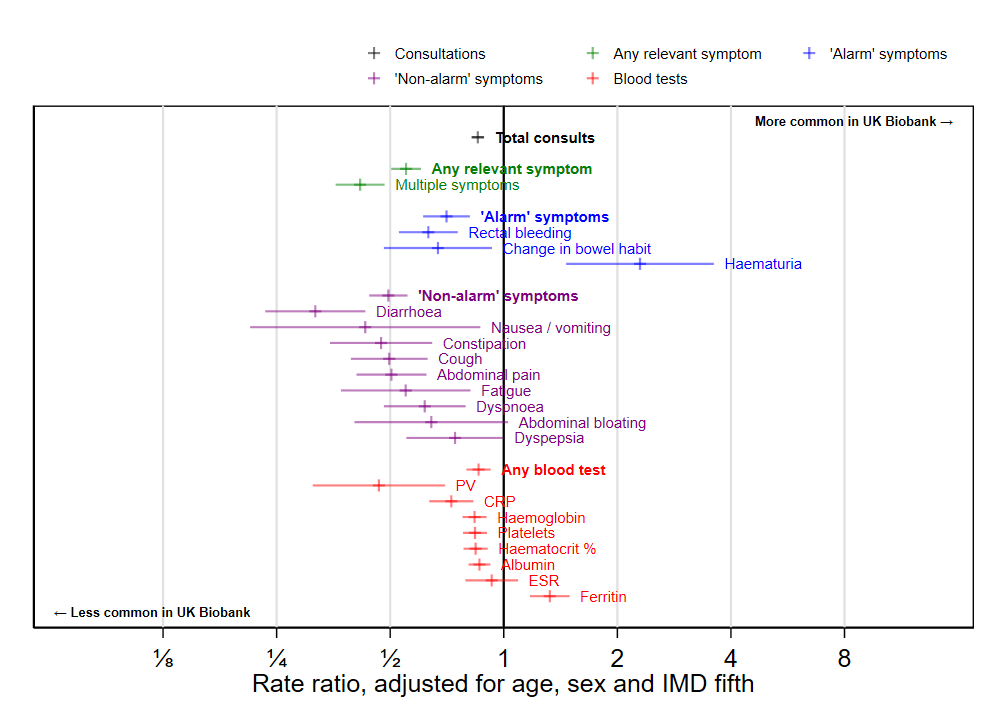


***Fig D. Lung cancer.***


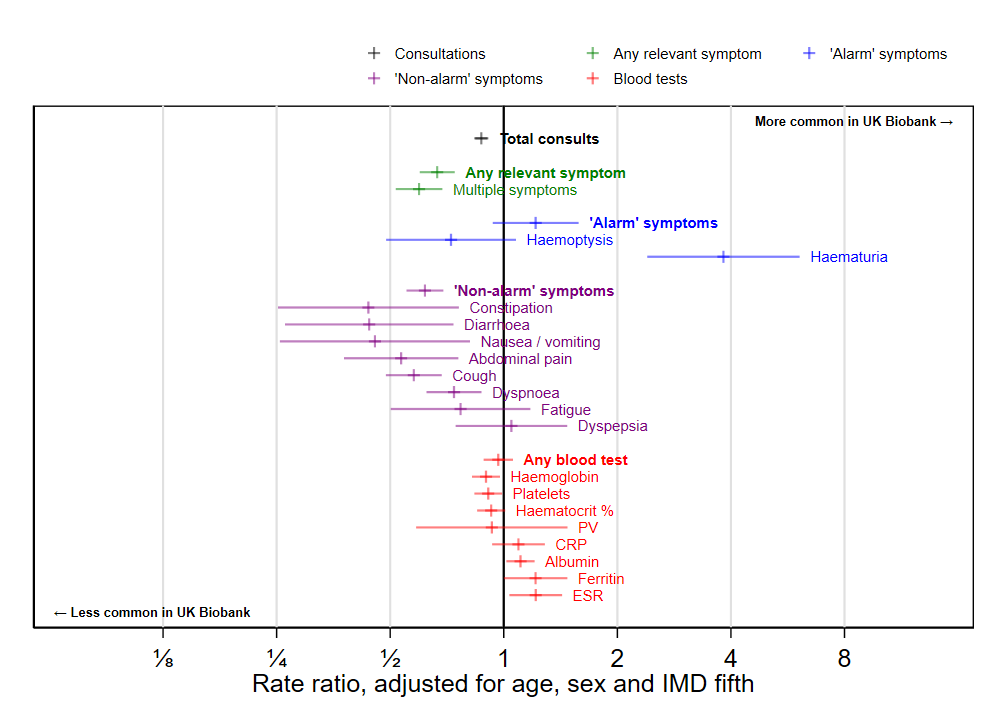


***Fig E. Melanoma.***


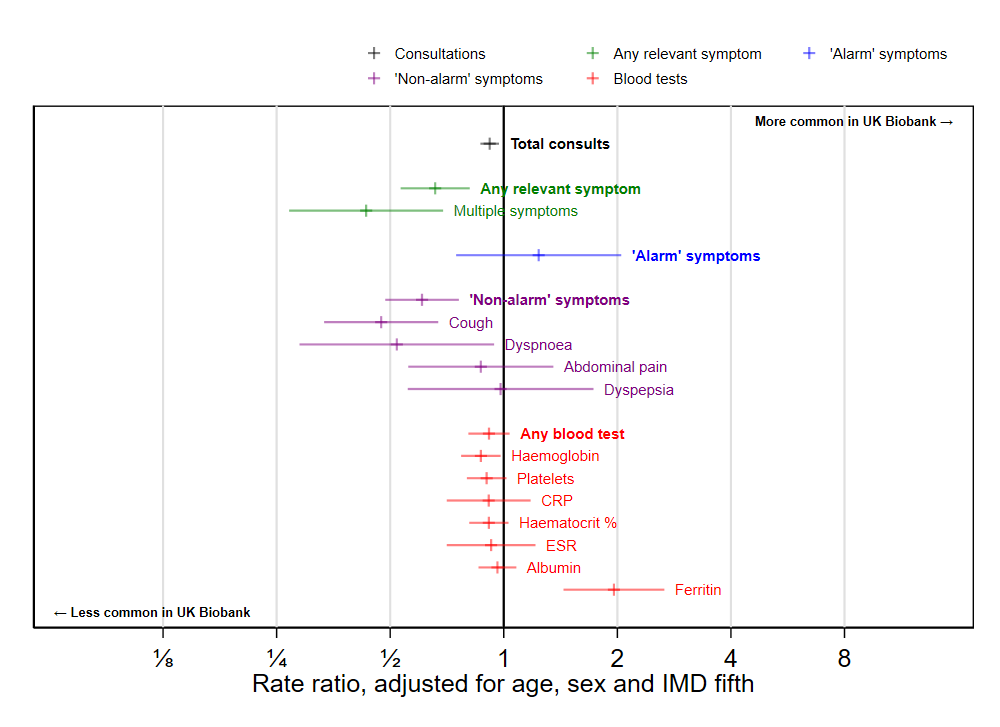


***Fig F. NHL.***


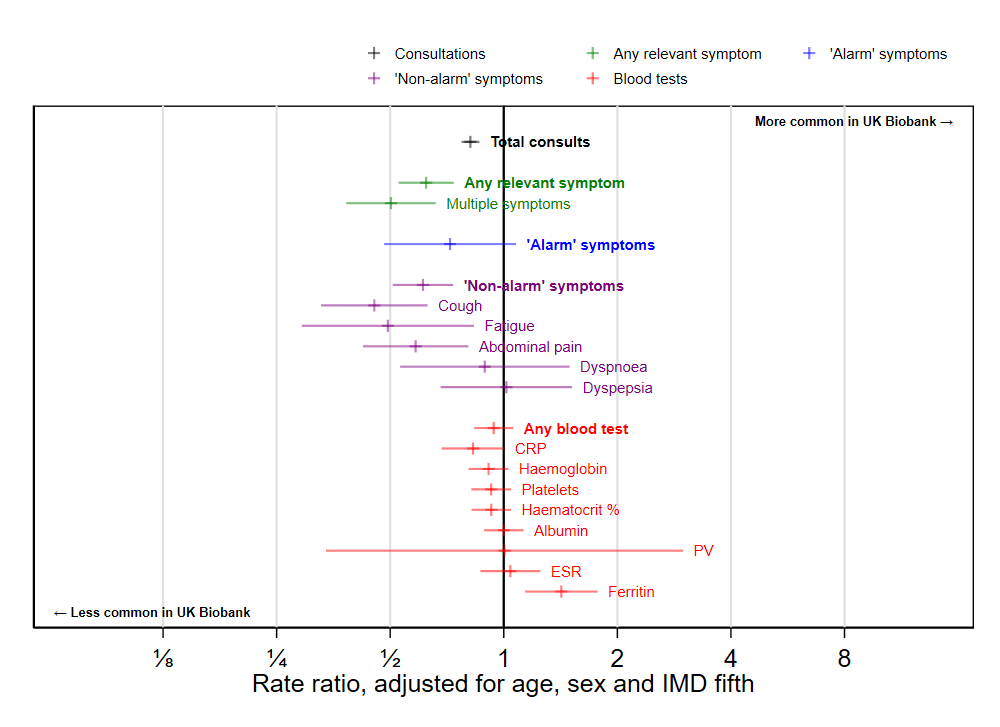


***Fig G. Bladder cancer.***


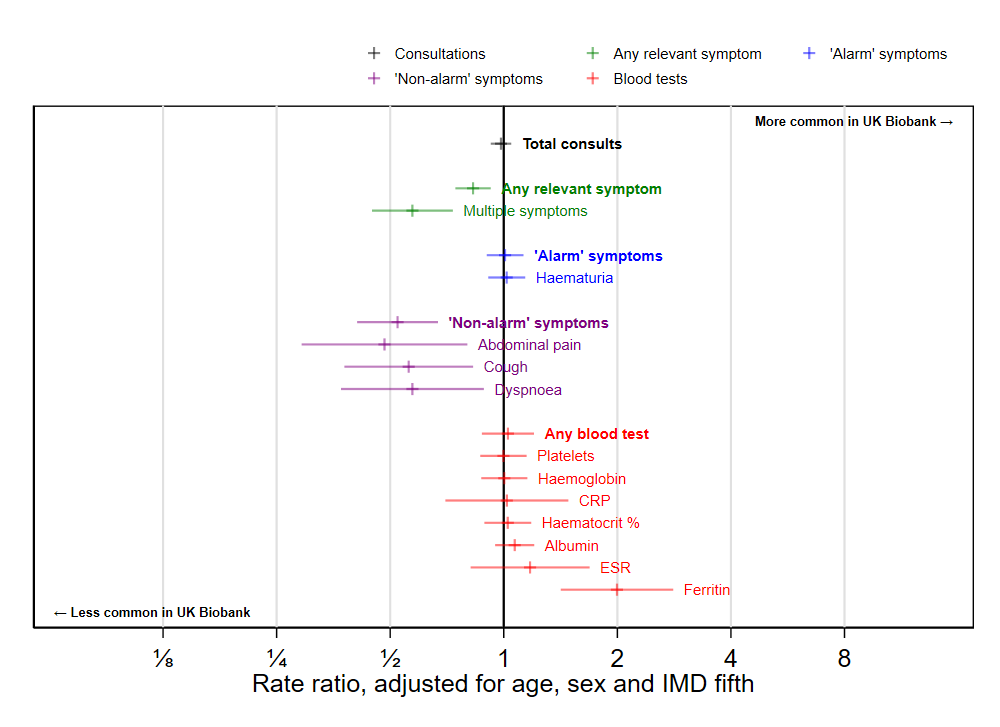


***Fig H. Uterine cancer.***


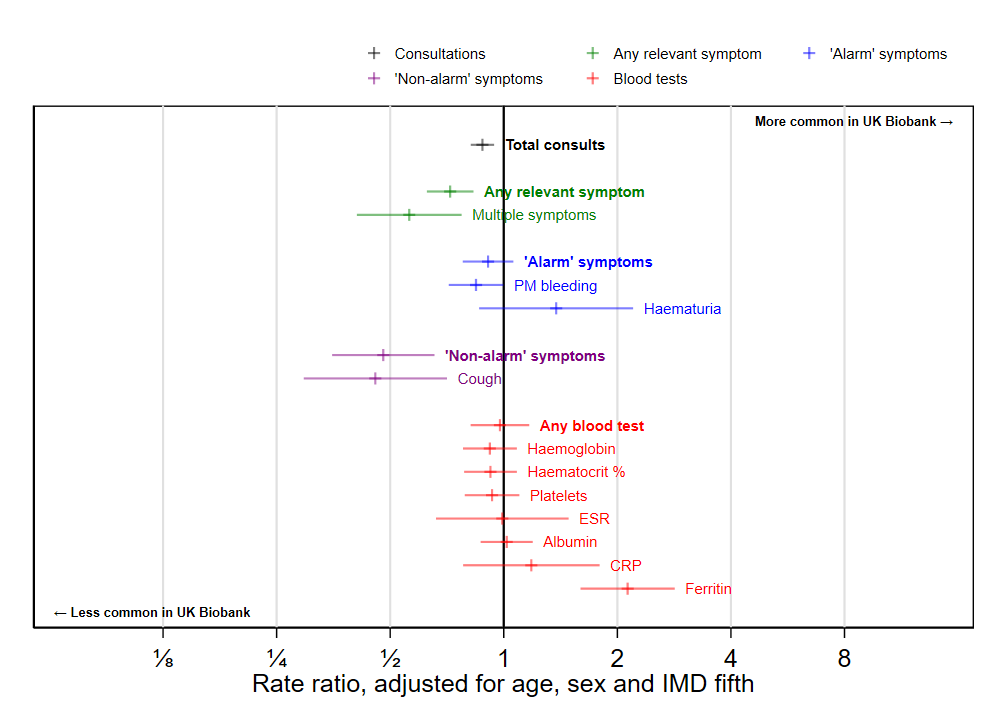


***Fig I. Kidney cancer.***


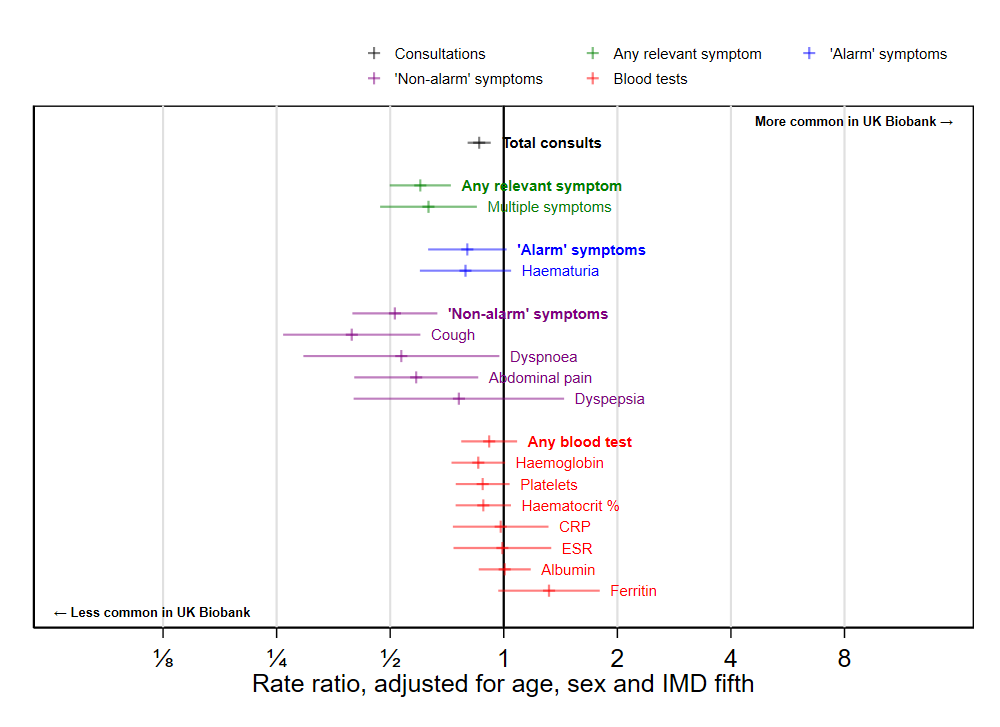


***Fig J. Upper GI cancer.***


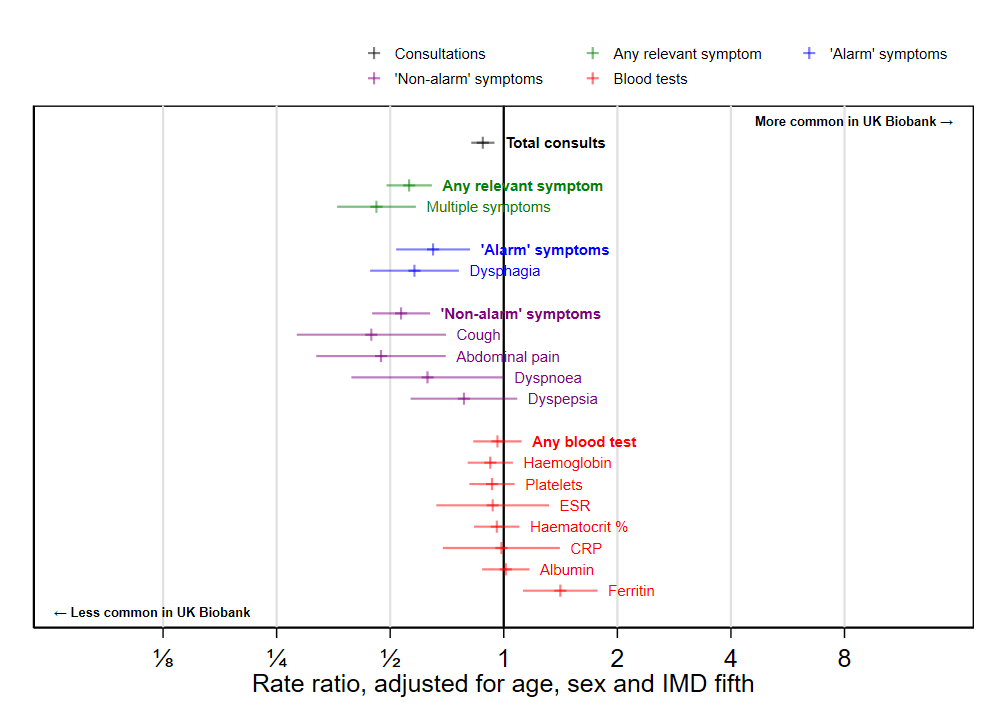


***Fig K. Other cancers.***


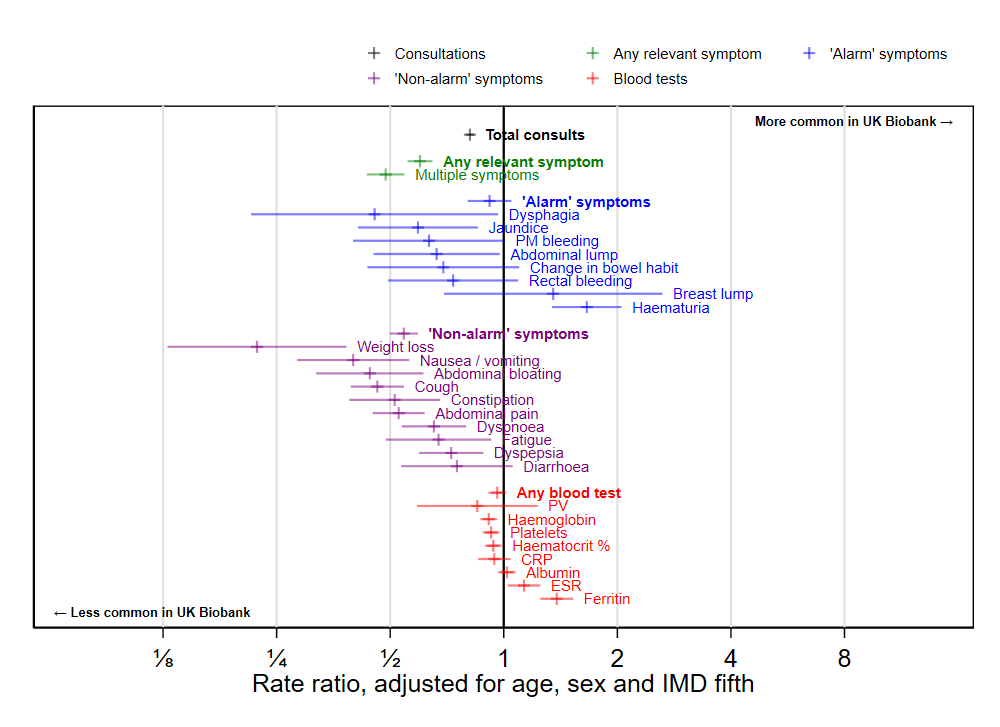

Supplement: S1 Text — Fig A. Breast cancer. Fig B. Prostate cancer. Fig C. Colorectal cancer. Fig D. Lung cancer. Fig E. Melanoma. Fig F. NHL. Fig G. Bladder cancer. Fig H. Uterine cancer. Fig I. Kidney cancer. Fig J. Upper GI cancer. Fig K. Other cancers. (DOCX) [file pdig.0000383.s006.docx]
