# Supplementary material for: Phenotypes and rates of cancer-relevant symptoms and tests in the year before cancer diagnosis in UK Biobank and CPRD Gold
Source: PLOS Digit Health. 2023 Dec 15;2(12):e0000383. doi: 10.1371/journal.pdig.0000383 (PMC10723831; doi:10.1371/journal.pdig.0000383)
Supplement: Attachment — Submitted filename: ukb_cprd_revision_response_final.docx [file pdig.0000383.s007.docx]

| **Editorial comments** |  |
| --- | --- |
| **E1.**  One of the reviewers argues that the alarm symptoms mentioned do not appear to be significant or well-validated. Please make sure that you address this comment to satisfy the reviewer's concern. | We appreciate that the concept of 'alarm' or 'red flag' symptoms for cancer is one that chiefly relates to primary care presentations and conventions about this term vary between different countries. Here we use the term as applied in the UK, to mean symptoms that should on their own trigger an urgent referral for suspected cancer. We feel important to provide additional context as requested, and to make the paper more accessible to non-clinical (e.g., data science) leaders. To that effect we have edited the first two paragraphs in the introduction to read:  Early diagnosis is an important strategy for cancer control associated with improved clinical outcomes and patient experience [1–3]. Over 90% of patients in the UK and over 80% in the US are diagnosed after symptom onset [4–6], and understanding the risk of underlying cancer in symptomatic patients presenting to primary care is critical for achieving earlier diagnosis. Symptoms of possible cancer fall into two groups. First, so-called 'alarm' or 'red-flag' symptoms, for which national guidelines (for example in England and Scotland [7]) indicate that cancer should be suspected, and appropriate referral actions be considered. Overall, the positive predictive value of those symptoms exceeds 3% [8], which is deemed by the UK National Institute of Health and Care Excellence (NICE) to represent an appropriate risk threshold for fast-track investigation. Second, other symptoms with overall predictive value for cancer that is lower than 3%; around half of patients with cancer initially present with such non-specific or vague symptoms [9]. Yet positive predictive values are affected by the underlying risk, and values are generally substantially greater in older than younger individuals and men over women.  Please also see our reply R3-1, below.  In the above text, references 4-6 are new:  4. Swann R, McPhail S, Abel GA, Witt J, Wills L, Hiom S, et al. National Cancer Diagnosis Audits for England 2018 versus 2014: a comparative analysis. Br J Gen Pract. 2023;73: e566–e574. doi:10.3399/BJGP.2022.0268  5. Sarma EA, Kobrin SC, Thompson MJ. A Proposal to Improve the Early Diagnosis of Symptomatic Cancers in the United States. Cancer Prev Res. 2020;13: 715–720. doi:10.1158/1940-6207.CAPR-20-0115  6. Elliss-Brookes L, McPhail S, Ives A, Greenslade M, Shelton J, Hiom S, et al. Routes to diagnosis for cancer - determining the patient journey using multiple routine data sets. Br J Cancer. 2012;107: 1220–1226. doi:10.1038/bjc.2012.408 |
| **E2.**  I would also ask that you explain the context of the study a bit better in the introduction: the positive predictive value of each single symptom from your reference seems to be quite low. What are relevant clinical and policy decisions (e.g., NHS strategies to address this or other clinical guidelines) regarding whether or not these signs and symptoms should be investigated (and how, e.g., stepwise or in a "shotgun" fashion)? | Thank you, we agree with the point. We have provided additional context as following.  Early diagnosis strategies rely on primary care physicians being able to appropriately assess the risk of underlying cancer in symptomatic patients [10]. Several reviews and national guidelines summarise the evidence about the predictive value of common symptoms in either category and related positive predictive values [10–12]. Additionally, risk prediction models with a web interface exist that can support individualised risk prediction conditional on specific symptoms [13]. Currently available evidence chiefly pertains to sources of electronic health records data that do not include information on genetic risk [2,14–18]. The UK Biobank is one of the largest data sources where information from electronic health records and genetic risk are integrated. There is great interest in examining the contribution of genetic predisposition on cancer risk additional to other characteristics using UK Biobank data. Enabling such research requires development of appropriate phenotypes for symptoms of interest, and their distribution described and compared against other sources to guide interpretation. These considerations have motivated our study.  We hope the clarification that many of the symptoms considered in the study would prompt diagnostic investigation in the UK demonstrates the importance of these signs, as will the additional inclusion of a US study describing abdominal pain, rectal bleeding, diarrhoea, and iron deficiency anaemia as red flags for colorectal cancer (i.e. reference 18).  Please see also our replies R3-1 and R3-2, below.  In the above text, references 11, 12 and 19 are new and relate to presence and relevance of ‘alarm’ symptoms for cancer diagnosis in the UK, Denmark and the US.  11. Shapley M, Mansell G, Jordan JL, Jordan KP. Positive predictive values of ≥5% in primary care for cancer: systematic review. Br J Gen Pract. 2010;60: e366–e377. doi:10.3399/bjgp10X515412  12. Huggenberger IK, Andersen JS. Predictive value of the official cancer alarm symptoms in general practice--a systematic review. Dan Med J. 2015;62. Available: http://www.ncbi.nlm.nih.gov/pubmed/26050833  18. Fritz CDL, Otegbeye EE, Zong X, Demb J, Nickel KB, Olsen MA, et al. Red-flag signs and symptoms for earlier diagnosis of early-onset colorectal cancer. JNCI J Natl Cancer Inst. 2023. doi:10.1093/jnci/djad068 |
| **E3.**  Finally, please add limitations to your study and not just discuss the differences between the databases but also the external validity and the clinical and policy implications. | Thank you for highlighting this. We have added paragraphs to the limitations and concluding remarks to address this comment.  First, for external validity, we have added:  Judging external validity of this study is hard due to the selection bias in UK Biobank. Differences between UK Biobank and CPRD will largely be driven by a mix of selection bias in UK Biobank and differences in recording of data due to the different general practice IT systems in use. When comparing other electronic health record datasets based on selected and representative samples, the mix of real differences in health due to selection and apparent differences due to completeness of recording will not be the same; dataset-specific studies will be required.  This then links directly to the key clinical and policy question: what does this mean for risk prediction using EHR. We have added the following to the concluding remarks to address this:  Given the variability in the encoding of symptoms, there is an urgent question about external validity of risk estimates (i.e., whether they can be transported from a given dataset to a population). The results highlight the need for further research of this kind across other sources of primary care electronic health records in the UK and health systems in other countries. Further efforts are needed to develop harmonised phenotypes that can be used across electronic health records datasets, as well as efforts to make use of free-text data from the electronic health record that may contain more complete information on symptoms. The findings support efforts for ensuring equitable participation in contemporary cohort studies collecting genetic, lifestyle and healthcare use data, such as the Our Future Health and All of Us cohorts. |
| **Reviewer #1** |  |
| **R1-1.**  I wonder what was the reasoning behind using both hemoglobin concentration and hematocrit, as they are both indices of anemia? Were other common labs such as serum sodium (SIADH), calcium (NHL, multiple myeloma, lung cancer - hypercalcemia of malignancy), blood urea nitrogen (sensitive marker of GI bleeding), bilirubin (liver metastases, pancreatic cancer) available in both databases for comparison? | Thank you for this comment. We set out to harmonise phenotypes for a range of symptoms and tests, but by necessity only a selection of all possible symptoms and tests was profiled. The reviewer correctly highlights that two tests we compared serve effectively the same purpose. It is worth examining tests that examine aspects of the same underlying physiological process (e.g. haemoglobin and haematocrit both assess presence of anaemia), as it is useful to know if one had more comparable recording than the other (although this did not appear to be the case).  Regarding tests, we agree examining additional tests would have been interesting but we restricted the list due to resource constraints. Our Group has recently published original research on the frequency of use of different common blood tests as part of the diagnostic process of patients with cancer, and from this earlier study we are reassured that the tests we have examined are among the most commonly used ones as part of the diagnostic process of patients subsequently diagnosed with cancer, see Cranfield et al. <https://pubmed.ncbi.nlm.nih.gov/36253112/>.  We now discuss the matter within the limitations section:  Further blood tests of relevance to cancer diagnosis exist that we did not examine, such as calcium and bilirubin. While there would be value in examining recording of further tests, we are reassured that most other blood tests had similar occurrence in both data sources. |
| **R1-2.**  Table 1: Night sweats and especially unintentional weight loss should generally be considered alarm symptoms. | The distinction between alarm and non-alarm symptoms is challenging to draw. Night sweats is a known symptom of many haematological cancers, but it tends to be rare (among cases) and in studies based in primary care (see Shephard E. et al. <https://bjgp.org/content/bjgp/65/634/e289.full.pdf>) it had quite a weak positive predictive value and so we find it hard to call this an alarm symptom. Unexpected weight loss certainly has a strong association with cancer risk overall, but not for individual cancer sites. For this reason, it does not feature in current UK referral clinical practice guidelines which are centred on specific cancers, hence we did not describe it as an alarm symptom in order to be aligned with clinical nomenclature. |
| **R1-3.**  Table 2: Authors can consider presenting this data as a histogram or a bar chart (side-by-side comparison bars between CPRD and UKB) for better visibility. | This is an interesting idea but we think it is useful to keep the information in tabular form. |
| **R1-4.**  Fig 3: Authors may consider moving fig 3 to supplement as it does not add to the readability of the data presented. | Thank you for the suggestion. While we agree to an extent, we would like to keep the figure as it summarises all the comparisons made and reported. |
| **R1-5.**  Fig 4: any possible explanation why Welsh centers were different from that of Scottish and English, i.e. Welsh centers were more similar to CPRD (general UK population)? Is the Welsh population not well-represented in the UKB? | This is unclear to us. One possible conjecture is based on the fact that UK Biobank participants tended to be drawn from the less deprived groups in each country, but we know Wales is more deprived than Scotland or England (<https://pubmed.ncbi.nlm.nih.gov/27852716/>). That is, it may be that a sample that over-represents the less deprived groups in Wales was accidentally quite representative of the UK as a whole. |
| **R1-6.**  [line 266] Sentence should not start with a number: 64%. Instead, it should state: Sixty-four percent. | Thank you, fixed. |
| **R1-7.**  [line 313-314] Please clarify “non-specific exposures” in this context. | You are correct, this was unclear. By “non-specific exposures” we meant non-specific symptoms but we feel this is better addressed by the following paragraph. We have deleted this clause from here and edited the start of the following paragraph to read:  The findings highlight the risk of relying on single sources of EHRs when estimating prevalence or predictive values of less-specific symptoms (e.g., fatigue, abdominal pain) where it appears recording may be most variable.  Which we hope is clearer. |
| **R1-8.**  Although it was not one of the primary aims of this study, it would be interesting to look for associations between nonspecific symptoms and malignancies for which there is no established screening (other than breast, CRC and prostate) and then compare them in aggregate between the two databases. | We agree that this would be a great idea for a future study. |
| **R1-9.**  If numbers allow, would it be possible to further explore different combinations of symptoms and labs and see if there was a specific combination of these covariates that is associated with certain cancer diagnoses in the following year? For example, anemia and bloating together in prediction of GI malignancy as opposed to each of these covariates separately. | We agree with the reviewer that this would be an important piece of work. Combinations of symptoms is frequently challenging to examine in primary care electronic health record data, as they are surprisingly rarely recorded (at least in coded data). For example, in an unpublished study we found that only 4% of patients presenting for one of a range of symptoms had a consultation for a different symptom within 30 days (although these patients were generally at higher cancer risk).  Combinations of symptoms and blood tests is likely easier to examine, as blood tests are relatively frequently ordered in response to a non-specific symptom consultation. We have some ongoing work in this area, and it remains a promising area for future study. |
| In summary, this is a comprehensive study that tried to address an important problem of healthcare data extraction by harmonising EHRs that utilize different coding systems. The authors recognize that coded data offer limited insight and can lead to likely erroneous associations (hematuria and lung cancer). Analyses of unstructured data (free text from clinical notes), where available, by using natural language processing algorithms in the future will most likely be able to uncover a wealth of additional information and this should also be one of the concluding remarks and suggested future directions of this study. | Thank you for this assessment. We tend to agree with the reviewer about the additional possibilities introduced by analysis of free-text data and have made appropriate edits to the concluding remarks, so the relevant sentence now reads:  The results also highlight the need for further research of this kind across other sources of primary care electronic health records in the UK and health systems in other countries. Further efforts are needed to develop harmonised phenotypes that can be used across electronic health records datasets, as well as efforts to make use of free-text data from the electronic health record that may contain more complete information on symptoms.  We would note, however, that in an era of increasing concern around data and privacy, access to free text data in national databases is increasingly difficult. |
| **Reviewer #2** |  |
| Thank you for the opportunity to review this manuscript. Overall I thought the study was very well conducted and clearly presented.  The study clearly shows differences in the recording of symptoms prior to cancer diagnosis between CPRD and UK Biobank. The methods are clearly explained. There are several potential causes for this (other than genuine differences in presentation) that occurred to me when reading the results (UKB participants more likely to be diagnosed through screening, differences in user-interface between systems making coding of symptoms more/less likely, etc.), but each of these were well covered in the discussion (which I thought was excellent and dealt with them in a very balanced way). | Thank you for this kind assessment of our study. |
| I have some minor comments/questions. I realise some of these may/may not be possible with the available data (and don't consider them critical to the integrity of the study).    **R2-1.**  I note that for the UK Biobank population the authors retained those participants from practices using the Vision system. My understanding is that there is (or was?) a data quality issue with these participants (missing data for those participants who had died - bottom of page 12 [here](https://biobank.ndph.ox.ac.uk/showcase/showcase/docs/primary_care_data.pdf)). Can the authors comment on this, and justify their inclusion in the dataset if this is the approach taken. | Many thanks for highlighting this issue, and we are both impressed by the reviewer’s knowledge of the UKB documentation and embarrassed that we had not realised the issue previously. Several of us have read that document multiple times!  Our study only includes patients with linked primary care data, and hence these participants from practices using the Vision system will have been excluded. This means that less healthy participants (i.e., those who have died) from these practices were not included. We might expect this to bias the comparison in the direction of our results, i.e., toward fewer symptoms and tests in the portion of the UKB cohort registered at Vision practices.  We believe this problem will have only a small impact. On the order of 600 patients at Vision practices will have died and we will be missing their data. If all of these patients had cancer and all of them had consulted for one of the symptoms we considered before their diagnosis, then we would have 12,217 (vs 11,594 in our data) cancer patients in UK Biobank with 4,787 (vs 4,164 in our data) or around 39% (vs 36% in our data) having a symptom before diagnosis,. Therefore in this, unrealistically extreme, scenario there will still be considerably lower recording of symptoms before diagnosis in UKB cancer patients than the 54% of cancer patients in CPRD. The real impact will be lower, as many patients who died will not have had cancer, and some of those who died with cancer will not have consulted for symptoms in primary care before diagnosis.  We now explain this in strengths and limitations:  One UK Biobank primary care data provider in England (Vision) did not provide information on patients who died before the data extract; this will have affected around 600 patients meaning there may be a slight underestimate of both cancer cases and symptoms in UK Biobank, but this could at most explain a small fraction of the differences between UKB and CPRD |
| **R2-2.**  Is it possible to identify 'suspected cancer' referrals from either dataset? If so, a comparison of these might help to understand if differences in coding (rather than patients reporting) symptoms is responsible for the differences. (i.e. if suspected cancer referrals were similar, but symptoms lower in UKB, this might suggest that the issue is with lower coding rates within certain systems). As the authors imply, it is a shame that 'route to diagnosis' and stage information is not yet available. | This is an excellent suggestion but we think it would be challenging to implement, and we would still be reliant on completeness of coding of referrals in both datasets. We would aim to revisit this question in a future paper. |
| **R2-3.**  Can the authors confirm their definition of a 'consultation'. Is this any date on which 'event' codes were entered (and therefore are likely to be consultations but may be other contacts/admin events/coding of discharges etc.)? | We can confirm for the reviewer that our definition of a ‘consultation’ is any date on which an event code was entered. This indeed means that coding may occur due to other contacts, admin events, and coding of discharges. Administrative codes, at least, should be relatively rare due to the exclusion of the first year of follow-up, and our choice to focus primarily on symptoms and tests (though we note admin codes could affect the ’total consults’ comparison).  We have added an additional sentence to Table 1 the methods to clarify this:  Consultations for any reason. May include administrative and other non-clinical events. |
| Overall I thought this was an excellent description of an interesting study with a measured interpretation which was very mindful of the potential pitfalls in the data and the multiple possible explanations for the differences observed. | Thank you again for your measured assessment of the study. |
| **Reviewer #3** |  |
| **R3-1.**  The “alarm” symptoms mentioned do not appear to be significant or well-validated, and even such a validation would likely be specific to a given database. It is also unclear how often these non-specific symptoms are present in an age-specific cohort manner. To then take this non-specific set of findings and compare them to another database with non-specific findings seems dubious at best. | We politely but firmly disagree with the statement that ‘alarm’ symptoms do not appear to be significant or well-validated. In addition to national clinical practice guidelines and systematic reviews (please see reply to comment E1 above), we would also like to highlight that Moore and colleagues (<https://doi.org/10.1093/jnci/djad068>) published a detailed and comprehensive review paper based on UK referral guidelines for cancer, which includes supplementary material that documents the PPVs for cancer of various symptoms including those examined in this study, based on a range of primary sources. Prevalence of different symptoms of course varies, but many of these are relatively common. For example, a previous review suggested abdominal pain was present in 2.8% of consultations in primary care (PMID: 24987023); more concerning symptoms such as haemoptysis are of course far less prevalent. We do not see the issue of variation in prevalence by age as a concern for our study, given that our models adjust for age and the age range used is comparable. We agree that some symptoms, such as fatigue in patients under 30 (note, not our study population which included patients 30-75), might not on their own be viewed as likely to be due to underlying cancer, but we would strongly argue that symptoms where 1/20 or 1/10 patients have cancer should be viewed as very significant indeed. To fully disambiguate the matter, we have created a table based on the evidence reviewed and reported by the National Institute for Health and Care Excellence (NICE), the UK healthcare systems guideline developing body, on symptoms of possible cancer, their positive predictive value in primary care populations, and the guideline recommendation. The reference is: Suspected cancer: recognition and referral NICE guideline [NG12] https://www.nice.org.uk/guidance/ng12 **Symptom** \| **PPV** \| **Primary care management / referral guideline from NICE NG12**; Abdominal lump \| \| Ovarian: Urgent referral; Breast lump \| 8.1% (breast, women aged 25-74) \| Breast: Urgent referral women aged >30; Change in bowel habit \| 4.1% (colorectal, all patients) \| Colorectal: Urgent referral patients aged >50 Ovarian: CA125; Dysphagia \| 5.11% (oesophageal, all patients) \| Upper GI: Urgent referral all patients; Haematuria \| 4.7% (bladder, all patients) 3.3% (kidney, all patients) \| Bladder: Urgent referral for patients aged >45 Kidney: Urgent referral for patients aged >45; Haemoptysis \| 3.83% (lung, all patients) \| Lung: Urgent referral patients aged >40; Jaundice \| 12.9% (pancreas, all patients) \| Pancreas: Urgent referral patients aged >40; Post-menopausal bleeding \| 4% (uterine, patients aged >55) \| Uterine: Urgent referral for patients aged >55; Rectal bleed \| 4.4% (colorectal, all patients) \| Colorectal: Urgent referral patients aged >50; Abdominal bloating \| 2.5% (ovarian, women > 40) \| Ovarian: CA125; Abdominal pain \| 2% (colorectal, patients aged 70+) \| Colorectal: Faecal occult blood test (FOBT); Constipation \| 1.3% (colorectal, patients aged 70+) \|; Diarrhoea \| 1.7% (colorectal, patients aged 70+) \|; Dyspepsia \| 0.65% (upper GI, all patients) \|; Dyspnoea \| 1.2% (lung, smokers) \|; Fatigue \| \| Lung: Order CXR smokers aged >40 Ovarian: CA125; Night sweats \| \|; Pelvic pain \| \| Ovarian: CA125; Nausea / vomiting \| 1% (upper GI, patients aged >55) \|; Weight loss \| 2.2% (any cancer, all patients (PMID: 32291391)) \| Lung: Order CXR smokers aged >40 Colorectal: FOBT Ovarian: CA125 We believe the above demonstrate that the study has a firm rationale anchored in both prior evidence and current policy. |
| **R3-2.**  Comparing vague symptoms between two databases seems fraught. It is likely not particularly meaningful, except to make the case that these databases should not be combined for a joint analysis of this type. That could be the only outcome of this analysis, and I am not certain why that should be published in this journal or any journal. | We again disagree. As alluded above, national guidelines on urgent referral for suspected cancer are based on the positive predictive value of different symptoms. The vast majority of evidence supporting these guideline recommendations relates to studies using a single source of EHR data. The implementation of these guidelines is a major undertaking for the health system, and currently in England over 3M people are referred annually to a ‘fast-track’ specialist hospital assessment for suspected cancer, see <https://pubmed.ncbi.nlm.nih.gov/34972804/> Comparing how the prevalence and recording of symptoms may vary between different sources is therefore critical to cross-validate, and if necessary revise, current practice. |
